# HDAC4 influences the DNA damage response and counteracts senescence by assembling with HDAC1/HDAC2 to control H2BK120 acetylation and homology-directed repair

**DOI:** 10.1093/nar/gkae501

**Published:** 2024-06-14

**Authors:** Eros Di Giorgio, Emiliano Dalla, Vanessa Tolotto, Francesca D’Este, Harikrishnareddy Paluvai, Liliana Ranzino, Claudio Brancolini

**Affiliations:** Laboratory of Biochemistry, Department of Medicine, Università degli Studi di Udine, p.le Kolbe 4, 33100 Udine, Italy; Laboratory of Epigenomics, Department of Medicine, Università degli Studi di Udine, p.le Kolbe 4, 33100 Udine, Italy; Laboratory of Epigenomics, Department of Medicine, Università degli Studi di Udine, p.le Kolbe 4, 33100 Udine, Italy; Laboratory of Biochemistry, Department of Medicine, Università degli Studi di Udine, p.le Kolbe 4, 33100 Udine, Italy; Laboratory of Epigenomics, Department of Medicine, Università degli Studi di Udine, p.le Kolbe 4, 33100 Udine, Italy; Laboratory of Epigenomics, Department of Medicine, Università degli Studi di Udine, p.le Kolbe 4, 33100 Udine, Italy; Laboratory of Epigenomics, Department of Medicine, Università degli Studi di Udine, p.le Kolbe 4, 33100 Udine, Italy; Laboratory of Epigenomics, Department of Medicine, Università degli Studi di Udine, p.le Kolbe 4, 33100 Udine, Italy

## Abstract

Access to DNA is the first level of control in regulating gene transcription, a control that is also critical for maintaining DNA integrity. Cellular senescence is characterized by profound transcriptional rearrangements and accumulation of DNA lesions. Here, we discovered an epigenetic complex between HDAC4 and HDAC1/HDAC2 that is involved in the erase of H2BK120 acetylation. The HDAC4/HDAC1/HDAC2 complex modulates the efficiency of DNA repair by homologous recombination, through dynamic deacetylation of H2BK120. Deficiency of HDAC4 leads to accumulation of H2BK120ac, impaired recruitment of BRCA1 and CtIP to the site of lesions, accumulation of damaged DNA and senescence. In senescent cells this complex is disassembled because of increased proteasomal degradation of HDAC4. Forced expression of HDAC4 during RAS-induced senescence reduces the genomic spread of γH2AX. It also affects H2BK120ac levels, which are increased in DNA-damaged regions that accumulate during RAS-induced senescence. In summary, degradation of HDAC4 during senescence causes the accumulation of damaged DNA and contributes to the activation of the transcriptional program controlled by super-enhancers that maintains senescence.

## Graphical Abstract

**Graphical Abstract:**
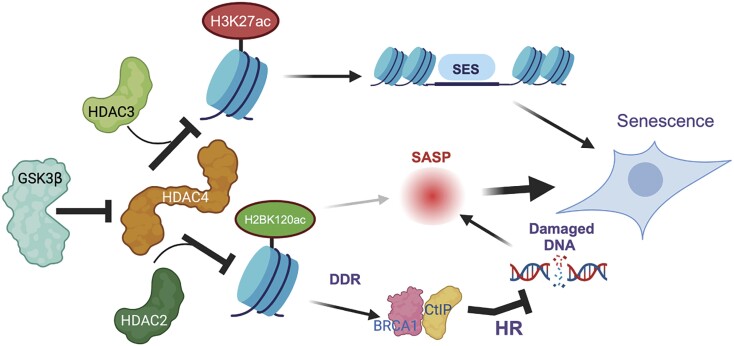

## Introduction

Cellular senescence is a complex cellular state that is maintained by the activation of various genetic programs, to achieve permanent cell cycle arrest and sculpture the microenvironment (1). Several extrinsic and intrinsic conditions/stressors can promote the onset of senescence. Telomere shortening during replicative senescence (RS), DNA damage as induced by oxidative stress, drugs or during oncogene-induced senescence (OIS), and specific developmental programs can trigger different forms of senescence (2,3). The permanent cell cycle arrest seen in all these forms of senescence depends in each case on the upregulation of inhibitors of cyclin-dependent kinases (CDKs) CDKN1A/p21 and CDKN2A/p16 (4). Another genetic program that is involved in several, but not all forms of senescence is the senescence-associated secretory phenotype (SASP), which is based on the transcription of cytokines, chemokines, growth factors and components of the extracellular matrix. This pro-inflammatory secretome is responsible for several of the effects of senescence in tissues and organs (5).

The genetic programs controlling senescence are supervised by extensive restructuring of the epigenome and 3D genome (6,7). Changes in heterochromatin domains, expression of specific histone isoforms and specific 3D genome reorganization characterize different forms of senescence (3,8).

Typical enhancers (TEs) and super-enhancers (SEs) are important distal regulatory elements that control the transcription of genes (9). SEs are usually defined as large clusters of TEs with a length of 5–50 kb. Epigenetically, they are characterized by the presence of H3K27ac and H3K4me1 marks (10). During senescence, selected TEs and SEs are activated to orchestrate the transcription of the senescence program (9,11,12). HDAC4, a class IIa member of the HDACs family, is degraded by the ubiquitin-proteasome system in response to various forms of senescence. Degradation of HDAC4 contributes to the activation of TEs and SEs during the initial phase of senescence and to senescence fate determination (9).

Senescent cells are also characterized by the presence of irreparable DNA damage leading to permanent cell cycle arrest (13,14). Histone acetylation is not limited to shape transcriptional programs, but also occurs in response to DNA damage to enable loading of the DNA damage response (DDR) machinery (15,16). In principle, age or environment-related changes in specific epigenetic regulators could be responsible for the accumulation of unresolved DNA damage and the concomitant priming of the senescence program (17). Epigenetic regulators involved in both the control of the DDR and the execution of the senescence program may therefore represent a key evolutionary outcome to buffer the negative effects of unresolved DNA damage (18). In this manuscript, we explored the contribution of HDAC4 to the regulation of DDR. We discovered an epigenetic complex that links the accumulation of unrepaired DNA damage to the activation of senescence.

## Materials and methods

### Experimental design

Three models of cellular senescence have been adopted. (i) The knock-out of *HDAC4* in leiomyosarcoma cells re-expressing a Cas9 resistant (PAM mutated) tamoxifen-inducible (ER-fused) HDAC4. (ii) The knock-out of *HDAC4* in melanoma cells (A375) re-expressing a doxycycline-inducible PAM mutated HDAC4. (iii) The inducible expression of HRASG12V in BJ/*hTERT* fibroblasts. *HDAC4^PAM^* (HDAC4 CDS NM_006037) bears point mutation (V31L) in correspondence to the PAM to prevent its targeting by Cas9. Its efficacy, similar to the WT form has been previously validated (9). For each cell line, the effect of HDAC4 KO on senescence was observed in different clones (*n* > 4) and using 4 different sgRNAs (5). Three models of controlled DNA damage and repair have been adopted: the expression of the mega-endonuclease I-PpoI in SK-LMS-1 and BJ/*hTERT* cells; the expression of I-SceI in the reporter cells U2OS/EJ5 (NHEJ) and U2OS/TRI-DR (HR); the recruitment of DDR complexes in laser micro-irradiated cells.

### Cell culture and reagents

BJ/*hTERT*, SK-LMS-1 (TP53*^wt/G245S^*) and SK-UT-1 (TP53*^R175H/R248Q^*) were previously characterized (5). HEK293T, LinXE, Ampho Phoenix and MEFs *HDAC4^loxp/loxp^* cells were cultured as previously described (5) in 10% FBS DMEM (Euroclone). To achieve *Hdac4* knock-out, MEFs *HDAC4^loxp/loxp^* were retrovirally infected to express Cre-ER and were treated with 4OHT. A375 (*TP53^wt/wt^*) melanoma cells were grown in RPMI-1640. SK-LMS-1*^HDAC4-/-/HDAC4PAM-ER^*, U2OS/TRI-DR, U2OS/EJ5 were previously described and characterized (9,19). For the experiments performed in hypoxia, cells were grown in hypoxic chambers at 37°C, 5% CO_2_, 2% O_2_ (Baker Ruskinn). The following chemicals were used: 1 μM 4OHT, 1 μM Doxycycline, 1 μM MG132, 0.5 μM aphidicolin, 3 μM PD0032991, 0.4% Trypan blue, 1 μM PD0332991, 500 nM aphidicolin (all from Sigma-Aldrich), 3.125 μM camptothecin (Enzo Life Sciences), 20 μM etoposide (Enzo Life Sciences), 2.5 μM PCI-34051 (Tocris), 20 μM Scr7 (Tocris), 20 μM Mirin (Tocris), 2.5 μM SAHA (Alexis). Parental BJ and A375 cells were STR profiled and authenticated for this paper (Eurofins, Ebersberg). SK-LMS-1 (HTB-88) and SK-UT-1 (HTB-114) were from ATCC (Virginia, USA). Monoclonal SK-LMS-1 and SK-UT-1 used to generate HDAC4 KO were compared to parental cells by RNA-seq (9,19). All cell lines were routinely tested from Mycoplasma contamination using Hoechst 33342 (Sigma) staining and qPCR.

### Plasmid construction, transfection, retroviral and lentiviral infection, silencing

pLENTI-CRISPR/V2 (Plasmid #52961), pSpCas9(BB)-2A-GFP(PX458) (Plasmid #48138), pSpCas9(BB)-2A-Puro (PX459) (Plasmid #62988), pCW-Cas9 (Plasmid #50661), pX330-BbsI-PITCh (Plasmid #127875), pCRIS-PITChv2-FBL (Plasmid #63672), pCW57/Hygro-MCS1-2A-MCS2 (Plasmid #80922), Apple-TP53BP1trunc (Plasmid #69531), pDEST-mCherry-LacR-BRCA1 (Plasmid #71115), pDEST-FRT/T0-GFP-BRCA1 (Plasmid #71116), pLenti CMV/TO GFP-MDC1 (779–2) (Plasmid # 26285), pCW-GFP-CtIP (Plasmid #71109), pEGFP-N1/H2B-GFP (Plasmid #11680), MSCV-CreERT2-Puro (Plasmid #22776), pLenti-Puro (Plasmid #39481), pBABe-HA-ER-IPpoI (Plasmid #32565), pLew100::NLS-ISceI-HA (Plasmid #21299), SeeSaw Reporter (SSR2.0) (Plasmid # 50393) were obtained from Addgene. pLKO-Puro/shHDAC4 -1 (TRCN0000314667) and -2 (TRCN000004832), pLKO-Puro shHDAC1 (TRCN0000004818) and pLKO-shHDAC2 (TRCN0000004823) were obtained from Sigma-Aldrich. pWZL-Hygro-HDAC4PAM-ER and pCW-Hygro-HDAC4PAM were obtained by sub-cloning a mutagenized HDAC4 (QuikChange Site-Directed Mutagenesis Kit, Agilent) into the linearized empty backbones by a restriction-based approach. pWZL-Hygro-ER acceptor plasmid was previously described (5). Apple-TP53BP1trunc and H2B-GFP were sub-cloned respectively in pBABE-Zeo and pWZL-Neo. pWZL-NeoGFP-LMNB1 and pWZL-Neo/H1.2-GFP were obtained by amplifying the relative cDNA from IMR90 cells. pWZL-Hygro/HRASG12V-ER and pBABE-Puro/HRASG12V were previously described (5). pWZL-Neo/HDAC4-GFP, pWZL-Neo/HDAC4Δ600, pWZL-Neo/HDAC3-GFP, pWZL-Neo/HDAC4-3xFLAG, pCW-Puro/HRASG12V and pWZL-Neo/H2B-GFP were obtained by sucloning. pLKO-Hygro plasmid expressing the same shRNAs were obtained by oligo cloning. H2BK120Q was obtained by site-directed mutagenesis (Quick-Change lightning, Agilent). pCMV6-XL4 HDAC1 was obtained from Origene (Rockville, USA). All the generated plasmids were checked by restriction and sequencing. Transfections, viral infections and siRNA delivery were done as previously described (5). For silencing, cells were seeded in 12-well plate and transfected for 24–48 h with Lipofectamine 3000 (Life Technologies, 3μl per well). The following siRNAs (74 pmol) were used: *HDAC4* (CCACCGGAAUCUGAACCACUGCAUU, Invitrogen Stealth), *HDAC1_A* (CAGCGACUGUUUGAGAACC, Sigma-Aldrich), HDAC1_B (CUAAUGAGCUUCCAUACAA, Sigma-Aldrich), *HDAC2_A* (GCGGAUAGCUUGUGAUGAA, Sigma-Aldrich), *HDAC2_B* (GCAAAGAAAGCUAGAAUUG, Sigma-Aldrich), *HDAC3* (GAUGCUGAACCAUGCACCU, Sigma-Aldrich), scramble control siRNA (UAAGGCUAUGAAGAGAUA, Invitrogen Stealth). *HDAC5*, *HDAC7* and *HDAC9* siRNAs were previously described (5). The following esiRNAs were used to minimize off-targets: *HDAC4* (EHU136981), *RAD51* (EHU045521), *HDAC3* (EHU035581), *HDAC5* (EHU123121), Control (EHURLUC) (Sigma-Aldrich).

### CRISPR/Cas9 Genome editing and Precise Integration into Target Chromosome (PITCh)

SpCas9 was stably transduced to generate SK-LMS1*^HDAC4-/−^* clones, while SpCas9 was transiently transfected (Lipofectamine 2000, LifeTechnologies) to generate A375*^HDAC4-/−^* clones (5). The Cas9 resistant HDAC4 (*HDAC4^PAM^*) was continuously re-expressed in a 4OHT dependent (SK-LMS-1 clone 66) or DOX dependent (A375 KO clone) manner (5). For the targeting of endogenous *HDAC4* with *EGFP*, PITCh knock-in method was used (20). Briefly, SK-LMS-1 cells were transfected with 4 μg pX330-BbsI-PITCh-sgRNA HDAC4 3′ (GCTTCGAGGGAGTGCTACAG) and 12 μg pCRIS-PITChv2-HDAC4 GFP by using 30 μl TransIT-CRISPR Transfection Reagent (Mirus Bio). Monoclonal cultures were obtained by using cloning cylinders (Sigma-Aldrich); GFP + clones, identified through Enspire spectrofluorometer plate reader (PerkinElmer), were expanded and subjected to immunoblotting and validated by Sanger sequencing. pCRIS-PITChv2/HDAC4-GFP was obtained from pCRIS-PITChv2 FBL by replacing the micro-homology region of FBL with the one of *HDAC4* (CCGCGTTACATAGCATCGTACGCGTACGTGTTTGGTGGAAGAGGAGCCGCCCCTGCCGGATCCATGGTGAGCAAGGG) by Seamless Ligation Cloning (21).

### Immunofluorescence and immunoblotting

Immunofluorescence was performed as previously described (22). Briefly, cells were fixed with 3% paraformaldehyde and permeabilized with 0.3% Triton X-100. The secondary antibodies were Alexa Fluor 488-, 532- 546- or 633-conjugated anti-mouse and anti-rabbit secondary antibodies (Molecular Probes). For S phase analysis, cells were grown for 3 hours with 50 μM Bromodeoxyuridine (BrdU, Sigma-Aldrich). After fixation, coverslips were treated with HCl (1% and 2%), quenched with borate buffer, and processed for immunofluorescence. TP53BP1 (#4937, Thermo Fisher Scientific), CtIP (F-2, sc-28324 Santa Cruz Biotechnology), LMNB1 (ab16048, Abcam), LMNA (E-1, sc-376248 Santa Cruz Biotechnology) antibodies were used in immunofluorescence. Cells were imaged with a confocal microscope Leica AOBS SP8 or with Leica AF6000 LX. Nuclei were stained with Hoechst 33342 (10 μg/ml). Unless specifically explained, images represent maximum intensity projections of 3D image stacks and were adjusted for brightness and contrast for optimal visualization. Cell lysates after SDS-PAGE and immunoblotting on nitrocellulose (Whatman) were incubated with primary antibodies. HPR-conjugated secondary antibodies were obtained from Cell Signalling and blots were developed with Super Signal West Dura (Thermo Fisher Scientific). Odyssey Infrared Imaging systems (LI-COR Biosciences) was used to detect the fluorescence signal of secondary fluorescent antibodies (anti-Rabbit CF770 SAB4600215, anti-Mouse CF680 SAB4600361 Merck). For stripping, primary and secondary antibodies were removed by using Restore PLUS Western Blot Stripping Buffer (Thermo Fisher Scientific), according to manufacturer. Unless otherwise indicated, all the immunoblot figures were representative of at least two biological replicates. The primary antibodies used in this work are listed in Supplementary Table S7.

### Time-lapse video microscopy and laser micro-irradiation

SK-LMS-1 and SK-LMS-1*^HDAC4-/-/HDAC4PAM-ER^*cells engineered to express H2B-GFP and Apple-TP53BP1 trunc or H2B-GFP alone were seeded on fibronectin coated 35 mm glass bottom dishes (MatTek) at low density (0.3 × 10^5^ cells). After 24 h, medium was refreshed and 4OHT was added in HDAC4 re-expressing cells. Six hours later, the dishes were housed in a stage-top live cell imaging chamber (Okolab) on a Leica TCS SP8 X confocal microscope, maintained in a humidified atmosphere at 37°C and 5% CO_2_ and imaged every 10 min. for 74 h under four dimensions using a 100×/1.40 NA oil immersion objective. 5-step z-stacks were collected for each time-point. Laser power, total dwell time, pinhole aperture and acquisition intervals were chosen appropriately to minimize toxicity and bleaching. For laser micro-irradiation, 0.6 × 10^5^ SK-LMS-1 cells were seeded on fibronectin coated 35 mm glass bottom dishes (MatTek) and transfected with plasmid encoding TP53BP1, MDC1, CtIP, BRCA1. After 8 h, medium was changed, and cells were silenced or not with HDAC4 esiRNA (74 pmoles, Sigma-Aldrich). 16 hours later, cells were treated with BrdU (100 μM) for the following 16 h for the pre-sensitization step. Experiments were performed on a Leica TCS SP8 X confocal microscope equipped with a stage-top environmental chamber and a 63×/1.40 NA oil immersion objective. Cells were irradiated using a 405 nm diode laser. The following settings were used: 100% laser output, zoom factor 1.25, 16 × 16 scan format, 10 Hz, bidirectional scan, 100 scanning cycles. Images were acquired at 2 AU pinhole size every 10 seconds for 30 min. Brightness and contrast adjustments have been made to the entire image and consistently applied to every frame in the same experiment.

### Immunoprecipitation

A hypotonic buffer was used for cell lysis (20 mM Tris–HCl pH 7.4, 10 mM KCl, 10 mM MgCl_2_, 1% Triton X-100, 10% glycerol, 1 mM phenylmethylsulphonylfluoride, 5 mM NaF, 1 mM Na_3_VO_4_), supplemented with protease inhibitors. Lysates were incubated O/N with 1 μg anti-HDAC4 (5), rabbit IgG, anti-FLAG M2 (Sigma-Aldrich), mouse IgG, and for 2 h with 8 μl Protein A Dynabeads (Thermo-Fisher Scientific). To avoid interfering IgG chains, when required primary antibodies were crosslinked to beads as explained before (19). After four washes with lysis buffer, the immunocomplexes were reversed with 2× Laemmli sample buffer, boiled, resolved by SDS-PAGE and subjected to western-blotting. 1/100 of total lysate has been collected as input.

### Mass spectrometry-based immuno-precipitation proteomics

10 × 10^6^ SK-LMS-1*^HDAC4/GFP-PITCH^* cells were harvested at the exponential phase of growth and lysed with 2 ml of the hypotonic buffer described above. 800 μg lysates were incubated O/N with 16 μl ChromoTek GFP-Trap Magnetic Agarose beads (Proteintech, Rosenmont, USA) or ChromoTek V5-Trap Magnetic Agarose beads, as a control. After four washes with lysis buffer, the immunocomplexes were reversed with 2× Laemmli sample buffer and resolved by SDS-PAGE. After in gel trypsin digestion, peptides were resuspended in 20 μl of 0.1% formic acid and then subjected to Nano UPLC (Ultimate 3000 nano UHPLC system, ThermoFisher Scientific, USA) MS/MS analysis. The full scan was performed between 300 and 1650 *m*/*z* at the resolution 60 000 at 200 *m*/*z*, the automatic gain control target for the full scan was set to 3e6. The MS/MS scan was operated in Top 20 mode using the following settings: resolution 15 000 at 200 *m*/*z*; automatic gain control target 1e5; maximum injection time 19ms; normalized collision energy at 28%; isolation window of 1.4 Th; charge sate exclusion: unassigned, 1, >6; dynamic exclusion 30 s. Raw MS files were analyzed and searched against *Homo sapiens* protein database based on the species of the samples using Maxquant (1.6.2.6). The parameters were set as follows: the protein modifications were carbamidomethylation (C) (fixed), oxidation (M) (variable); the enzyme specificity was set to trypsin; the maximum missed cleavages were set to 2; the precursor ion mass tolerance was set to 10 ppm, and MS/MS tolerance was 0.5 Da. MS/MS analysis was performed by Creative Proteomics (Shirley, USA).

### dCas9-targeted locus-specific protein isolation (CLASP)

1.0 × 10^7^ SK-LMS-1/HA-ER-I-PpoI/dCas9 cells were transfected with 740 pmoles esiRNAs and 100 μl Lipofectamine 2000 (Thermo Fisher) and dCas9 expression was induced with Doxycycline (1 μg/ml). 36 h later cells were treated with 4OHT (5 μM) and after 3 h were transfected with 5 μg each of three *in vitro* transcribed sgRNAs (sgRNA1: CTACCTTAAGAGAGGTCCAA; sgRNA2: AAAGGTGCTCAGCTCTGAAA; sgRNA3: ATGAATGAATAAACCATACG) designed around I-PpoI cleavage site on DAB1 locus and 90 μl of TransIT-CRISPR Transfection Reagent (Mirus Bio). Precision gRNA Synthesis and Purification Kit (Thermo Fisher) were used to prepare sgRNAs. sgRNAs were also dephosphorylated as previously reported (5). Three hours after the transfection, cells were crosslinked with 1% formaldehyde for 10 min., chromatin was extracted with SDS Lysis Buffer (1% SDS, 10 mM EDTA, 50 mM Tris–HCl pH 8.0), sonicated (30 cycles, Bioruptor, Diagenode) and immunoprecipitated with 5 μg anti-Cas9 antibody (8C1-F10, Active Motif) and 15 μl Protein G Dynabeads (Thermo Fisher). After the washing steps, beads were resuspended in 200 μl NT2 buffer (50 mM Tris–HCl pH 7.5, 150 mM NaCl, 1 mM MgCl_2_, 0.05% NP-40, 5u DnaseI) and incubated 30 min. at 37°C. Crosslinking was reversed by resuspending magnetic beads with 15 μl Re-ChIP elution buffer (TE, 2% SDS, 15 mM DTT, PIC) and 5 μl 4× Laemmli Sample buffer and incubated at 90°C for 5 min. Eluted proteins were subjected to SDS-PAGE.

### Glutathione S-transferase (GST) pull-down

2 μg GST, GST-MEF2D (1–190) (22) and GST-HDAC2 (SRP5266, Merck) were incubated with a lysate obtained from SK-LMS-1 cells by using an hypertonic lysis buffer containing 300 mM NaCl in order to destroy the complexes as much as possible. Pulldown was conducted at 4°C with rotation for 2 h, by using GST-Sepharose 4B beads (Cytiva, Marlborough USA). Laemmli 2× sample buffer was used to elute baits and prays from beads.

### Production and purification of recombinant GST-H2B-GFP

H2B-GFP was subcloned from pBABE-PURO by inserting H2B-GFP fusion restricted EcoRI-SalI into pGEX4T1 plasmid linearized with EcoRI-XhoI. GST-tagged H2B-GFP was expressed in Escherichia coli BL21-DE3. Bacteria were grown at 37°C and 230 rpm to an OD of 0.6 and IPTG (1 mM) was used to induce the protein production (2 h at 30°C). Bacteria pellet was lysed with GST lysis buffer (PBS pH 7.4, 1% Triton X-100; 0.2% SDS, 0.5% NP40, 0.1% Tween-20, Protease inhibitors, 1ml GST lysis buffer for 25 ml of bacteria liquid culture). After 30 min. of lysis in ice, the lysate was sonicated (SonoPlus loop sonicator, 5 heats 30 s). Soluble fraction was obtained after centrifugation (3500 rcf, 10 min.) and loaded on Biorad affinity chromatography columns prepacked with Glutathione Sepharose 4 Fast Flow (GE Healthcare). Elution by competition was adopted (50 mM Tris–HCl pH 8.0, 10 mM GSH), followed by dialysis in 50 mM Tris–HCl pH 7.5, 150 mM NaCl. For *in vitro* acetylation/ubiquitylation assay GST-H2B-GFP was rebound to the matrix.

### *In vitro* H2B acetylation and ubiquitylation reactions

Cells were used as sources of enzymes. 300 μg of total lysate was used for each assay starting from at least 1 × 10^6^ SK-UT-1 cells and 0.5 × 10^6^ MEFs. Cells were lysed in Lysis Buffer (200 mM Tris–HCl pH 7.5, 10 mM KCl, 10 mM MgCl_2_, 1% Triton X-100, 1% glycerol, 1 mM PMSF, 5 mM NaF, 1 mM Na_3_VO_4_) for 10 min. rocking at 4°C. Next, the solution was made 150 mM NaCl. For each reaction, 1 μg of recombinant GST-H2B-GFP (20 ng/μl) were bound to 40 μl of GST-Sepharose resin and incubated with 750 μl lysate, 240 μl 10 mM ATP (2 mM final), 24 μl DTT 1M (2 mM final), 12 μl PIC for 60 min. at 30°C. Beads were washed trice with 1 ml Lysis Buffer and H2B was eluted by incubating beads at 75°C with 20 μl Laemmli-sample buffer for 5 min. For immunodepletion experiments, lysates generated as explained above were incubated for 6 h with 3 μg anti-HDAC4 antibody and 20 μl Protein A Dynabeads. The supernatant was taken as immunodepleted fraction.

### *In vitro* H2B deacetylation

293 cells were transfected with 15 μg plasmid encoding H2B-GFP or H2B K120C-GFP by using 30μl polyethylenimine (PEI, 1 μg/ml). 72 h later, cells were lysed with 1ml RIPA buffer (10mM Tris–HCl pH 7.5, 150 mM NaCl, 1 mM EDTA, 1% Triton X-100, 0.1% SDS, 0.1% Sodium deoxycholate, 1 mM PMSF, 5 mM NaF, 1 mM Na_3_VO_4_). H2B-GFP was immunoprecipitated for 4 h with 4.5 μg anti-GFP antibody and 8 μl Protein A Dynabeads. Beads were washed twice with 1 ml RIPA buffer and once with HDAC buffer (25 mM Tris–HCl pH 8, 137 mM NaCl, 2.7 mM KCl, 1 mM MgCl_2_) and resuspended in 300 μl HDAC buffer. This suspension was divided in three reactions of 100 μl each containing respectively 100 ng recombinant GST (22), GST-HDAC2 (Merck), GST-HDAC2, 2.5 μM SAHA and incubated for 30 min. at 30°C. H2B-GFP was collected by placing tubes in a magnetic stand, eluted with 20 μl Laemmli sample buffer and subjected to SDS-PAGE.

### DR-GFP and EJ5-GFP reporter system

2.5 × 10^6^ TRI-DR-GFP and EJ5-GFP U2OS cells were transfected with 148 pmol of control or HDAC4 siRNA. After 18 h each plate was divided in two and transfected or not after 12 h with 3 μg of I-SceI. 72 h later the HR and NHEJ efficiency was determined by quantifying GFP-positive cells by flow cytometry. PCR was used to further monitor HR. gDNA was extracted with the Quick-DNA Miniprep Kit (Zymo Research) and PCR was performed with KAPA HiFi HotStart ReadyMix by using primers P1 (GAGGGCGAGGGCGATGCC), P2 (TGCACGCTGCCGTCCTCG), F1 (TTTGGCAAAGAATTCAGATCC), F2 (CAAATGTGGTATGGCTGATTATG), as previously described (23). *ACTB* amplicon was used to normalize the amplicon intensity. The following PCR protocol was used: 98°C/5 min. × 1 cycle; 98°C/45 s., 70°C/30 s., and 72°C/30 s. × 25 cycles; 72°C/10 min. × 1 cycle. 8 μl of the PCR products were loaded on a 1% agarose gel and visualized by EtBr staining.

### Flow cytometry

For GFP-positivity evaluation, single-cell suspensions were prepared as previously described (24) and acquired on BD FACSCalibur cytometer. Data were analyzed with FlowJO software.

### SA-β-gal assay

SA-β-gal assay was performed as previously described (5). After fixation cells were incubated for 16 h with staining solution: 40 mM citric acid/Na phosphate buffer, 5 mM K_4_[Fe(CN)_6_]3H_2_O, 5 mM K_3_[Fe(CN)_6_], 150 mM NaCl, 2 mM MgCl_2_ and 1 mg/ml X-gal (Panreact Applichem). Images were acquired with Leica LD bright field optical microscope.

### RNA extraction and quantitative qRT-PCR

Cells were lysed using Tri Reagent (Molecular Research Center). 1.0 μg of total RNA was DNAseI treated (NEB #T2010) and reverse-transcribed by using 100 units of M-MLV Reverse transcriptase (Life Technologies) in the presence of 1.6 μM oligo(dT) (Sigma-Aldrich) and 4 μM Random hexamers (Euroclone). qRT-PCRs were performed using SYBR green technology (KAPA Biosystems). qPCR of gDNA was performed by using 2.5 ng gDNA as template. Data were analyzed by comparative threshold cycle (delta delta Ct) using *HPRT, GAPDH* as normalizers for qRT-PCR, or *ACTB* and *GAPDH* as qPCR normalizers.

### DNA end resection assay

To quantify 5′ strand resection at DNA double strand breaks a strategy previously established (25) was adopted and adapted to I-PpoI cleavage. Briefly, gDNA was extracted and restricted for 6 h with 20 units of BamHI and HindIII (New England Biolabs). 2.5 ng of purified DNA were then subjected to qPCR by using primer pairs straddling the endonuclease cleavage sites.

### ChIP, library construction, ChIP-seq and NGS data analysis

Chromatin was obtained from BJ-*hTERT*, BJ-*hTERT/RAS-ER* and BJ-*hTERT/RAS-ER/HDAC4* cells and immunoprecipitated with 3 μg of anti-H2BK120ac, 5 μg of anti-γH2AX and 4 μg of anti-HDAC4 antibodies or control IgG, as previously described (9). The specificity of HDAC4 antibody was evaluated on HDAC4 KO samples (9). 5 ng of total DNA were used to prepare ChIP-seq libraries. Two sequencing experiments from independent ChIP-seq experiments, each pooling at least two distinct biological replicates, were performed and compared. A Pearson's correlation coefficient (PCC) test on the two sequencing experiments assessed the reproducibility of each ChIP-seq (26). The FastQC/MultiQC programs were used to evaluate the quality of sequencing reads and Bowtie 2 was used to align them to NCBI GRCh38 human genome reference. Peak calling was performed against input sequences using EDD for γH2AX ChIP (–write-log-ratios and –write-bin-scores options) and MACS2 for HDAC4 and H2BK120ac (–nomodel, –extsize 150 and -q 0.05 options); IDR peaks identified by comparing the replicates were considered for analysis, as previously described (9). Gene annotations were performed as previously described (9). gplots, biomaRt and Gviz R/Bioconductor packages and the deepTools suite were used to generate peak heatmaps and profiles. The bedtools toolset was used to identify overlaps of at least one nucleotide as previously described (5). H3K27ac and H3K4me1 peak calling was performed against input sequences using MACS2 (‘sharp’ mode). Enhancers were defined accordingly to the co-presence of H3K27ac and H3K4me1 enriched signals and excluding H3K27ac peaks that were located within ±2 kb region of any RefSeq annotated promoter. ROSE algorithm (27) was used to rank these identified enhancers in respect to H3K27ac ChIP-seq signal, accordingly to (28), thus defining SEs. Known and novel motif discovery was performed using the MEME-ChIP tool from the MEME Suite, as previously described (9). Using a 1-order background model to normalize for biased distribution of letters and groups of letters in the analyzed sequences allows to specifically adjust the background for dimer biases. The identified enriched motifs were compared to JASPAR2018_CORE_vertebrates_non_redundant and uniprobe_mouse databases for annotation using Tomtom Motif comparison Tool.

The R Bioconductor package DiffBind was used to perform differential binding analysis of H2BK120ac ChIP-seq peak data. FDR <0.05 was used as a cut-off for differential binding affinity analysis in Figure 7. Ease-seq (29) was used to calculate H2BK120ac rates between the indicated samples and plot read density and intensity in the heatmaps in Figures 7 and 8. UCSC Wiggle (Wig) Track Files v1.5.4 were created for Supplementary Figure S1A (smoothing window: 2) after peak signals conversion in normalized read counts (1 Mb window) (30).

### Statistical analysis

For experimental data, Student's *t*-test was employed. Mann–Whitney test was applied when normality could not be assumed. *P*< 0.05 was chosen as statistical limit of significance. For comparisons between more than two samples, the Anova test was applied coupled to Kruskal–Wallis and Dunn's Multiple Comparison Test. For correlation between two variables, Pearson correlation or Spearman correlation were calculated for normal or non-normal distributions, respectively. Excel and GraphPad Prism were used for routineer analysis, R/Bioconductor packages for large data analysis and heatmap generation. We marked with *P< 0.05, **P< 0.01, ***P< 0.001. Unless otherwise indicated, all the data in the figures were represented as arithmetic means ± the standard deviations from at least three independent experiments.

### Critical reagents

A list of oligonucleotides and antibodies is provided in Supplementary Table S7.

## Results

### The accumulation of DNA lesions is an early event during senescence triggered by HDAC4 depletion

**Figure 1.**
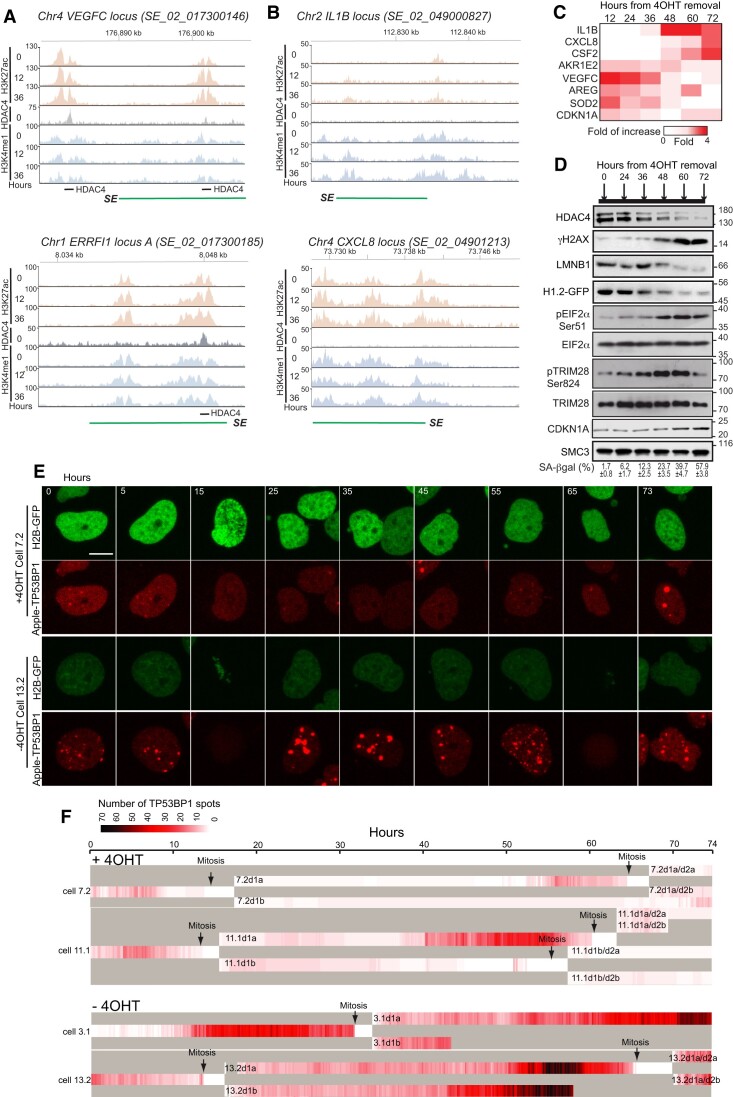
The accumulation of DNA lesions is an early event during senescence induced by HDAC4 depletion. (**A**) Representative loci of SEs activated by HDAC4 depletion in SK-LMS-1*^HDAC4-/-/HDAC4PAM-ER^* cells (at the indicated time point, expressed in hours, after HDAC4 depletion) and characterized by the binding of HDAC4 at time point 0. Shown are the intensities of H3K27ac, H3K4me1 and HDAC4 peaks. These two SEs were registered in SEdb 2.0 (92) with the ID shown in the picture. (**B**) Representative loci of SEs activated by HDAC4 depletion in SK-LMS-1*^HDAC4-/-/HDAC4PAM-ER^* cells (at the indicated time point, expressed in hours, after HDAC4 depletion) and characterized by the binding of HDAC4 at time point 0. Shown are the intensities of H3K27ac, H3K4me1 and HDAC4 peaks. Both these SEs were registered in SEdb 2.0 (92) with the ID shown in the picture. (**C**) Heat map showing fold induction of SES-associated genes at the indicated time point after HDAC4 depletion in SK-LMS-1 cells as determined by qRT-PCR. Data refer to the previous time point and for the 12-h time point to time point 0. Mean ± SD; *n* = 3. (**D**) Total cellular lysates were generated from SK-LMS-1 cells undergoing premature senescence harvested at the indicated time after HDAC4 depletion. Immunoblot analysis was performed using the indicated antibodies, SA-βgal positivity is indicated. Mean ± SD; *n* = 3. (**E**) Representative frames of time-lapse experiments with SK-LMS-1*^HDAC4-/-/HDAC4PAM-ER^* cells expressing H2B-GFP and Apple-TP53BP1 reporters, grown in the presence or absence of 4OHT to abrogate HDAC4 expression. Numbers indicate hours. Bar 10 μm. (**F**) Heatmap representing the quantification of the TP53BP1 foci/bodies in SK-LMS-1*^HDAC4-/-/HDAC4PAM-ER^*, during 74 h of analysis starting from 6 h after 4OHT removal (time ‘0’), as indicated. Typical cells for the + 4OHT and the -4OHT conditions (respectively cell n.10 and n.9) are represented. The signal intensity is proportional to the number of TP53BP1 spots. The beginning of the mitosis is indicated by arrows. The daughter cells arising from each mitosis are indicated and labelled as ‘d’.

We initially selected leiomyosarcoma SK-LMS-1 cells, deleted for HDAC4 using CRISPR/Cas9 and re-expressing a 4-hydroxytamoxifen (4OHT)-inducible PAM mutant version of HDAC4 (*HDAC4^−/−^/HDAC4^PAM-ER^*) as a model of inducible senescence (9). In this model, that we defined HDAC4-dependent senescence (HDS), removing 4OHT from the culture medium triggers senescence (Supplementary Figure S1A). We previously defined SEs activation after 36 h of HDAC4 deletion (27). Here, to determine the timing of SEs activation, ChIP-seq for H3K27ac and H3K4me1 was also performed 12 h after 4OHT removal. The ROSE algorithm (27) was used to define these SEs. The deletion of *HDAC4* leads to rapid reorganization of chromatin with the formation of new SEs that double within 12 h from 4OHT removal (from 647 at time 0, to 1102 and 1451 respectively at 12 and 36 h) (Supplementary Figure S1B and Supplementary Table S1). The new SEs formed during the induction of senescence are termed super-enhancers of senescence (SES). In these SES, H3K27ac levels are already increased 12 h after HDAC4 depletion (Figure 1A and Supplementary Figure S1B, C).

SESs discovered to be directly bound to HDAC4 by ChIP-seq, were classified as SESs directly regulated by HDAC4 (5) (Figure 1A and Supplementary Figure S1C). The effect of HDAC4 depletion on these SES is coupled to the early induction of the corresponding genes *AKR1E2, VEGFC, SOD2* and *AREG* (Figure 1C) (9). Other SES that do not evidence the binding of HDAC4, as in the case of several SASP genes (Figure 1B and Supplementary Figure S1C), where defined SES under the indirect influence of HDAC4. Induction of SASP genes (*IL1B, CXCL8, CSF2*) occurs later (Figure 1C, and Supplementary Figure S2A). Finally, upregulation of *CDKN1A* occurs early and its SES is not directly bound by HDAC4. The differential upregulation of these SES-associated genes was confirmed when senescence was induced by depletion of HDAC4 in melanoma cells (Supplementary Figure S2B).

The appearance of senescence is also characterized by additional changes such as: (a) the activation of a chronic DDR; (b) the engagement of cyclin-dependent kinase inhibitors; (c) the upregulation of anti-apoptotic genes; (d) the occurrence of endoplasmic reticulum (ER) stress; (e) the disassembly of nuclear lamina (31). The timing of the appearance of: (i) DNA damage (γH2AX and TRIM28 phosphorylation), (ii) Lamin B (LMNB1) dismantling, (iii) chromatin changes (H1-GFP signal) and (iv) ER-stress (eIF2a phosphorylation) (14) was compared with the upregulation of genes under SES control (Figure 1C/D, Supplementary Figure S2C/D). These senescence markers become clearly visible only 48 h after HDAC4 depletion (Figure 1D, and Supplementary Figure S2D). The presence of multilobed nuclei and the accumulation of cytosolic chromatin fragments (CCFs), two other senescence markers (32), was also recorded (Supplementary Figure S2C/D). At 24 h after HDAC4 depletion, cells begin to accumulate DNA damage and nuclear alterations (appearance of lobed nuclei and CCFs).

The accumulation of DNA damage was confirmed in the second model of HDS, A375 melanoma cells silenced for HDAC4 (Supplementary Figure S2E) or knocked-out by CRISPR/Cas9 (Supplementary Figure S2F). In A375 cells removal of HDAC4 triggered TP53 accumulation. DNA damage increased linearly in the first 48 h after HDAC4 removal and even more in the next 24 h (Supplementary Figure S2F). To summarize, HDAC4 depletion contributes to activate SES and triggers DNA damage, as early responses. Consistent with previous studies, loss of LMNB1 is a secondary event that foregoes SASP (33).

### Single cell analysis after HDAC4 depletion highlights the pre-mitotic accumulation of DNA damage

Unperturbed cells accumulate DNA lesions during interphase, possibly during progression through S phase (34). To timely monitor the occurrence of DNA lesions during HDS *in vivo*, we engineered SK-LMS-1*^HDAC4-/-HDAC4PAM-ER^*cells to express the H2B-GFP and Apple-TP53BP1 reporters. H2B is a senescence-resistant nuclear marker while TP53BP1 is a mediator of DNA double-strand breaks (DSBs) repair (35). The TP53BP1 probe was validated by comparing its activation pattern to endogenous TP53BP1. Both endogenous TP53BP1 and Apple-TP53BP1 form small foci and larger dots (Supplementary Figure S2G-I). Previous studies have defined these large dots as TP53BP1 nuclear bodies (TP53BP1-NBs), which are associated to under-replicated DNA (35,36). Etoposide treatment was used as a positive control (Supplementary Figure S2G). Subsequently, cells were observed by time-lapse confocal microscopy *in vivo* over a period of 74 h. The distribution of TP53BP1 dots evidences the accumulation of DSBs (Figure 1E and Supplementary Video S1), reflecting the formation of spontaneous DNA lesions (36). After removal of HDAC4, cells accumulated several TP53BP1 nuclear foci and NBs (Figure 1E and Supplementary Video S2).

The accumulation of TP53BP1 dots and TP53BP1-NBs was quantified from different time-lapse experiments and shown in a heat map (Figure 1F, and Supplementary Figures S2H/S2I/S3/S4). During the period of analysis, the cell cycle length was constant (43.22 + 3.40 h) and 9 of 10 cells expressing HDAC4 entered mitosis twice (Figure 1FSupplementary Figure S3/S4 and Supplementary Video S1). Removal of HDAC4 (-4OHT) impaired cell proliferation, as *HDAC4^−/−^* cells frequently failed to enter the second mitosis (except for cells 13.2 and 19.1) and accumulated TP53BP1 nuclear foci and NBs (Figure 1E/F, Supplementary Figure S3/S4, and Supplementary Video S2). These TP53BP1 NBs were not symmetrically distributed among sister cells after mitosis (Supplementary Video S2). In conclusion, the absence of HDAC4 exacerbated the trend of DNA lesions accumulation during S and G2 phases.

To correlate the accumulation of pre-mitotic TP53BP1 nuclear dots with mitotic defects, we measured the average number of all TP53BP1 dots for 100 min. before the occurrence of chromosome condensation (onset of mitosis). In the presence of HDAC4, the number of TP53BP1 dots was comparable in the cells examined (between 0 and 3/cell), and mitosis ended after 100–150 min. (Supplementary Figure S5A). Similar behaviour was also observed during the second mitosis. In *HDAC4^−/-^* cells, the number of TP53BP1 nuclear dots in G2 was increased, as was the length of mitosis (Supplementary Figure S5B, C).

As a consequence of unresolved chromosomal lesions transmitted to daughter cells, TP53BP1-NBs re-emerged in G1 (36). In the presence of HDAC4, daughter cells had few symmetrical TP53BP1-NBs and this behaviour was maintained in subsequent mitosis (Supplementary Figures S4 and S5B). In the absence of HDAC4 the number of TP53BP1-NBs was increased, and TP53BP1-NBs were asymmetrically distributed between daughter cells (Supplementary Figures S5A and B and Supplementary Movie S2). The accumulation of macroscopic nuclear alterations such as CCFs, ultrafine DNA bridges (UFBs), lobate nuclei and non-productive mitosis (Supplementary Figure S5D) could explain the mitotic delay observed in the absence of HDAC4.

### HDAC4 depletion triggers replication stress (RS)

DNA damage during senescence can originate from various sources, e.g. changes in the nuclear lamina and atmospheric oxygen tensions (20,21). Importantly, senescence can be prevented by growing cells under low-oxygen conditions (37,38). To elucidate the contribution of oxygen and loss of nuclear lamina to DNA lesion accumulation, we engineered SK-LMS-1*^HDAC4-/-/HDAC4PAM-ER^*cells to express LMNB1-GFP and grew them under normoxia or hypoxia (2% O_2_). Although LMNB1 partially reduced the occurrence of senescence (Supplementary Figure S6A), we can exclude a prominent role of LMNB1 and oxygen in the accumulation of DNA damage after HDAC4 depletion (Supplementary Figure S6B–E). LMNB1 partially prevented the upregulation of SASP genes (*CXCL8, IL1B*) but had no effect on *CDKN1A* expression (Supplementary Figure S6F, G). Importantly, HDAC4-regulated SES-associated genes (*AKR1E2, SOD2, VEGFC, ERRFI1*) were not affected by LMNB1 re-expression (Supplementary Figure S6H).

**Figure 2.**
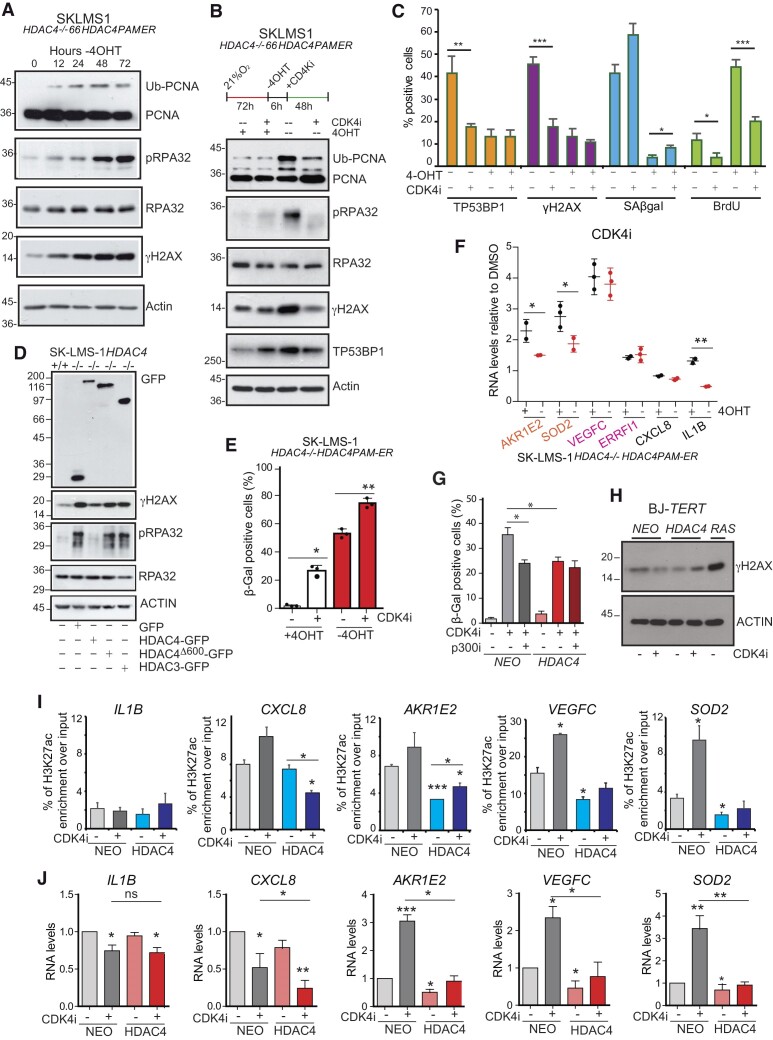
HDAC4 depletion triggers replication stress. (**A**) Immunoblot analysis using cellular lysates from SK-LMS-1*^HDAC4-/-HDAC4PAM-ER^* cells harvested at the indicated time after HDAC4 depletion. The detected proteins are indicated. (**B**) Immunoblot analysis using cellular lysates from SK-LMS-1*^HDAC4-/-HDAC4PAM-ER^*harvested 72 h after the removal or not of 4OHT and the treatment for 48 h with palbociclib/CDK4i, (3μM), as indicated. The detected proteins are indicated. (**C**) Quantification of TP53BP1, γH2AX foci, SA-β-gal and BrdU positivity in SK-LMS-1*^HDAC4-/-/HDAC4PAM-ER^*after removal or not of 4OHT and the treatment for 48 h with palbociclib, as indicated. Mean ± SD; *n* = 3. *P< 0.05, **P< 0.01 and ***P< 0.001. (**D**) Immunoblot analysis in SK-LMS-1 cells wt or KO for HDAC4 and re-expressing HDAC3-GFP or HDAC4 and HDAC4Δ600-GFP, as indicated. The detected proteins are indicated. (**E**) Analysis of senescence after SA-β-gal staining in SK-LMS-1*^HDAC4-/-HDAC4PAM-ER^*cells, treated as indicated. Mean ± SD; *n* = 3. *P< 0.05, **P< 0.01 and ***P< 0.001. (**F**) mRNA expression levels of the indicated genes in SK-LMS-1*^HDAC4-/-/HDAC4PAM-ER^*re-expressing (+4OHT) or not (−4OHT) HDAC4-ER for 24 h prior to the treatment for 48 h with palbociclib. For the two groups of KO cells re-expressing or not HDAC4, fold induction is relative to the matched DMSO-treated cells. The significance is relative to the comparison between the two groups re-expressing or not HDAC4 and treated with the same drug. (**G**) Analysis of senescence after SA-β-gal staining, in BJ-*hTERT* cells expressing the indicated transgenes. Mean ± SD; n = 3. *P< 0.05, **P< 0.01 and ***P< 0.001. (**H**) Immunoblot analysis in BJ/*hTERT* cells expressing HDAC4, RAS or NEO resistance, as a control, and treated as indicated for 72 h with palbociclib. **I**. ChIP-qPCR signals for H3K27ac at the indicated SES in BJ-*hTERT* cells expressing HDAC4 or NEO resistance and treated or not with palbociclib for 72 h. Significance is relative to untreated BJ-*hTERT/NEO* cells. The regions of SES analyzed by ChIP-qPCR were indicated previously (9). Mean ± SD; *n* = 2. *P< 0.05. (**J**) mRNA expression levels of the indicated genes in BJ/*hTERT* cells expressing HDAC4 or NEO resistance and treated or not with palbociclib for 72 h. Fold induction is relative to BJ/*hTERT*/*NEO* DMSO-treated cells. Mean ± SD; *n* = 3. *P< 0.05, **P< 0.01 and ***P< 0.001.

DNA lesions can often arise during DNA replication. Several intracellular and extracellular conditions can trigger RS and forks stalling or collapsing leading to DSBs (39). ATR-mediated hyperphosphorylation of single-stranded (ss)DNA-binding protein RPA32 and monoubiquitylation of PCNA mark damaged replication forks. These post-translation modifications (PTMs) are critical for the recruitment of repair factors and Polη (40). At 12 h after the switch-off of *HDAC4PAM-ER* expression, cells accumulated Ub-PCNA and phosphorylated (S4/S8) RPA32, which became more evident 48 h later. These PTMs were accompanied by the induction of DSBs (Figure 2A). Most of this DNA damage was attributable to RS. Inhibition of cell cycle progression, achieved by the CDK4 inhibitor palbociclib (CDK4i), nearly abolished RPA32 phosphorylation and decreased PCNA ubiquitylation and the increase in DSBs (Figure 2B, C). Accordingly, depletion of HDAC4 showed additive effects to aphidicolin (APH) or camptothecin (CPT) for DSBs accumulation (Supplementary Figure S7A-C). Recovery after the APH block was also delayed by the absence of HDAC4 (Supplementary Figure S7B).

We confirmed the induction of RS after deletion of HDAC4 in A375 and in BJ-*hTERT/RAS/E1A* cells (Supplementary Figure S7D). These cell lines are used as models to study senescence and all these cells enter senescence after HDAC4 silencing (9,41).

HDAC4 binds to the NCOR1/NCOR2/HDAC3 complex via the carboxy-terminal deacetylase domain (42). To gain insight into the mechanism by which HDAC4 buffers RS-induced DNA damage, we re-expressed HDAC4Δ600-GFP or HDAC3-GFP in SK-LMS-1*^HDAC4-/-/HDAC4PAM-ER^* cells. Only re-expression of full-length HDAC4-GFP resulted in almost complete restoration of γH2AX and RPA32 phosphorylation (Figure 2D).

As shown in figure 2C, palbociclib/CDK4i can elicit senescence (43) in the absence of DNA damage (41). Hence, we used palbociclib to investigate whether the effect of HDAC4 on the expression of genes under the control of SES could be separated from the effect on RS. Moreover, palbociclib treatment could clarify whether HDAC4 can suppress senescence in the absence of DNA damage. Palbociclib induces senescence and simultaneous removal of HDAC4 has an additive effect (Figure 2E). Transcription of genes associated with SES that are repressed by HDAC4 is upregulated by palbociclib, and removal of HDAC4 does not further affect their expression (Figure 2F). In contrast, SASP genes (*IL1B, CXCL8*) that are not induced during palbociclib-induced senescence (44), are not induced even in the absence of HDAC4 (Figure 2F).

To confirm the role of HDAC4 in palbociclib-induced senescence, we expressed HDAC4 in human fibroblasts immortalized with hTERT (BJ-*hTERT*). Ectopically expressed HDAC4 reduces CDK4i-induced senescence in BJ-*hTERT* cells, like p300 inhibitor (p300i/A-485) treatment (14) (Figure 2G) and without overt DNA damage (Figure 2H). We also expressed RAS as positive control for accumulation of DNA damage during OIS (1,4).

Analysis of SES status in response to CDK4i revealed that H3K27ac levels are increased at *VEGFC* and *SOD2* loci (SES under HDAC4 regulation) and reduced by the concomitant HDAC4 overexpression (Figure 2I). Accordingly, the corresponding mRNAs are increased by palbociclib and repressed by HDAC4 overexpression (Figure 2J). For *AKR1E2* SES, a strong reduction of H3K27ac was observed in the presence of HDAC4, whereas CDK4i did not significantly increase H3K27ac levels in the analyzed region. The corresponding mRNA was upregulated by palbociclib and repressed by HDAC4 (Figure 2J). *IL1B* expression was not induced by CDK4i and H3K27ac levels remained largely unchanged (Figure 2I/J). H3K27ac levels were reduced in the analyzed region of CXCL8 SES in HDAC4-expressing cells. Accordingly, HDAC4 repressed *CXCL8* expression (Figure 2I, J). Overall, these results suggest that the activity of HDAC4 on SES is important in determining senescence independently from RS.

### Class IIa HDACs control RS

**Figure 3.**
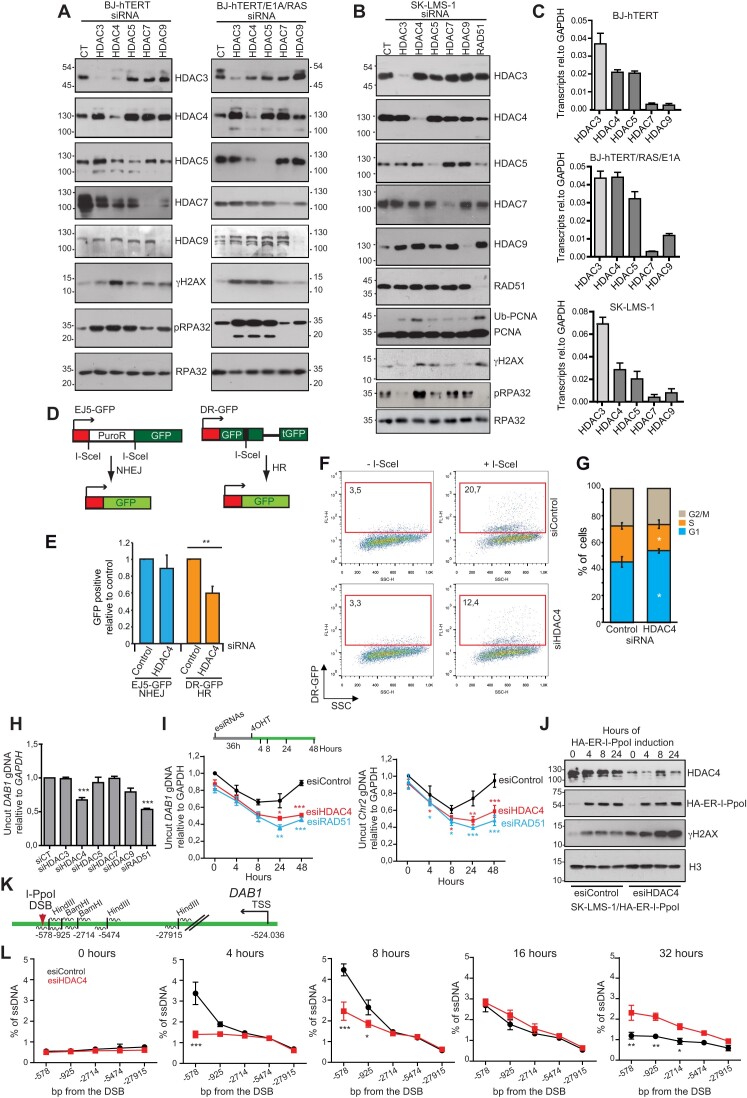
HDAC4 is required for an efficient HR directed repair. (**A**) Immunoblot analysis in BJ-*hTERT* and BJ-*hTERT/E1A* (1–143) cells transfected or not for 72 h with the indicated siRNAs. (**B**) Immunoblot analysis in SK-LMS-1 cells transfected or not for 60 h with the indicated siRNAs. (**C**) Transcript levels of the indicated mRNAs in the examined cell lines with respect to *GAPDH*. Data were retrieved from whole transcriptome analysis (GSE94416 and GSE150427). Mean ± SD; *n* = 3. (**D**) Scheme illustrating the NHEJ and the HR assisted mechanisms that allow the expression of eGFP respectively in U2OS/EJ5 and U2OS/TRI-DR cells after the cleavage by I-SceI. TRI-DR-U2OS cells express a mutant version of GFP with an internal I-SceI endonuclease restriction site, and another GFP mutant with 3′and 5′ end truncations. After I-SceI-induced DSBs, HR can restore wild-type GFP expression (23). In U2OS cells expressing EJ5-GFP, a GFP cassette is separated from its promoter by the puromycin-resistance gene that is flanked by two tandem I-SceI sites. After I-SceI-induced DSBs at these sites, NHEJ repair activities can restore GFP expression. (**E**) U2OS cells expressing the TRI-DR or the EJ5 reporters were transfected with the indicated siRNAs and GFP positive cells were evaluated by cytofluorimetric analysis. Mean ± SD; *n* = 3. *P< 0.05, **P< 0.01 and ***P< 0.001. (**F**) Dot plot representative of four samples of U2OS/TRI-DR cells transfected with I-SceI and esiRNAs against HDAC4 as indicated. (**G**) Cell cycle profile of U2OS/TRI-DR cells, as determined by cytofluorimetric analysis, transfected with the indicated siRNAs. Mean ± SD; *n* = 3. *P< 0.05, **P< 0.01, and ***P< 0.001. (**H**) SK-LMS-1 cells engineered to express in an inducible manner the mega-endonuclease I-PpoI-ER (SK-LMS-1*^HDAC4-/-HDAC4PAM-ER^*) were transfected with the indicated siRNAs. 24 h later I-PpoI was induced and gDNA was extracted after other 48 h. The efficiency of the repair was expressed as rate of *DAB1* levels in respect to CT uncut. A genomic region in the first intron of *DAB1* was amplified in qPCR reaction. The amplified region spans over the I-PpoI cleavage site. Significances refer to siRNA control (siCT) transfected cells. Mean ± SD; *n* = 3. *P< 0.05, **P< 0.01 and ***P< 0.001. **I**. Quantification of genome integrity in correspondence to the I-PpoI cleavage site in the first intron of *DAB1* on chromosome 1 and in an intergenic region of chromosome 2, in respect to *GAPDH* which has no I-PpoI cleavage sites. For this analysis, SK-LMS-1*^HA-ER-I-PpoI^*cells were transfected with the indicated esiRNAs and were harvested at the indicated time after I-PpoI induction (4OHT). Significances refer to siRNA control (siCT) transfected cells. Mean ± SD; *n* = 3. *P< 0.05, **P< 0.01 and ***P< 0.001. (**J**) Immunoblot analysis in SK-LMS-1*^HA-ER-I-PpoI^*cells treated for the indicated hours with 4OHT and transfected with esiRNAs control or anti HDAC4. Cellular lysates were incubated with the indicated antibodies. HA-ER-I-PpoI was detected with an anti-HA antibody. (**K**) Scheme reporting the genomic restriction sites of BamHI and HindIII in proximity to I-PpoI cleavage site on *DAB1* locus and exploited in the ssDNA end-resection essay. For each genomic position a pair of primers was designed to amplify the template in qPCR, and they are indicated as waves in the figure. (**L**) ssDNA end-resection assay performed on the gDNA extracted at the indicated times after I-PpoI induction (+4OHT) in SK-LMS-1*^HA-ER-I-PpoI^*cells transfected with esiRNAs control or anti HDAC4. Mean ± SD; *n* = 3. *P< 0.05, **P< 0.01 and ***P< 0.001.

The involvement of class IIa HDACs in RS is still unexplored. To investigate the contribution of the different class IIa HDACs to RS, we silenced their expression in both immortalized BJ-*hTERT* and transformed BJ-*hTERT/RAS/E1A* cells. These cell lines are commonly used models to study senescence (9,45). Expression of HDAC3, a partner of these deacetylases was also silenced (Figure 3A). Induction of RS can be observed in both transformed and non-transformed cells when HDAC3, HDAC4, and HDAC5 were silenced. In transformed cells, RPA32 phosphorylation is associated to an increase in γHA2X, whereas in normal cells HA2X phosphorylation is not strongly evident until HDAC4 is downregulated. In general, non-transformed cells show much lower levels of RS (Figure 3A). Of note, the silencing of HDAC4 can also decrease the levels of HDAC3 and HDAC5 in a cell line specific manner (Figure 3A). To confirm a role of HDAC3 and HDAC5 in regulating DNA damage and accumulation of RS in these cell lines, endoribonuclease-prepared siRNAs (esiRNAs) were selected. esiRNAs are more specific and have fewer off-target effects compared with siRNAs. In BJ-*hTERT/RAS/E1A* cells, downregulation of HDAC3 and HDAC5 can also increase RS and DNA damage accumulation (Figure 3A and Supplementary Figure S7E).

We repeated this screening in SK-LMS-1 cells. As additional control, we also silenced RAD51, which plays a key role in DSBs repair (46). In this cancer cell line, only HDAC4 silencing resulted in RS. As expected, downregulation of RAD51 leads to RS and, unexpectedly, to HDAC4 and HDAC5 downregulation (Figure 3B). The low expression of HDAC7 and HDAC9 in these cell lines may explain their apparent lack of role in regulating RS (Figure 3C).

### HDAC4 is required for an efficient HR directed repair

The accumulation of DNA damage in response to HDAC4 depletion could be due to both an increase in RS and a deficit in RS resolution. We favour the second hypothesis because downregulation of HDAC4 does not promote cell replication but restricts cell cycle progression. Previous studies have indicated possible contributions of HDAC4 to the DDR, but the mechanisms involved have not been defined (47–51). DSBs repair occurs via two main pathways: non-homologous end joining (NHEJ) and homologous recombination (HR). Alternative EJ repair (Alt-EJ) mechanisms may also be employed (52). To investigate the role of HDAC4 in HR and in NHEJ, we used a well-characterized I-SceI-mediated gene conversion assay (Figure 3D).

HDAC4 knock-down had marginal, not significant effects on NHEJ or Alt-EJ repair, whereas it resulted in a decrease in HR efficiency (Figure 3E, F and Supplementary Figure S7F/G). Downregulation of HDAC4 resulted in a slight accumulation of cells in G1 and a consequent reduction in cells in S phase (Figure 3G).

To confirm the role of HDAC4 in HR, we generated SK-LMS-1 cells expressing site-specific I-PpoI meganuclease, ER and HA-tagged. This allows the introduction of single DSBs into defined regions of the genome (53). The *DAB1* locus was selected to monitor DNA repair. Among the different class IIa HDACs, only silencing of HDAC4 affected DSBs repair (Figure 3H). Of note, the effect of HDAC4 was comparable to that of RAD51. To exclude off-target and lineage-dependent effects, this result was first validated using siRNA and esiRNA against HDAC4 (Supplementary Figure S8A, B) and next verified in BJ-*TERT/RAS/E1A* cells (Supplementary Figure S8D, E). Time course analysis shows that downregulation of HDAC4 affects repair ability rather than DNA damage induction, similarly to RAD51 silencing (Figure 3I/J). While control cells complete DNA repair at the *DAB1* locus within 48 h, cells with downregulated HDAC4 accumulate DSBs with a similar kinetics to control cells, but fail to fully repair the locus (Figure 3I). This effect was confirmed in another region cleaved by I-PpoI characterized by condensed chromatin, as well as in a third locus (*SLC05A1*) that is actively transcribed and also in BJ-*hTERT/RAS/E1A* cells (Supplementary Figure S8C–E). Silencing of HDAC4 or RAD51 showed a similar additive effect when cells were treated with DDR inhibitors Mirin and Scr7 (Supplementary Figure S8F).

DNA end resection is a key aspect of HR. It is initiated by the MRN-CtIP complex to generate a short, exposed 3′-ssDNA end (54). After formation, the 3′-ssDNA end is extended by the action of the EXO1/DNA2-BLM complex to promote RPA-RAD51 switching and HR (55). On the opposite, the Shieldin complex acts by restricting end-resection to promote NHEJ (56). The presence of ssDNA around the DSBs induced by I-PpoI was measured by the DNA end-resection assay (25) (Figure 3K). Depletion of HDAC4 leads to a delay in resection activity at the *DAB1* locus, in regions closer to the brake. Consequently, after 32 h, ssDNA is only detectable in cells where HDAC4 was silenced (Figure 3L). In conclusion, HDAC4 deficiency affects an early phase of HR.

### Reduced efficiency of BRCA1 loading at DSBs in the absence of HDAC4

**Figure 4.**
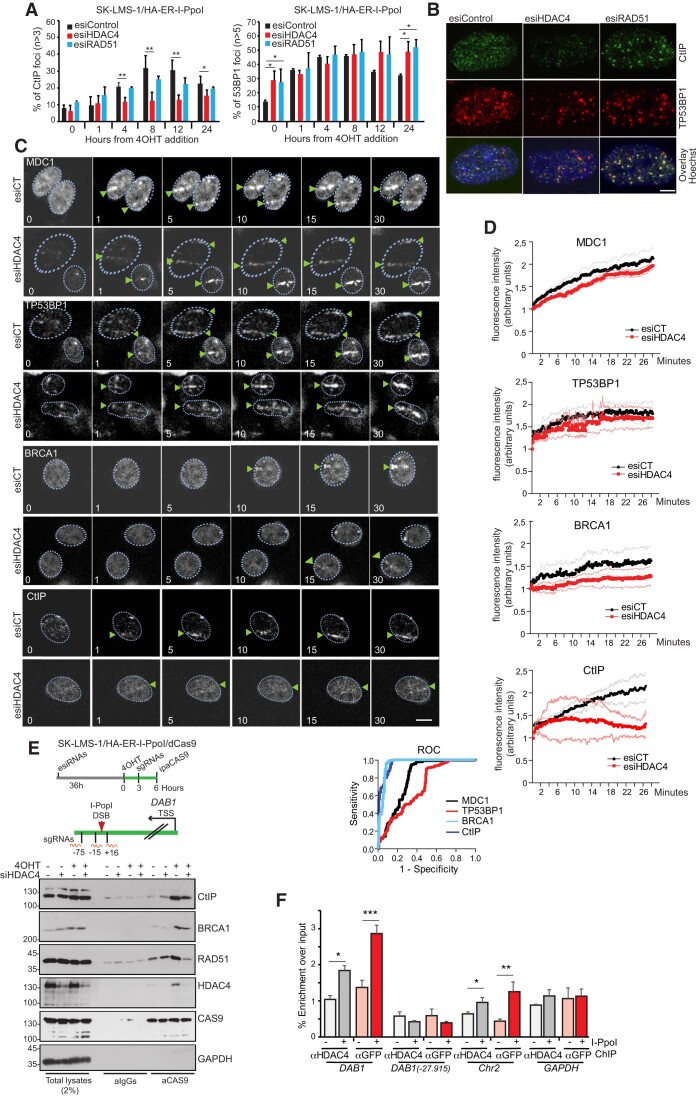
Reduced efficiency of BRCA1 loading at DSBs in the absence of HDAC4. (**A**) Histogram representing the percentage of SK-LMS-1*^HA-ER-I-PpoI^*cells with CtIP foci (*n* > 3 per cell) or TP53BP1 foci (*n* > 5 per cell) at the indicated hours from I-PpoI induction (+4OHT). For each time point at least 200 cells were scored. Mean ± SD; *n* = 3. *P< 0.05 and **P< 0.01. (**B**) Representative images at 8 h after I-PpoI induction of the immunofluorescences used for the quantitative analysis in Figure 5A. Confocal images are shown in pseudocolors. Bar 4.37 μm. (**C**) Time course of recruitment of MDC1, TP53BP1 and BRCA1 in SK-LMS-1 cells silenced for 48 h with esiRNAs against HDAC4 and then subjected to laser microirradiation. The first image shows cells before irradiation, and following images were taken at the indicated time points (minutes) after irradiation. The arrows point to the microirradiation site in each cell leading to accumulation of the indicated DDR proteins (green arrows). Bar 10 μm. (**D**) Graphs displaying the recruitment of MDC1 (*n* = 3, cells analyzed 21), TP53BP1 (*n* = 3, cells analyzed 24), CtIP (*n* = 3, cells analyzed 14) and BRCA1 (*n* = 3, cells analyzed 24), in SK-LMS-1 cells silenced or not for 48 h with esiRNAs against HDAC4. ROC curve demonstrating the superior ability of BRCA1 and CtIP, compared to MDC1 and TP53BP1, in distinguishing control cells from HDAC4 silenced cells, highlighting the defective recruitment of BRCA1 and CtIP in HDAC4 silenced cells. (**E**) CLASP was used to co-purify chromatin associated proteins with dCas9 targeted to the I-PpoI cleavage site at the DAB1 locus in SK-LMS-1*^HA-ER-I-PpoI^*, using sgRNAs as indicated in the scheme. The cells were transfected with the different esiRNAs and then I-PpoI was induced, as shown in the scheme. (**F**) ChIP-qPCR demonstrating the recruitment of HDAC4-GFP in correspondence to I-PpoI cleavage site at the indicated loci. Chromatin was extracted 6 h after I-PpoI induction. Mean ± SD; *n* = 3. *P< 0.05, **P< 0.01 and ***P< 0.001.

A multiprotein complex consisting of BRCA1, CtIP, and MRE11, as part of the MRN-complex (MRE11-RAD51-NBS1) monitors resection of DSBs (57). To further explore the role of HDAC4 in DSBs resection, we compared the time-dependent recruitment of CtIP and the HR antagonist TP53BP1 (58), to sites of DSBs induced by I-PpoI, in cells defective for HDAC4. Silencing of RAD51 was used as control. CtIP foci accumulate in a time-dependent manner after induction of I-PpoI (Figure 4A, B). This accumulation is defective in the absence of HDAC4, whereas it is independent of RAD51 nucleofilament formation (59). As expected, downregulation of HDAC4 or RAD51 increases the number of TP53BP1 foci (Figure 4A, B).

Next, we investigated the recruitment kinetics at DNA damage sites in cells exposed to 405 nm laser micro-irradiation of TP53BP1, BRCA1, CtIP and MDC1. MDC1 is an early recruited assembly factor that binds γH2AX to damaged sites (Figure 4C, arrowheads) (60). Real time signal accumulation shows that the kinetics of TP53BP1 and MDC1 recruitment were independent from HDAC4. In contrast, CtIP and BRCA1, key elements of the HR pathway, are less efficiently recruited in HDAC4 KD cells (Figure 4C, D).

To understand whether HDAC4 is enriched, present, or excluded at sites of DNA damage, we engineered SK-LMS-1 to express endogenous GFP-tagged HDAC4 using PITCh knock-in system (20) (Supplementary Figure S9A, B). Immunofluorescence analysis showed pan-localization of endogenously tagged HDAC4 (Supplementary Figure S9C). As previously reported (26), treatment with the CRM1 inhibitor leptomycin B resulted in nuclear accumulation of HDAC4-GFP. Instead, induction of DNA damage did not overtly affect the nuclear localization of HDAC4 and did not result in a recruitment of HDAC4 to γH2AX foci (Supplementary Figure S9C). To clarify whether a fraction of HDAC4 can localize at the sites of damaged DNA, we used the dCas9-targeted chromatin-based purification strategy (CLASP) (61). The CLASP approach, by delivering three specific sgRNAs into cells that were stimulated to express dCas9 and I-PpoI, allows the purification of chromatin near the I-PpoI cleavage site in the *DAB1* locus (Figure 4E). Induction of I-PpoI expression triggers recruitment of CtIP, RAD51, BRCA1 and HDAC4 in regions near DSBs within the DAB1 locus. HDAC4 silencing attenuated recruitment of all three elements of the HR pathway (Figure 4E).

To further support this observation, we used the I-PpoI system and evaluated the presence of HDAC4 at the *DAB1* site before and after I-PpoI induction. ChIP experiments using two different antibodies and endogenous GFP-tagged HDAC4 proved the presence of HDAC4 at the site of the lesion (Figure 4F). HDAC4 accumulation was not observed at a site distal to the lesion. However, it was clearly detected at a second site cleaved with I-PpoI (Chr2). The *GAPDH* locus was used as a negative control (Figure 4F). In summary, transient localization of HDAC4 near DSBs appears to be an important event for efficient recruitment of the HR complex.

### HDAC4 influences the epigenetic landscape at DSBs

The DDR is sustained and accompanied by local rearrangement of the epigenome (37,62). HDAC4 could affect the DDR at multiple levels, including regulation of genes involved in the DDR. However, analysis of the transcriptome of HDAC4 depleted LMS cells did not evidence any significant perturbation of genes involved in HR (Supplementary Table S2). Therefore, epigenomic surveillance activity might be among the most plausible action. Specifically, H4K16 acetylation increases in response to DSBs, and this acetylation promotes HR-mediated repair by recruiting BRCA1 while limiting TP53BP1 binding to the site of lesion (63,64). In addition, H2BK120 switches from ubiquitination to acetylation in response to DSBs (65). Therefore, we examined the acetylation status of H2BK120 and H4K16, after I-PpoI induced DSBs in the presence or not of HDAC4. H3K27ac, an indirect substrate of class IIa HDACs (9) was used as a reference.

**Figure 5.**
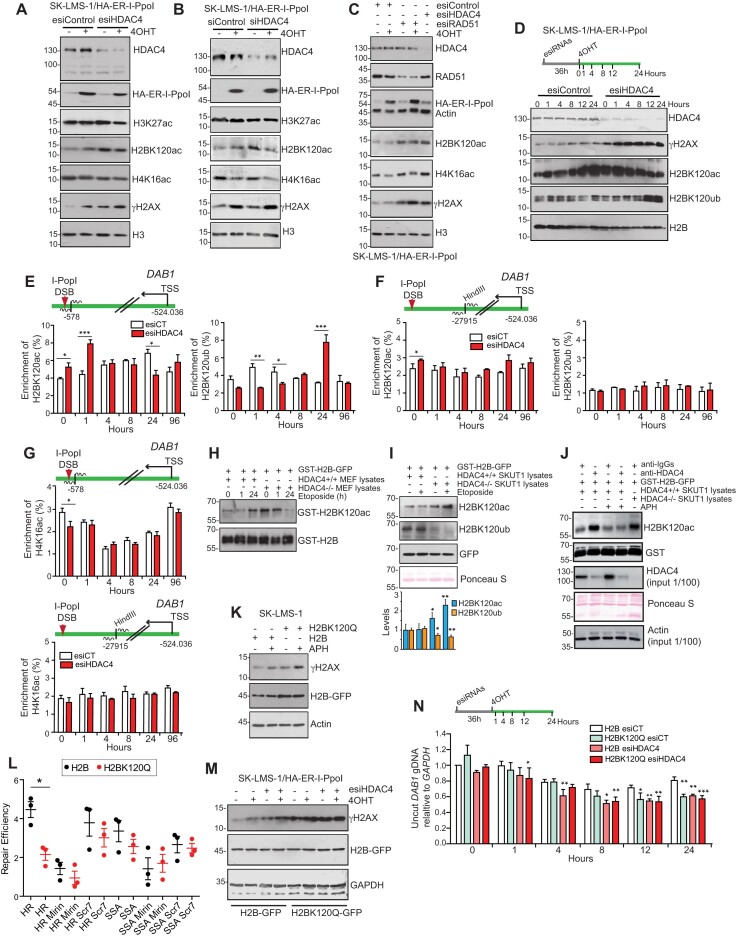
HDAC4 influences the epigenetic landscape at the DSBs. (**A**–**C**) Total cellular lysates were generated from SK-LMS-1*^HA-ER-I-PpoI^*cells silenced or not for HDAC4 or RAD51 for 36 h and then treated or not for 12 h with 4OHT to induce I-PpoI cleavage. Immunoblot analysis was performed using the indicated antibodies. (**D**) Total cellular lysates were generated from SK-LMS-1*^HA-ER-I-PpoI^*cells silenced or not for HDAC4, following the induction of I-PpoI, as indicated in the figure. Immunoblot analysis was performed using the indicated antibodies. (**E**–**G**) ChIP-qPCR were performed to evaluate the H2BK120ac, H2BK120ub and H4K16ac levels through the time following I-PpoI cleavage at the *DAB1* locus, in SK-LMS-1*^HA-ER-I-PpoI^*cells silenced for HDAC4 or control. (**E**) H2BK120ac, H2BK120ub levels were examined in proximity to I-PpoI cleavage site. (**F**) H2BK120ac, H2BK120ub levels were examined distally to I-PpoI cleavage. (**G**) H4K16ac levels were examined both proximally and distally. Mean ± SD; *n* = 3. *P< 0.05, **P< 0.01 and ***P< 0.001. (**H**, **I**) In vitro acetylation and ubiquitylation assay of recombinant GST-H2B-GFP incubated for 30 min. with lysates obtained from MEFs *HDAC4^+/+^* and *HDAC4^−/-^* (**H**) and SKUT1 (**I**) *HDAC4^+/+^* and *HDAC4^−/−^* cells and pretreated or not for the indicated time with etoposide as indicated. (**J**) *In vitro* acetylation and ubiquitylation assay of recombinant GST-H2B-GFP incubated for 30 min. with lysates obtained from SKUT1 *HDAC4^+/+^* and *HDAC4^−/−^*cells, immunodepleted or not for HDAC4 as indicated. (**K**) Total cellular lysates were generated from SK-LMS-1 cells stably over-expressing H2B-GFP and the acetyl-mimicking H2BK120Q-GFP. Immunoblot analysis was performed using the indicated antibodies. Where indicated, 1 μM APH was used. (**L**) gDNA was extracted from U2OS/TRI-DR cells over-expressing H2B or H2BK120Q, transfected with empty plasmid or with I-SceI for 48 h and treated or not for the last 40 h with Scr7 or Mirin (20μM). qPCR on GFP with specific primers was used to discriminate between SSA and HR repair. Repair efficiency was expressed as rate between I-SceI and empty plasmid transfected cells. Mean ± SD; *n* = 3. *P< 0.05. (**M**) Total cellular lysates were generated from SK-LMS-1*^HA-ER-I-PpoI^*cells stably over-expressing H2B-GFP or H2BK120Q-GFP, transfected for 36 h with esiCT and esiHDAC4 and then treated or not for 12 h with 4OHT to induce the expression of I-PpoI. Immunoblot analysis was performed using the indicated antibodies. (**N**) qPCR analysis of *DAB1* locus integrity after the I-PpoI cleavage in SK-LMS-1*^HA-ER-I-PpoI^*cells stably over-expressing H2B-GFP or H2BK120Q-GFP, transfected for 36 h with esiCT and esiHDAC4 and then treated or not for 12 h with 4OHT to induce the expression of I-PpoI. Significances for each time point refer to siRNA control (siCT) transfected H2B expressing cells. Mean ± SD; *n* = 3. *P< 0.05, **P< 0.01 and ***P< 0.001.

I-PpoI induced DSBs cause an increase in H2BK120ac levels and a slight decrease in H4K16ac (Figure 5A, B). Downregulation of HDAC4 causes an increase in H2BK120ac, whereas H4K16ac is minimally affected (Figure 5A, B). After induction of I-PpoI the increase in H2BK120ac was not enhanced by downregulation of HDAC4. A similar increase in H2BK120 acetylation is observed after downregulation of RAD51 (Figure 5C). Time-course analysis confirmed the increase in H2BK120ac in HDAC4-silenced cells before induction of DNA damage. Instead, this acetylation is decreased later after I-PpoI induction, possibly because of the switch to ubiquitylation (Figure 5D). Changes in H2BK120ac and H4K16ac may occur independently of DNA damage. To accurately map these changes in the context of DSBs, ChIP experiments were performed at the *DAB1* locus. In the absence of DNA damage, H2BK120ac levels are higher in HDAC4-KD cells, in a region close to the site of I-PpoI cleavage. When DSBs are generated, there is an early and transient increase in H2BK120ac signal (within 1 hour) in HDAC4-silenced cells, followed by a later transient switch to H2BK120ub. In contrast, control cells show the described ub/ac switch with a peak of H2BK120ub at 1 hour and a later progressive H2BK120ac accumulation (Figure 5E). In a region distal to the DSBs site, no differences between H2BK120ac and H2BK120ub were detected after I-PpoI induction (Figure 5F). The slight downregulation of H4K16ac in HDAC4-silenced cells was confirmed by ChIP. The pattern of H4K16 acetylation after DNA damage shows transient downregulation only near the lesion and is not affected by HDAC4 (Figure 5G). In summary, HDAC4 deficiency causes an alteration in the H2BK120 acetylation/ubiquitylation switch at the site of DSBs.

The role of HDAC4 in buffering H2BK120ac was confirmed in an *in vitro* acetylation/ubiquitylation assay using cell lysates of *Hdac4^+/+^* and *Hdac4^−/−^* MEFs (mouse embryonic fibroblasts), treated or not with etoposide (Figure 5H). Acetylation at K120 of recombinant GST-H2B was strongly observed after incubation with cell lysates from *Hdac4^−/−^* cells. In the presence of Hdac4, treatment with etoposide for 24 h resulted in a comparable increase in GST-H2B acetylation. A similar result was obtained using lysates from LMS SK-UT-1*^HDAC4-/-^*or wt cells as an enzyme source. This experiment also evidenced a decrease in H2BK120ub in the KO cells (Figure 5I). HDAC4 immuno-depletion proved that HDAC4-dependent activity is required to buffer H2BK120 acetylation (Figure 5J). Overall, HDAC4 can enhance deacetylation of H2BK120 and affect HR.

To further corroborate these data, we engineered SK-LMS-1*^HA-ER-I-PpoI^*cells to express a mutant version of H2BK120 (K120Q) (66), which mimics the presence of the acetyl-group (Figure 5K). These cells exhibit higher basal levels of DNA damage and are less able to repair DSBs through HR. By contrast, single-strand annealing (SSA) was minimally perturbed (Figure 5L) (67). The MRN complex inhibitor Mirin (68) and the DNA ligase IV inhibitor Scr7 (69) were used as controls. We then used the same cells to study the effect of silencing HDAC4 on repairing the *DAB1* locus. Levels of γH2AX in response to I-PpoI induction or HDAC4 silencing were increased in cells expressing H2BK120Q (Figure 5M). Interestingly, the accumulation of unrepaired DNA at the *DAB1* locus was comparable between cells silenced for HDAC4 or expressing H2BK120Q, and these effects were not addictive (Figure 5N).

In conclusion, perturbing H2BK120ac dynamics is associated with the accumulation of unrepaired DNA, and this appears to be a major epigenomic change associated with the HR-repair deficit in HDAC4-depleted cells.

### Class I HDACs associate with HDAC4 and provide the H2BK120 deacetylase activity

HDAC4 has low catalytic activity and performs deacetylation by association with class I HDACs (70). The data shown in Figure 2D suggest that the NCOR1/NCOR2/HDAC3 complex may be involved in the regulation of DNA damage by RS. To determine which HDAC4 complex affects H2B120ac, the effects of SAHA/vorinostat, a pan-HDACI, and PCI34051, an HDAC8-specific inhibitor, were examined. SAHA affected the efficiency of DNA repair at the *DAB1* locus and increased H2BK120ac. PCI34051 induced DNA damage and increased H2B120ub but did not affect H2BK120ac levels (Supplementary Figure S10A, B).

**Figure 6.**
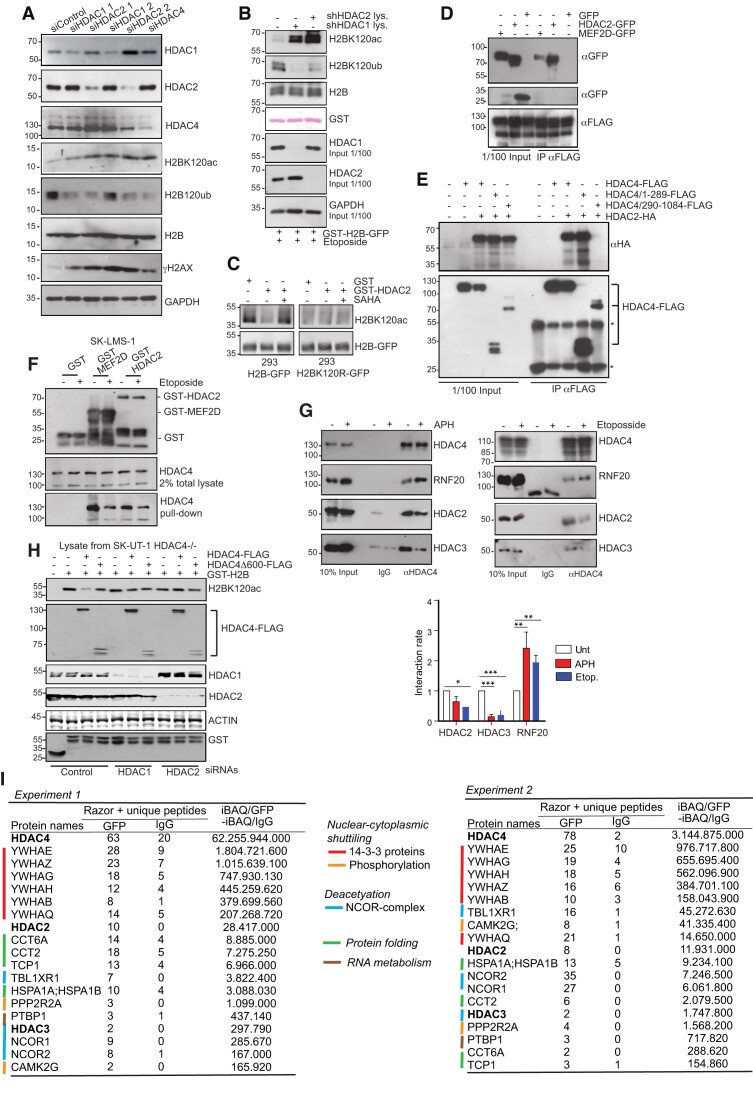
HDAC1 and HDAC2 associate with HDAC4 and are necessary to control H2BK120 acetylation. (**A**) Total cellular lysates were generated from SK-LMS-1 cells transfected for 48 h with the indicated siRNAs. Immunoblot analysis was performed using the indicated antibodies. (**B**) *In vitro* acetylation and ubiquitylation assay of recombinant GST-H2B-GFP incubated for 30 min. with total cellular lysates obtained from SK-LMS-1 cells stably knocked-down for HDAC1 or HDAC2 and pretreated for 60 min. with etoposide. Input from SK-LMS-1 lysates was taken to demonstrate the effectiveness of the knock-down. Immunoblot analysis was performed using the indicated antibodies with exception of GST that is visualized by Ponceau S staining. (**C**) *In vitro* deacetylation assay performed on H2B-GFP and H2BK120C-GFP immunoprecipitated from transfected 293T cells in the presence of recombinant GST-HDAC2 or GST alone. SAHA (20 μM) was used to block HDAC2 activity. Immunoblot analysis was performed using the indicated antibodies. (**D**) Pull-down assay was performed by incubating HDAC4-FLAG purified from 293 cells with GFP, MEF2D-GFP and HDAC2-GFP, purified from retrovirally transduced Ampho cells. A specific interaction was observed between HDAC4-FLAG and MEF2D-GFP and HDAC2-GFP. (**E**) 293 cells were co-transfected with plasmid expressing HDAC2-HA and full-length or deletion mutants of HDAC4-FLAG as indicated. Immunoprecipitation was performed by using 1 μg of anti-FLAG antibody. 1/100 total lysate was included as input. (**F**) GST pull-down assay performed by using HDAC4 as prey and GST, GST-MEF2D (aa 1–190) and GST-HDAC2 as baits. HDAC4 was obtained from SK-LMS-1 lysates treated or not for 60 min. with etoposide, as indicated. (**G**) Co-IP experiment in SK-LMS-1*^HDAC4-GFP/HDAC4-GFP^*treated or not with etoposide (60 min.) or APH (4 h) as indicated. Native lysates were immunoprecipitated with 1 μg anti-HDAC4 antibody. Densitometric analysis for three experiments is reported. Mean ± SD; n = 3. *P< 0.05, **P< 0.01 and ***P< 0.001. (**H**) *In vitro* deacetylation assay was performed by using as enzymatic sources proteins obtained from native lysates harvested from SKUT-1*^HDAC4-/−^* cells, re-expressing the indicated mutant of HDAC4-FLAG and silenced or not for HDAC1 or HDAC2 (48 h). Recombinant H2B-GST was incubated for 30 min. at 37°C in reaction buffer with equal lysates amount per each condition and subjected to SDS-PAGE. Immunoblots were performed using the indicated antibodies. **I**. Mass spectrometric analysis of HDAC4-interacting proteins. Shown are the results of two independent biological replicates. Positive hits were selected if the number of unique Razor + peptides in the anti-GFP immunoprecipitations was >2-fold compared to the anti-IgGs immunoprecipitations. Positive hits were ranked as the ratio between the iBAQ scores of anti-GFP and anti-IgGs immunoprecipitations.

HDAC3 is the strongest candidate for providing the enzymatic activity to modulate H2BK120ac. However, silencing HDAC3 does not increase the occurrence of DSBs (Supplementary Figure S10C) and does not alter the kinetics of DSBs repair at the *DAB1* locus in this model (Supplementary Figure S10D). We next investigated the involvement of HDAC1/2. Silencing of these class I HDACs increased H2BK120ac, decreased H2BK120ub signals, and induced DSBs similar to HDAC4 silencing (Figure 6A). Cell lysates depleted of HDAC1 or HDAC2 show increased H2BK120 acetylation and decreased H2BK120 ubiquitylation (Figure 6B). Recombinant HDAC2 deacetylates H2B at lysine 120, and this effect was blocked by SAHA (Figure 6C). A complex between ectopically expressed HDAC4 and HDAC2 can be detected in Co-IP experiments. Binding to MEF2D was used as a control (Figure 6D). The amino-terminal region (aa 1-289) of HDAC4 can bind to HDAC2 (Figure 6E). The interaction between HDAC4 and HDAC2 was also observed in GST pull-down experiments (Figure 6F). Interestingly, induction of DNA damage reduces the association of HDAC4 with MEF2D and HDAC3 (Figure 6F). The existence of a complex between HDAC2 and HDAC4 and its modulation in response to DNA damage and RS was confirmed at the level of endogenous proteins (Figure 6G). The intensity of the interaction was comparable to that with HDAC3, which also decreased in response to treatment with etoposide or APH. In contrast, binding with RNF20, the E3 ligase for H2BK120ub, was increased (Figure 6G). Although some data suggest autonomous functions of HDAC1 and HDAC2, these class I HDACs are often part of the same multiprotein complexes (71). Accordingly, silencing of HDAC1 increased the levels of H2BK120ac. Moreover, a complex between HDAC4 and HDAC1 can be detected in 293 cells with ectopically expressed HDAC4. An interaction was also observed in SK-LMS-1 cells with the endogenous proteins (Supplementary Figure S10E/F).

To prove that an HDAC4-HDAC1/HDAC2 complex is involved in the control of H2BK120ac, GST-H2B was incubated with lysates of HDAC4 KO cells silenced or not for HDAC1 or HDAC2 and re-expressing HDAC4-FLAG, HDAC4Δ600-FLAG (lacking the 484 carboxy-terminal amino acids including the HDAC domain), and FLAG as control (Figure 6H). Re-expression of full-length HDAC4 reduced the amount of acetylated H2BK120. The HDAC4Δ600 only minimally affected H2BK120ac in agreement with its inability to repress RS (Figure 2D). Silencing of HDAC1 and HDAC2 significantly reduced the ability of ectopically expressed HDAC4 to deacetylate H2BK120. Therefore, HDAC1 and HDAC2 appear to play a key role in determining the deacetylation activity of HDAC4 towards H2BK120ac.

To further confirm the existence of an HDAC4-HDAC2 complex and to gain insight into the molecular definition of this complex, co-immunoprecipitation followed by mass spectrometry analysis (Co-IP-MS) was performed (Figure 6I and Supplementary Table S3). SK-LMS-1*^HDAC4/GFP-PITCH^*cells were used for the immuno-purification of HDAC4 complexes. In both experiments, the major proteins interacting with HDAC4 were members of the 14–3-3 family, confirming the robustness of our proteomic analysis. Other HDAC4 interactors were calcium/calmodulin-dependent protein kinase II gamma (CAMK2G) and protein phosphatase 2 regulatory subunit Bα (PPP2R2A), two regulators of HDAC4 nuclear-cytoplasmic shuttling (72,73). Of the known HDAC4 partners, several components of the NCOR1-NCOR2-HDAC3 complex were isolated. HDAC2 was also isolated, paradoxically with a higher iBAQ value compared to HDAC3. In both experiments, HDAC2 proved to be an important partner of HDAC4. Other identified partners of HDAC4 are some chaperones and PTBP1, which is involved in the regulation of mRNA metabolism (74).

In summary, HDAC4 can form a complex with HDAC2 and possibly also with HDAC1 to monitor H2BK120 deacetylation.

### The genomic influence of HDAC4 on DNA damage accumulation and H2BK120 acetylation during RAS-induced senescence

**Figure 7.**
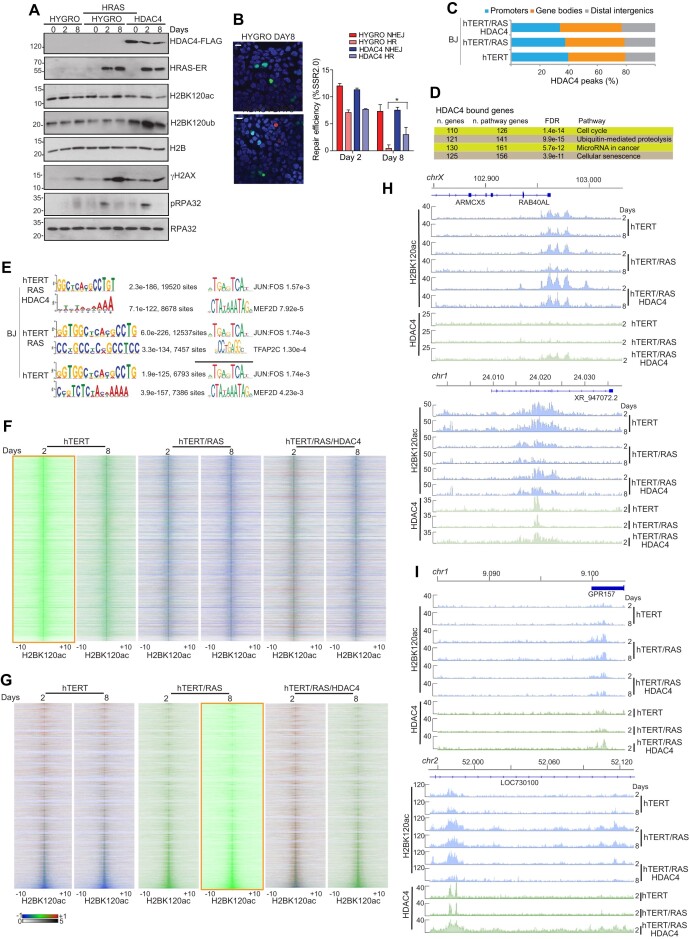
The accumulation of DNA damage during OIS. (**A**) Total cellular lysates were generated from BJ-*hTERT* cells expressing for the indicated days HRASG12V-ER and HYGRO or HDAC4 as indicated. Immunoblot analysis was performed using the indicated antibodies. (**B**) I-SceI was transfected in the indicated BJ-*hTERT/RAS* cells that integrates SSR2.0 48 h before the induction with 4OHT. For quantification, at least 150 cells were analyzed in each of the three biological replicates. Representative confocal images are included (green = NHEJ, red = HR). Bar 25 μm. (**C**) Histogram representing the genomic distribution of HDAC4 IDR peaks in the three cell lines used. We defined: promoters (≤3 kb from TSS), gene bodies (exons, introns and UTRs) or distal intergenic regions (≥3kb from gene boundaries). (**D**) HDAC4 peaks shared between the three cell lines analyzed were investigated for KEGG pathways enrichment. FDR and k/k (bound genes/pathway genes) are shown. (**E**) Motif discovery analysis reporting the 2 most frequent motifs bound by HDAC4 in the three cells lines (left). For each motif, the most similar motif of JASPAR database is reported with its significance. (**F**) Heatmap showing the intensity (represented in shades of red, blue and green) and density (proportional to color gradation) of H2BK120ac peaks in the indicated cell lines compared to BJ-*hTERT* at day 2. The signals are within 20kb of the center of the peaks identified in BJ-*hTERT*. (**G**) Heatmap depicting the intensity (represented in shades of red, blue and green) and density (proportional to the color gradation) of H2BK120ac peaks in the indicated cell lines compared to BJ-*hTERT/RAS* at day 8. Signals are within 20kb of the center of the peaks identified in BJ-*hTERT/RAS*. (**H**, **I**) Representative loci of H2BK120ac and HDAC4 ChIP-seq signals in the three BJ cell lines used at the indicated time points after seeding (BJ-*hTERT*) or RAS induction (BJ-*hTERT/RAS* and BJ-*hTERT/RAS/HDAC4*).

To understand the dynamics of H2BK120ac during OIS and its relationship to DDR, we decided to map the genomic distribution of H2BK120ac in the BJ*-hTERT* model, in response to HRAS^G12V^ induction. It is well known that oncogenic HRAS^G12V^ expression in human fibroblasts can trigger replication stress, the accumulation of irreparable DNA damage and the premature cell cycle exit. A cellular response defined as OIS. (1,4,14). Therefore, BJ-cells were engineered to express HRAS^G12V^-ER following the administration of 4OHT. We also evaluated the influence of HDAC4 by stable overexpression and the HYGRO resistance cassette was used as a control. Analyses were performed 2 days after HRAS^G12V^ induction, when cells are in proliferative phase and RS is increased, and at day 8, when OIS is established (14). Ectopic expression of HDAC4 in BJ-*hTERT/RAS* cells partially rescued the H2BK120 ubiquitylation/acetylation switch and DNA damage accumulation (Figure 7A). Then, to monitor the efficiency of HR and NHEJ, a previously described fluorescent genomic reporter (75) was integrated into the cells. I-SceI was introduced into cells after 2 or 8 days of RAS expression for 48 h. Cells undergoing successful NHEJ after I-SceI cleavage begin to express the GFP protein, whereas cells using HR recombine the repair allele and express the RFP gene. Again, ectopic expression of HDAC4 was sufficient to, at least partially, overcome the HR deficit observed in senescent cells (Figure 7B).

Next, we performed ChIP-seq experiments (Supplementary Figure S11). We first mapped HDAC4 binding on day 2 after RAS induction or not. HDAC4 was found to bind a relevant number of common regions in the three BJ models with a high frequency at promoters and gene bodies (Figure 7C). The genes associated with these genomic loci are involved in the regulation of key processes such as the cell cycle, the ubiquitin-proteasome system, miRNA biogenesis and cellular senescence. A consistent number of HDAC4-bound genes was previously characterized as HDAC4 targets by transcriptomic analysis (Figure 7D and Supplementary Figure S12). In the three cell models examined, HDAC4 binding sites are highly enriched in AP-1 and MEF2 motifs (Figure 7E). Of interest, HDAC4 binding to MEF2 motifs was enriched in BJ-*hTERT* and BJ-*hTERT/RAS/HDAC4* cells but not in BJ-*hTERT/RAS* cells (Figure 7E).

We then examined the three cell lines for H2BK120 acetylation. In agreement with previous studies (76), the genomic distribution of H2BK120ac was similar in the three cell lines and was mainly found at promoters and gene bodies (72.4–75.4% of peaks, Supplementary Table S4).

The H2BK120ac regions frequently overlap between the three cell lines, with the highest value in BJ-*hTERT/RAS* and BJ-*hTERT/RAS/HDAC4* after 2 days of RAS expression (73.86%+0.82) and the lowest (45.53%+3.13) between BJ-*hTERT* and BJ-*hTERT/RAS* at day 8 (Supplementary Table S5).

To better understand the variations in the H2BK120 acetylome induced by RAS, we performed a quantitative analysis of H2BK120 signals by comparing the three cell lines under two different growth conditions. First, we selected all significantly H2BK120-acetylated peaks in BJ-*hTERT* cells at day 2 as a reference for non-transformed proliferating cells (Figure 7F and Supplementary Figure S13A). With few exceptions, a predominant deacetylation of these regions was observed in BJ-*hTERT/RAS* cells, which became even more pronounced after 8 days of RAS induction. Ectopic expression of HDAC4 did not counteract this deacetylation trend, but it was attenuated. This was particularly evident in BJ-*hTERT/RAS/HDAC4* cells 2 days after RAS induction (Figure 7F and Supplementary Figure S13A). Two selected loci in Figure 7H show RAS-induced H2BK120 deacetylation and HDAC4 antagonistic activity. Interestingly, these two loci are characterised by the binding of HDAC4, suggesting its direct involvement in antagonising RAS-induced modifications in the H2BK120ac acetylome.

The second comparison referred to the OIS status reached by the BJ-*hTERT/RAS* cells on day 8 after RAS induction. In this case, significantly H2BK120-acetylated peaks in senescent BJ-*hTERT/RAS* cells showed different acetylation profiles in BJ-*hTERT*. These differences were generally conserved between days 2 and 8 (Figure 7G and Supplementary Figure S13B). Re-expression of HDAC4 in BJ-*hTERT/RAS* resulted in a profile of H2BK120 acetylation that partially matched that of BJ-*hTERT* cells, particularly at 2 days after RAS activation. In Figure 7I, two loci illustrate this behaviour, demonstrating the direct binding of HDAC4 and the dynamic regulation of H2BK120 acetylation.

Altogether, these data show that induction of RAS and entry into senescence cause a profound restructuring of the genomic distribution of H2BK120ac peaks. In BJ-*hTERT/RAS/HDAC4* cells, HDAC4 binding correlated with the maintenance of a hypoacetylated state in these genomic regions compared to BJ-*hTERT/RAS* cells, similar to proliferating BJ-*hTERT* cells.

### H2BK120ac profiles in DNA damaged regions accumulated during OIS: HDAC4 contribution

**Figure 8.**
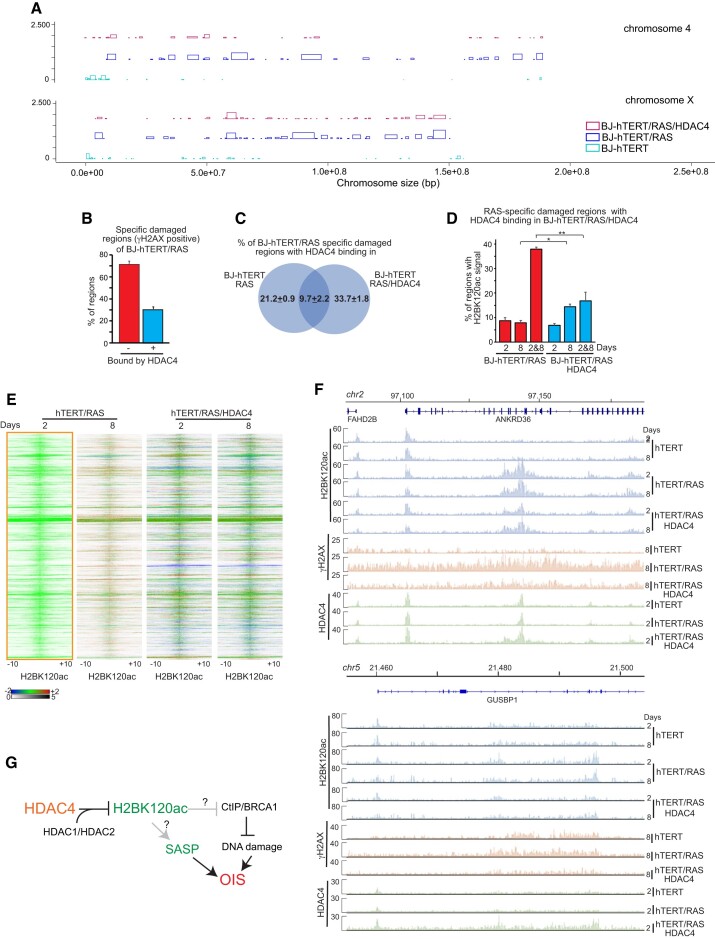
The genomic characterization of HDAC4 and H2BK120ac dynamic during OIS and DNA damage. (**A**) Genomic distribution of the γH2AX signal between BJ-*hTERT*, BJ-*hTERT/RAS* and BJ-*hTERT/RAS/HDAC4* cells induced with 4OHT for 8 days in Replicate A. Two representative chromosomes are shown. (**B**) Histogram representing the percentage of γH2AX regions in BJ-*hTERT/RAS* cells (day 8 of RAS induction) characterized by HDAC4 binding, as indicated. (**C**) Venn diagram depicting the percentage of γH2AX regions in BJ-*hTERT/RAS* cells (day 8) characterized by HDAC4 binding in BJ-*hTERT/RAS* or BJ-*hTERT/RAS/HDAC4* cells. (**D**) Histogram representing the percentage of γH2AX regions in BJ-*hTERT/RAS* cells characterized by HDAC4 binding in BJ-*hTERT/RAS/HDAC4* cells (the 33.7% of regions indicated in C in the Venn diagram) showing significant H2BK120 acetylation at day 2, 8 or day 2 and 8 of RAS induction in the indicated BJ cell lines. Mean ± SD; *n* = 3. *P< 0.05, **P< 0.01. (**E**) Heatmap depicting the intensity (represented as shades of red, blue and green) and density (proportional to the color gradation) of H2BK120ac peaks in the indicated cell lines compared to BJ-*hTERT/RAS* at day 2. Signals are within 20 kb of the center of HDAC4 binding in BJ-h*TERT/RAS/HDAC4* cells. (**F**) Representative loci of H2BK120ac, γH2AX and HDAC4 ChIP-seq signals in the three BJ cell lines used at the indicated time points after seeding (BJ-*hTERT*) or RAS induction (BJ-*hTERT/RAS* and BJ-*hTERT/RAS/HDAC4*). (**G**) Proposed mechanism: HDAC4 proteasomal degradation in presenescent cells leads to dismantling of the HDAC4-HDAC1/HDAC2 complex causing subversion of H2BK120 acetylation/ubiquitylation rate and defective HR-repair.

OIS is associated with unresolved DNA damage and persistent DDR activation (14). To map the genomic distribution of DNA damage during OIS, ChIP-seq for γH2AX (77) was performed in the three cell lines at 8 day from RAS activation. BJ-*hTERT* cells were used as control. As previously observed (61), non-transformed BJ-*hTERT* cells accumulate DNA lesions mainly in sub-telomeric regions, with minor signals eventually spreading along the chromosomes (Figure 8A and Supplementary Figure S14-17). In contrast, expression of RAS results in expansion of γH2AX domains, which are often distributed throughout chromosomes (Figure 8A and Supplementary Figures S14–S17). In the presence of ectopic HDAC4, the pattern of RAS-induced γH2AX domains is maintained but clearly reduced. Furthermore, the γH2AX domains are larger in RAS expressing cells in respect to proliferating cells, while HDAC4 re-expression reduce their length (Supplementary Table S6).

Genomic analysis confirmed that HDAC4 can reduce the accumulation of DNA damage. The following question concerns the status of H2BK120 acetylation in unrepaired genomic regions, as defined by γH2AX positivity. To answer this question, we selected regions that were enriched in γH2AX only in senescent BJ*-hTERT/RAS* cells. In principle, these regions should manifest differences in H2BK120 acetylation compared to BJ-*hTERT/RAS/HDAC4* cells, in which the same regions are not significantly enriched with γH2AX signals. We have defined these regions as RSD (Ras-Specific-Damaged) regions. In BJ*-hTERT/RAS* cells more than 70% of RSD-regions were depopulated by HDAC4 peaks (Figure 8B). Notably, 34% of the RSD regions retained HDAC4 binding (at least one peak) in BJ-*hTERT/RAS/HDAC4* and were not enriched in γH2AX (Figure 8C). These regions could be defined as undamaged or successfully repaired after HDAC4 expression. Interestingly, a high percentage of H2BK120 acetylation characterizes these regions in BJ*-hTERT/RAS* both in the proliferative phase and upon reaching OIS (Figure 8D, days 2 and 8). This high percentage of H2BK120ac is reduced in the presence of HDAC4 (Figure 8D). Quantitative analysis of H2BK120 acetylation in RSD regions showed an overall increase in H2BK120 acetylation in senescent BJ-*hTERT/RAS* cells compared to proliferative phase (Figure 8E, day 2 for reference). In cells re-expressing HDAC4, the RSD regions show a general trend towards deacetylation of H2BK120, although some exceptions can be seen at a few loci. This deacetylation is already clearly visible at day 2 (Figure 8E and Supplementary Figure S3C). As shown by the example genomic loci, there is significant deacetylation of H2BK120ac and a decrease in γH2AX levels after HDAC4 binding in BJ-*hTERT/RAS/HDAC4* cells compared to BJ-*hTERT/RAS* cells (Figure 8F).

In summary, although ChIP-seq only provides a snapshot of the chromatin state of the cells and no conclusions can be drawn about causality, our analysis highlights how, in the genomic regions where DNA double-strand damage accumulated exclusively in BJ-*hTERT/RAS* cells, maintaining HDAC4 binding through its ectopic expression reduces H2BK120ac levels and correlates with a decrease in γH2AX signals.

## Discussion

In this manuscript, we have described the existence of a deacetylase complex formed by HDAC4 and class I HDAC1/HDAC2 that is involved in the recognition and erasing of H2BK120 acetylation. The existence of the HDAC4-HDAC2 complex was confirmed by Co-IP-MS. This analysis did not reveal the presence of co-repressors defining the different multi-subunit complexes in which HDAC1/2 can be recruited and activated. Instead, the association of HDAC4 with the NCOR1-NCOR2 complexes was confirmed. Therefore, it is possible that the ‘classical’ HDAC2 complexes are not involved in the interaction with HDAC4. It will be important to understand whether HDAC4 can act as a platform for the assembly and activation of HDAC2 or whether the NCOR complexes are involved. Interestingly, the N-terminal region of HDAC3 (aa 9–49), which is involved in the binding of NCOR2 and Ins(1,4,5,6)P4, shows high homology to HDAC2 (78).

H2BK120ac preferentially marks promoters and gene bodies (76) and by controlling RNA PolII elongation (79) supervises differentiative (80) and adaptative responses (81). H2BK120ac also plays a role in OIS where it controls transcription of SASP genes, further linking HDAC4 to this epigenetic modification (81). Indeed, we found strong correlations between genomic regions bound by HDAC4 and the presence of H2B120ac signals. Oncogenic programs triggered by HRAS^G12V^ expression can abruptly reset the genomic architecture of H2K120 acetylation. In this context, HDAC4 has a minor antagonizing activity that becomes more evident in regions where DNA damage accumulates (see below).

H2BK120 is also ubiquitylated, and removal of the acetyl group is instrumental for the epigenetic switch between acetylation and ubiquitylation. Accumulation of H2BK120ub introduces important structural changes and decompaction of higher-order chromatin structure (82). The H2BK120 acetylation/ubiquitylation switch has also been reported in association with DSBs. Paradoxically both an increase in H2BK120 ubiquitylation (83) and an increase in K120 acetylation (65) have been correlated with subsequent successful repair of the lesion. The experiments performed here with the mega-endonuclease I-PpoI demonstrate that control cells ubiquitylate H2BK120 within 1 hour from cleavage, which is later converted to acetylation. In contrast, silencing of HDAC4 results in H2BK120 acetylation that peaks at 1 hour and is replaced by ubiquitylation with delayed kinetics that reaches the ubiquitylation peak at 24 h after I-PpoI cleavage.

The H2BK120 acetylation/ubiquitylation switch is controlled by the SAGA complex (84). At DSBs, PCAF acetylates H2BK120 and converts H2BK120ub into H2BK120ac with the associated DUBs USP22 and USP11 to promote DSBs repair. However, unlike HDAC4, both HR and NHEJ are impaired in the absence of PCAF or USP11 (84,85).

Although further experiments are required to precisely define the composition of the HDAC1/HDAC2 complexes that interact with HDAC4, our results suggest that this complex, by controlling the deacetylation of H2BK120, may affect the H2BK120 ac/ub switch. Thereby these observations suggest a link between HDAC4/HDAC1/2 activities and the SAGA-complex. Several lines of evidence support this conclusion: i) depletion of HDAC4 increases H2BK120ac and interferes with H2BK120ub deposition, ii) ectopically expressed HDAC4 increases H2BK120ub deposition during OIS, iii) the E3 ligase RNF20 can be co-immunoprecipitated with HDAC4, iv) genomic binding of HDAC4 is highly enriched in regions marked by H2BK120ac. The binding of HDAC4 to active chromatin also underpins a role in HR (65,86).

The HDAC4/HDAC1/2-dependent alteration of the H2BK120 acetylation/ubiquitylation switch correlates with defective and delayed recruitment of CtIP and BRCA1 to damaged sites and defects in DNA end-resection. In this context, BRCA1 promotes HR by overcoming TP53BP1-mediated blockade of CtIP (58). In the absence of HDAC4, HR-repair slows down while NHEJ is not altered, and cells accumulate TP53BP1 foci and nuclear bodies and reduce proliferation. Indeed, re-expression of HDAC4 in BJ-*hTERT/RAS* cells results in a partial escape from senescence that correlates with restored HR-repair and reduced size and spread of γH2AX-positive regions. It is plausible that the slowing, rather than blocking, of HR, as a consequence of proteasomal degradation of HDAC4 in presenescent cells, is part of a physiological mechanism that leads to a lack of compensatory activation of error-prone NHEJ or Alt-EJ. A condition that differs in cancer cells (87).

By focusing the analysis on the RSD (Ras-Specific-Damaged) genomic regions of senescent cells, we discovered that the RSD regions accumulate H2BK120 acetylation during senescence and that re-expression of HDAC4 can counteract this accumulation. This activity correlates with the reduction of DNA damage. Since HDAC4-dependent deacetylation in RSD regions is already observed on day 2, when cells proliferate, a possible role of HDAC4-dependent H2BK120 deacetylation in the prevention of DNA damage should be investigated.

Finally as previously published, loss of epigenetic control during DDR in presenescent cells due to UPS-mediated degradation of HDAC4 is coupled to the release of SEs regulated by the HDAC4-HDAC3 complex (9). The integration of the two responses by a single epigenetic actor, HDAC4, is a common feature with other epigenetic regulators, such as EZH2 (88), SIRT6 (89), KDM5A (90), NSD1 (91). Thus, proteasomal degradation of HDAC4 during senescence can be viewed as part of an epigenetic clock that synchronizes the accumulation of unrepaired RS with the transcriptional reprogramming that controls senescence. When this control mechanism is disrupted, genomic instability increases, and neoplastic transformation may occur. In conclusion, our data suggest that the alterations in HDAC4, observed in different cancer types (72), should also be considered in terms of defects in the DDR and consequent potential combinatorial therapeutic interventions.

## Supplementary Material

gkae501_Supplemental_Files

## Data Availability

All data needed to evaluate the conclusions in the paper are present in the paper and/or the Supplementary Materials. All unique reagents generated in this study are available with a completed Materials Transfer Agreement. Raw data corresponding to ChIP-seq experiments in BJ cells and SK-LMS-1 cells are uploaded with GEO accession GSE216677 and GSE216861, respectively. A Superseries containing both SubSeries exists with GEO accession GSE216862. The mass spectrometry proteomics data have been deposited to the ProteomeXchange Consortium via the PRIDE partner repository with the dataset identifier PXD051316 and 10.6019/PXD051316.
