# Supplementary material for: HDAC4 influences the DNA damage response and counteracts senescence by assembling with HDAC1/HDAC2 to control H2BK120 acetylation and homology-directed repair
Source: Nucleic Acids Res. 2024 Jun 14;52(14):8218–40. doi: 10.1093/nar/gkae501 (PMC11317144; doi:10.1093/nar/gkae501)
Supplement: gkae501_Supplemental_Files [file gkae501_supplemental_files.zip › SUPPLEMENTARY DATA.pdf]

SUPPLEMENTARY DATA

HDAC4 influences the DNA damage response and counteract senescence by assembling with HDAC1/HDAC2 to control H2BK120 acetylation and homology-directed repair.

Eros Di Giorgio[1*], Emiliano Dalla[2], Vanessa Tolotto[2], Francesca D'Este[1,2], Harikrishnareddy Paluvai[2], Liliana Ranzino[2], Claudio Brancolini[2*]

[1]Laboratory of Biochemistry, Department of Medicine, Università degli Studi di Udine, p.le Kolbe 4, 33100 Udine, Italy.
[2]Laboratory of Epigenomics, Department of Medicine, Università degli Studi di Udine, p.le Kolbe 4, 33100 Udine, Italy.

[*]To whom correspondence should be addressed. Tel: ++390432494382; Fax: ++390432494301; Email: claudio.brancolini@uniud.it
Correspondence may also be addressed to Tel: ++390432494382; Fax: ++390432494301; Email: eros.digiorgio@uniud.it

This PDF file contains Figures S1-17 and captions for other supplementary material.

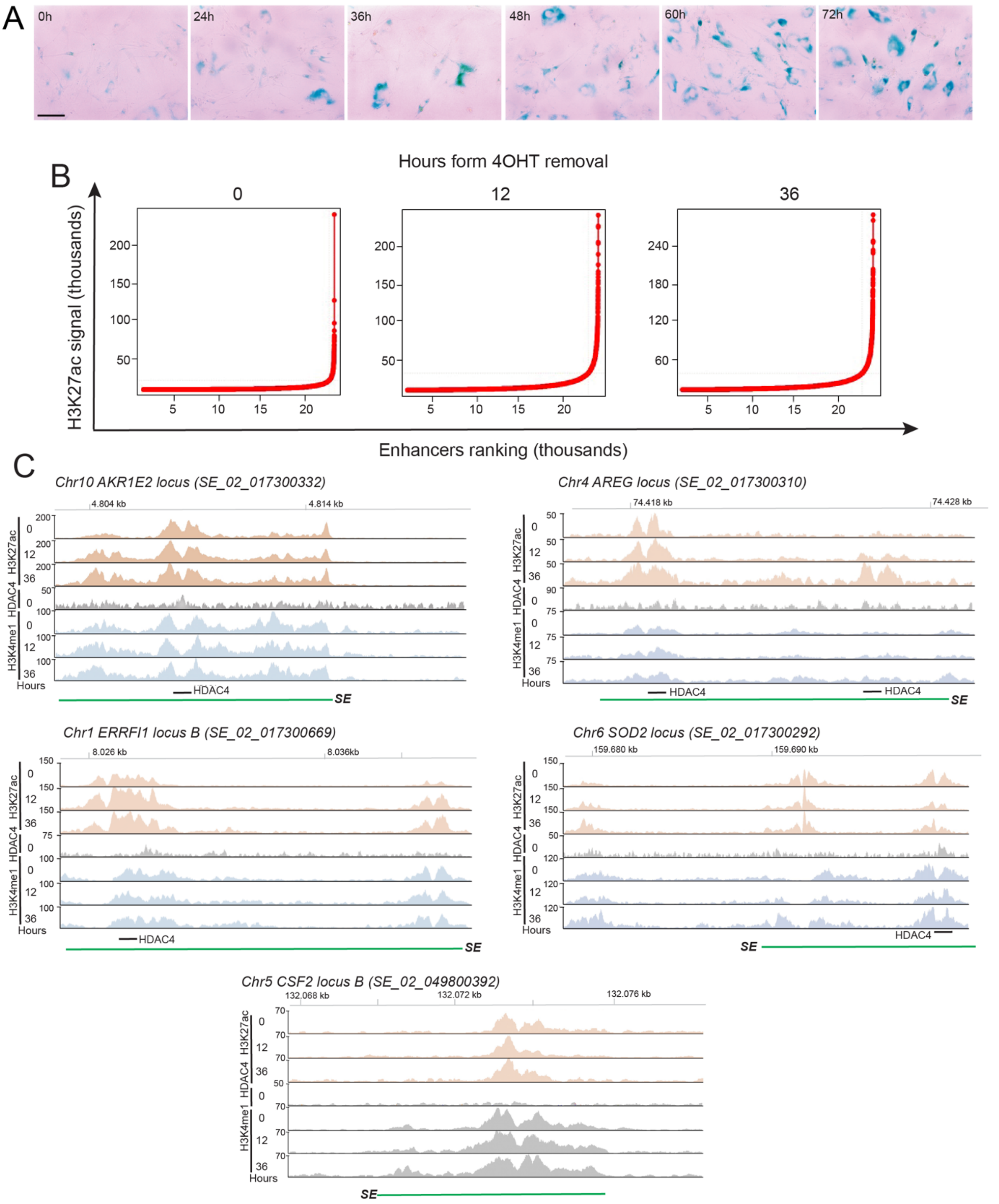

Figure S1. Characterization of senescence properties after HDAC4 depletion in SK-LMS-1 cells.

A. Representative microscopic images of SA-β-gal stained SK-LMS-1$^{HDAC4-/-/HDAC4PAM-ER}$ at the indicated time (hours) after 4OHT removal (Bar = 50μm).

B. SEs regulated by HDAC4 in in SK-LMS-1$^{HDAC4-/-/HDAC4PAM-ER}$ identified at the indicated time (hours) after 4OHT removal. ROSE algorithm ranks enhancers accordingly to H3K27ac levels.

C. Detailed view of representative SES (*AKR1E2, AREG, ERRFI1, SOD2* associated SES) directly bound by HDAC4 in SK-LMS-1 WT and of CSF2, a SASP gene, which SES does not

evidence HDAC4 binding. H3K27ac and H3K4me1 normalized signals and HDAC4 peaks are plotted. ROSE-identified SE and HDAC4 enriched peaks are indicated.

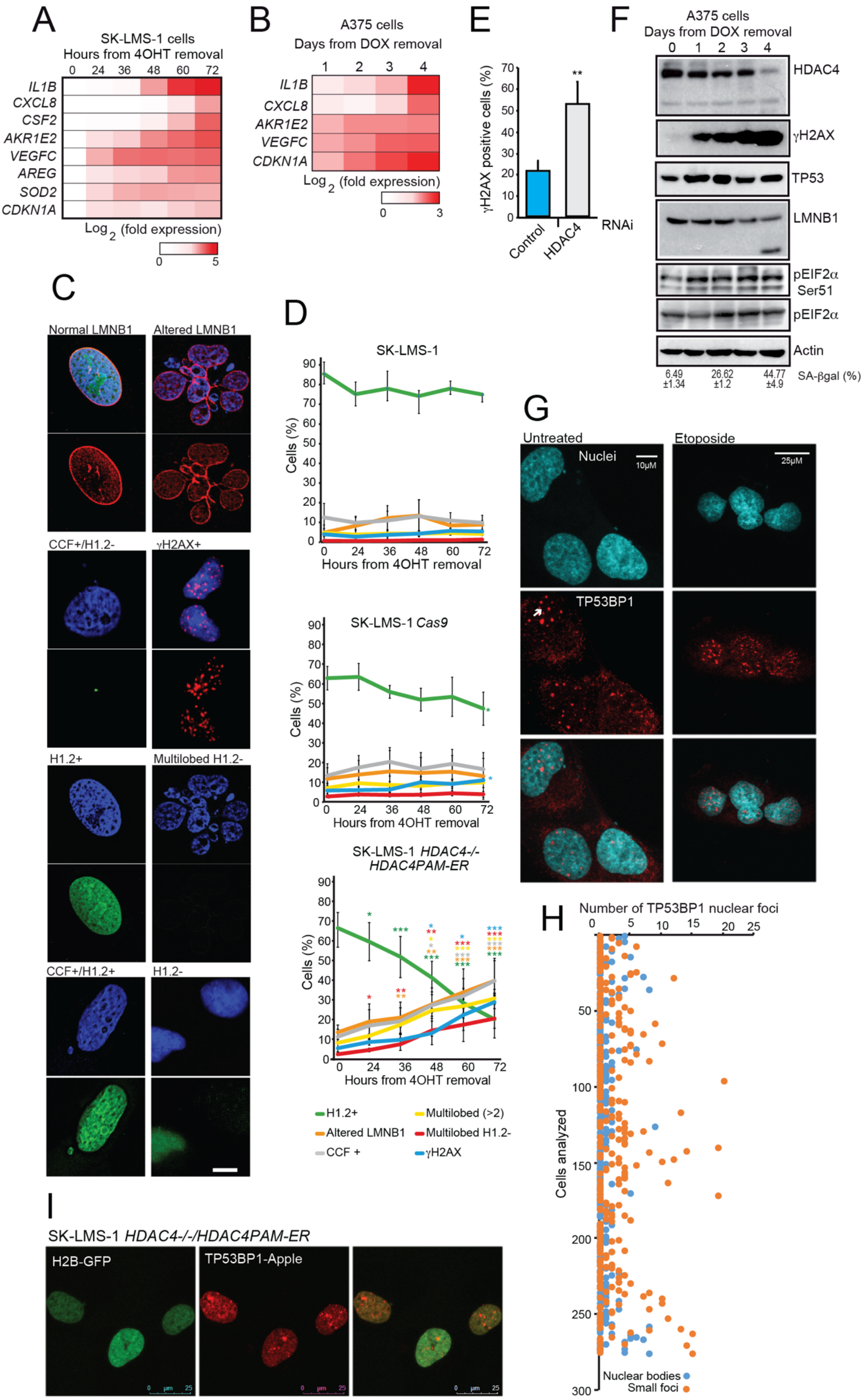

A
SK-LMS-1 cells
Hours from 4OHT removal
0 24 36 48 60 72
IL1B
CXCL8
CSF2
AKR1E2
VEGFC
AREG
SOD2
CDKN1A
Log 2 (fold expression)
0 5
B
A375 cells
Days from DOX removal
1 2 3 4
IL1B
CXCL8
AKR1E2
VEGFC
CDKN1A
Log 2 (fold expression)
0 3
E
γH2AX positive cells (%)
Control
HDAC4
RNAi
**
F
A375 cells
Days from DOX removal
0 1 2 3 4
HDAC4
γH2AX
TP53
LMNB1
pEIF2α Ser51
pEIF2α
Actin
6.49 ±1.34
26.62 ±1.2
44.77 ±4.9
SA-βgal (%)
C
Normal LMNB1
Altered LMNB1
CCF+/H1.2-
γH2AX+
H1.2+
Multilobed H1.2-
CCF+/H1.2+
H1.2-
D
SK-LMS-1
Cells (%)
Hours from 4OHT removal
SK-LMS-1 Cas9
SK-LMS-1 HDAC4-/- HDAC4PAM-ER
H1.2+
Altered LMNB1
CCF +
Multilobed (>2)
Multilobed H1.2-
γH2AX
G
Untreated
Etoposide
Nuclei
10μM
25μM
TP53BP1
H
Number of TP53BP1 nuclear foci
Cells analyzed
Nuclear bodies
Small foci
I
SK-LMS-1 HDAC4-/-/HDAC4PAM-ER
H2B-GFP
TP53BP1-Apple

Figure S2. HDAC4 is required to maintain genome stability.
A. Heatmap representing fold expression of SES associated genes at the indicated time after HDAC4 depletion in SK-LMS-1 cells.
B. Heatmap representing fold induction of super-enhancers associated genes at the indicated days after HDAC4 depletion in $A375^{HDAC4\text{-/-}\ pCW\text{-}HDAC4PAM}$ cells.
C. Representative images of the combination of cellular phenotypes observed after the depletion of HDAC4 in SK-LMS-1 cells (scale bar=10 µm).
D. Quantification of the time-course accumulation of the phenotypes represented in Fig. S2C in the indicated cells. n>200. Mean ± SD; n = 3.
E. Histogram representing the percentage of A375 cells that bears γH2AX foci (n>5 per cell) at 72 hours from HDAC4 silencing. n>200.
F. Lysates of $A375^{HDAC4\text{-/-}\ pCW\text{-}HDAC4PAM}$ cells, were generated at the indicated days after HDAC4 (DOX) removal. Immunoblots were performed using the indicated antibodies. Actin was used as loading control. SA-βgal positivity is indicated.
G. Representative confocal pictures of SK-LMS-1 cells, untreated or etoposide treated (2 hours, 20µM) and immunostained with anti-TP53BP1 and secondary AF546 antibodies (red). DAPI was used to stain nucleic acids (blue). The arrow points to a TP53BP1 nuclear body.
H. Dot plot representing the quantification of DNA damage foci and TP53BP1 bodies in etoposide treated SK-LMS-1 cells (n=270).
I. Representative movie frame of untreated $SK\text{-}LMS\text{-}1^{HDAC4\text{-/-}}$ cells re-expressing HDAC4 engineered to stably express H2B-GFP and Apple-TP53BP1 trunc and subjected to *in vivo* movie microscopy.

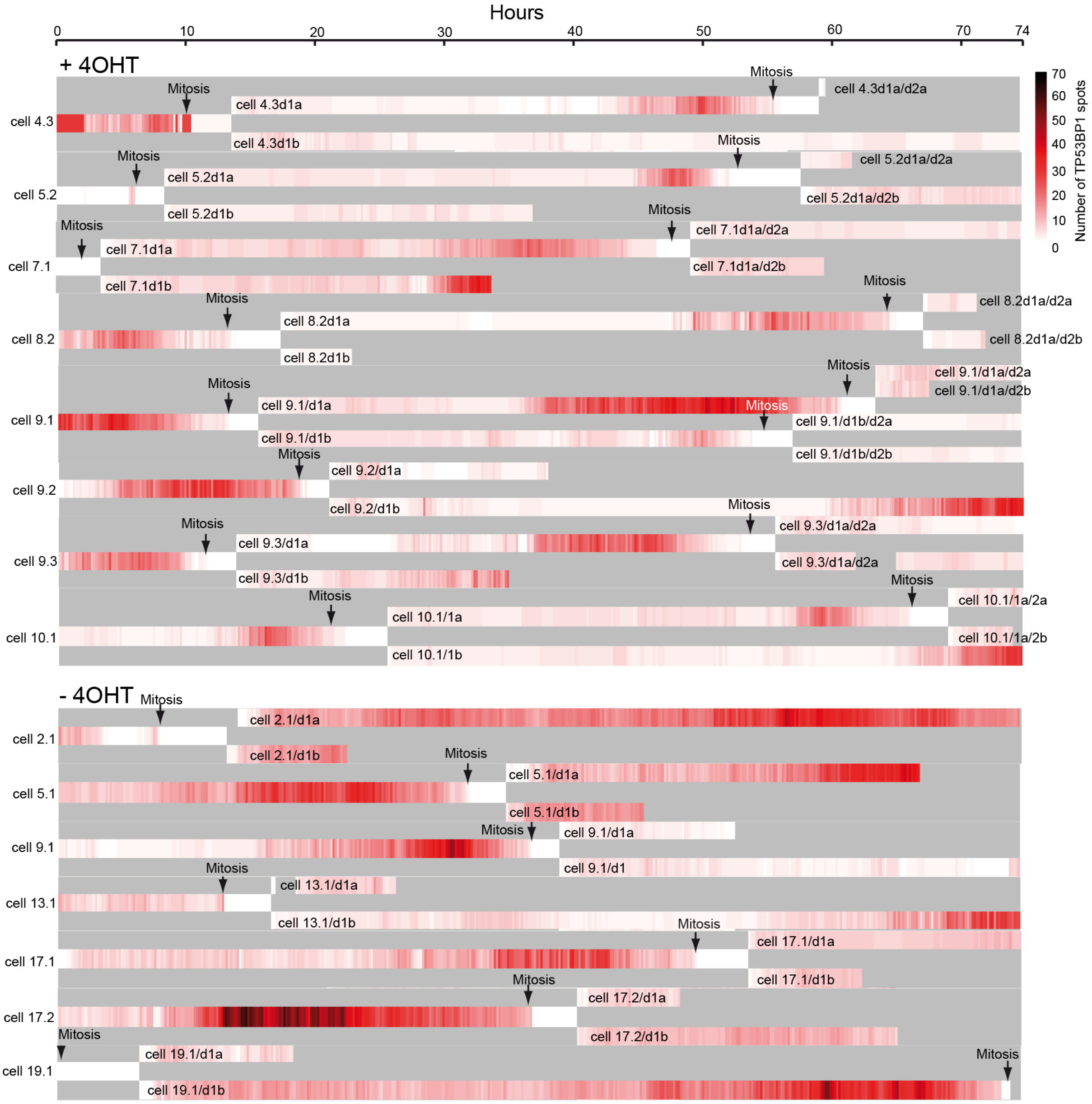

Figure S3. HDAC4 depletion causes the rapid accumulation of TP53BP1 foci in G2 and the subsequent mitotic slowdown and impairment.

Heatmap representing the quantification of the TP53BP1 foci/bodies in SK-LMS-1$^{HDAC4-/-/HDAC4PAM-ER}$. Analyses were performed for 74 hours starting from 6 hour after 4OHT removal (time "0"), as indicated. The intensity of the red signal is proportional to the number of TP53BP1 spots. The beginning of the mitosis is indicated by arrows. The daughter cells arising from each mitosis are indicated and labelled as "d" (daughter). In total, 10 and 9 cells were analyzed respectively for the +4OHT and the -4OHT conditions.

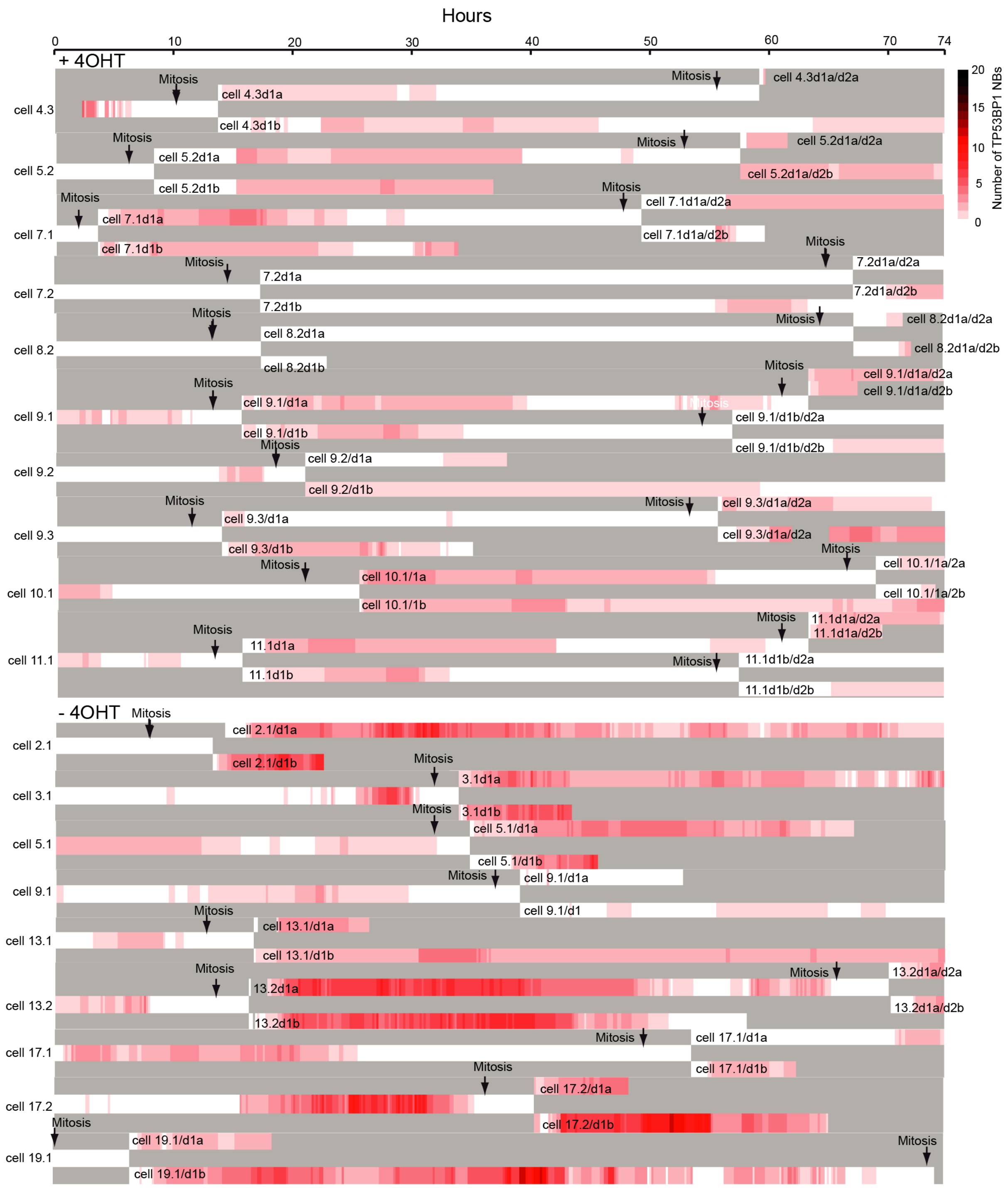

Figure S4. HDAC4 depletion causes the persistent accumulation in post-mitotic cells of TP53BP1 nuclear bodies.

Heatmap representing the quantification of the TP53BP1 nuclear bodies (NB) in SK-LMS-1$^{HDAC4-/-/HDAC4PAM-ER}$, during 74 hours of analysis starting from 6 hour after 4OHT removal (time "0"), as indicated. The intensity of the red signal is proportional to the number of TP53BP1 NBs. The beginning of the mitosis is indicated by arrows. The daughter cells arising from each mitosis are indicated and labelled as "d" (daughter). In total, 10 and 9 cells were analyzed respectively for the +4OHT and the -4OHT conditions.

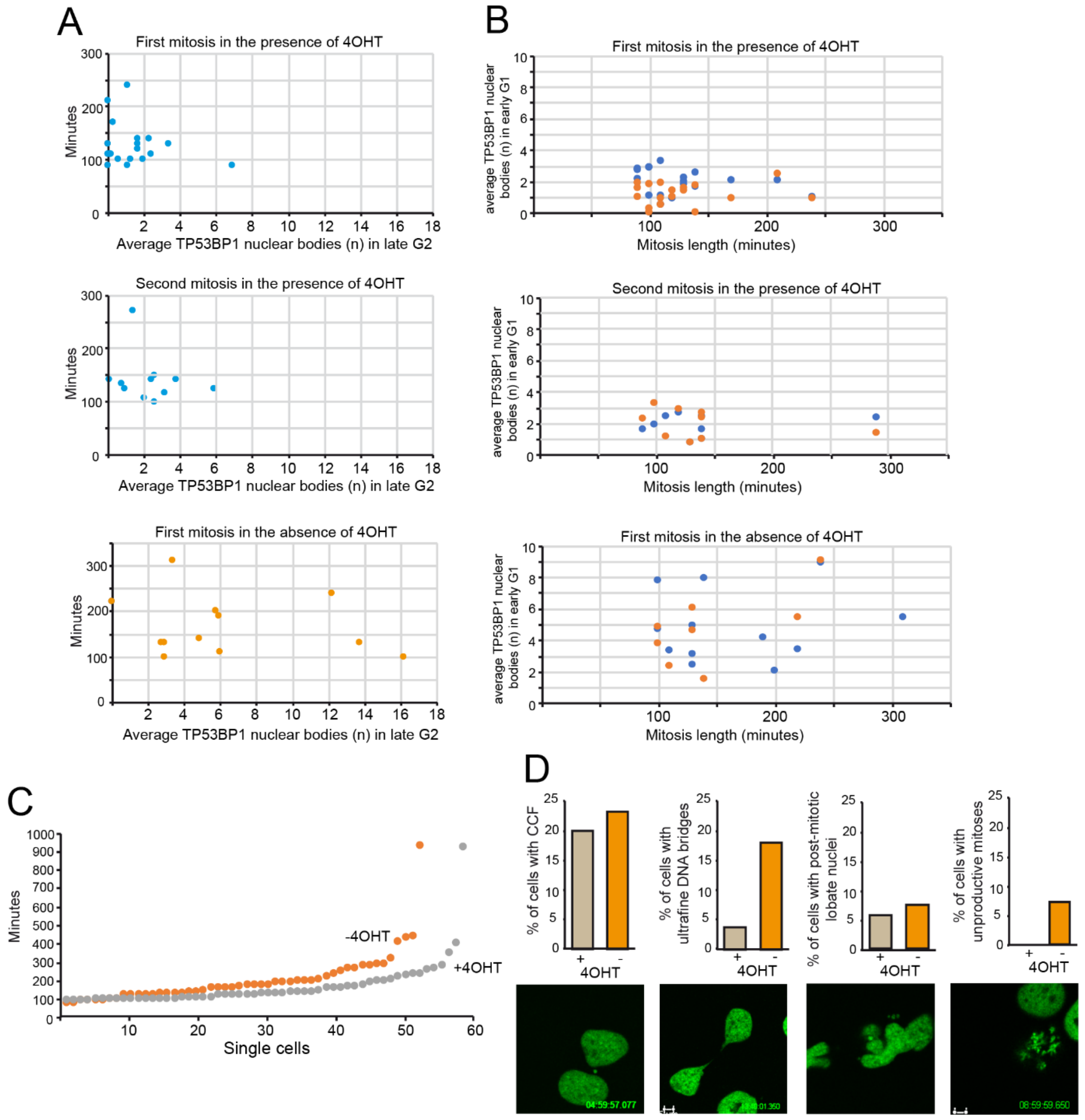

Figure S5. HDAC4 depletion leads to the accumulation of mitotic defects.

A. Dot plot representing the correlation between the duration of the first and second mitosis (y) and the average number of TP53BP1 nuclear bodies (x) observed in the last 10 frames (100 min.) preceding the prophase in SK-LMS-1$^{HDAC4-/-/HDAC4PAM-ER}$ cells re-expressing (+4OHT) or not (-4OHT) HDAC4-ER. The observation period is of 74 hours. Mitosis duration: +4OHT: 1st 127 ± 39.34 min; 2nd 139.09±53.37; -4OHT: 1st 166.67±65.55; 2nd not observed.

B. Dot plot representing the correlation between the duration of the first and second mitosis (x) and the average number of TP53BP1 nuclear bodies (y) observed during the first 10 frames (100 min.) after cytokinesis in sister cells (orange spot: mother cells, blue spot: offspring) subjected to the same observation explained in Fig. S5A.

C. Dot plot representing the duration of mitosis in SK-LMS-1$^{HDAC4-/-/HDAC4PAM-ER}$ re-expressing (+4OHT) or not (-4OHT) *HDAC4*^{PAM}*-ER*.

D. Quantification of mitotic defects evidenced by H2B-GFP and observed in the cells subjected to in vivo movie microscopy as explained in Supplementary Figure S5A, during 74 hours of analysis. For each kind of mitotic defect, a representative movie frame is provided.

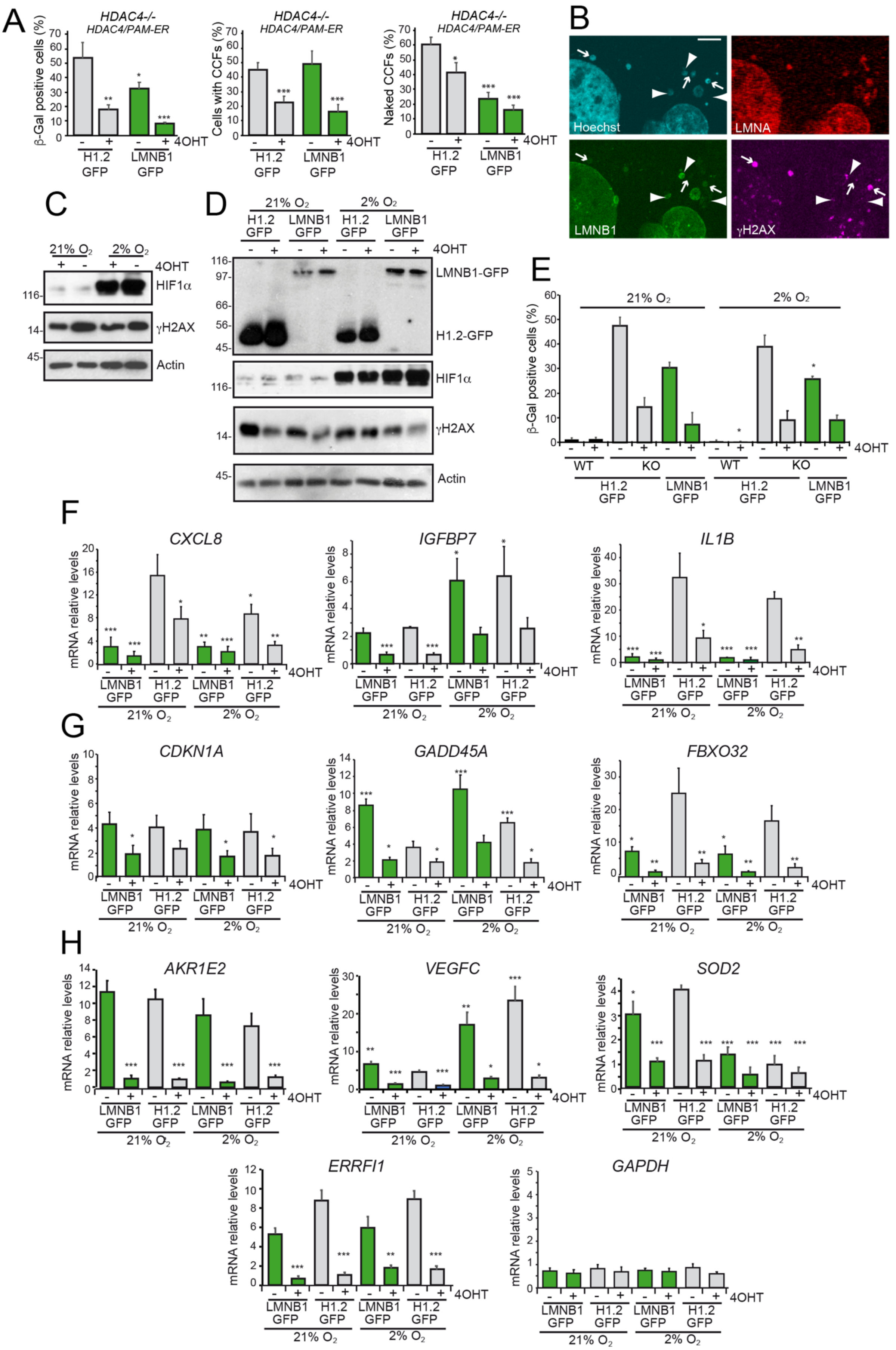

Figure S6. LMNB1 re-expression and hypoxic growing conditions have minimal effects

on the accumulation of DNA lesions during senescence induced by HDAC4 depletion.
A. Analysis of the % of SK-LMS-1$^{HDAC4-/-HDAC4PAM-ER}$ cells, grown in normoxia, expressing H1.2-GFP or GFP-LMNB1, as indicated, and re-expressing (+4OHT) or not (-4OHT) HDAC4PAM-ER, displaying positivity for SA-β-gal or the accumulation of CCFs and naked CCFs. Mean ±SD; n=4. The significance is relative to H1.2-GFP re-expressing cells.
B. Representative confocal picture of SK-LMS-1$^{HDAC4-/-HDAC4PAM-ER}$ cells, expressing GFP-LMNB1 and immunostained for DNA (Hoechst, blue), LMNA (red) and γH2AX (violet). Arrows point to naked CCFs, arrowheads to LMNB1+ CCFs. LMNB1 did not reduce the total number of DNA CCFs, but it reduced the appearance of naked (with defective LMNB1 envelope) CCFs. Naked CCFs were frequently positive for γH2AX.
C. Immunoblot analysis of HIF-1α and γH2AX, in SK-LMS-1$^{HDAC4-/-HDAC4PAM-ER}$ cells, re-expressing (+4OHT) or not (-4OHT) HDAC4PAM-ER and expressing the indicated transgenes. Lysates were generated after 4 days of culture in normoxia or in hypoxia, as indicated.
D. Immunoblot analysis of HIF-1α, γH2AX, GFP-LMNB1 and H1.2-GFP (anti-GFP antibody) in SK-LMS-1$^{HDAC4-/-HDAC4PAM-ER}$ cells, re-expressing (+4OHT) or not (-4OHT) HDAC4PAM-ER and expressing the indicated transgenes. Lysates were generated after 4 days of culture in normoxia or in hypoxia, as indicated.
E. Analysis of the SA-β-gal positivity in SK-LMS-1$^{HDAC4-/-HDAC4PAM-ER}$ cells expressing the indicated genes and knocked out or not for HDAC4. Significances are relative to the same condition (cell line and treatment) in normoxia. Mean ±SD; n=4.
F. mRNA expression levels of SASP genes in SK-LMS-1$^{HDAC4-/-HDAC4PAM-ER}$ cells. Levels are relative to SK-LMS-1 cells grown in normoxia. Significances refer to SK-LMS-1$^{HDAC4-/-HDAC4PAM-ER}$ cells expressing H1.2-GFP and grown in normoxia without 4OHT. Mean ±SD; n=3. *P<0.05, **P<0.01, and ***P<0.001.
G. mRNA expression levels of DDR related genes in SK-LMS-1$^{HDAC4-/-HDAC4PAM-ER}$ cells. Levels are relative to SK-LMS-1 cells grown in normoxia. Significances refer to SK-LMS-1$^{HDAC4-/-HDAC4PAM-ER}$ cells expressing H1.2-GFP and grown in normoxia without 4OHT. Mean ±SD; n=3. *P<0.05, **P<0.01, and ***P<0.001.
H. mRNA expression levels of HDAC4-regulated SES-associated genes in SK-LMS-1$^{HDAC4-/-HDAC4PAM-ER}$ cells. Levels are relative to SK-LMS-1 cells grown in normoxia. LMNB1 has opposite effects on *FBXO32* and *GADD45A* expression during senescence, by limiting and promoting their expression, respectively. The expression of *VEGFC, SOD2*, *IGFBP7* and *GADD45* is also regulated by low oxygen, independently from senescence. Significances refer to SK-LMS-1$^{HDAC4-/-HDAC4PAM-ER}$ cells expressing H1.2-GFP and grown in normoxia without 4OHT. GAPDH was used as control. Mean ±SD; n=3. *P<0.05, **P<0.01, and ***P<0.001.

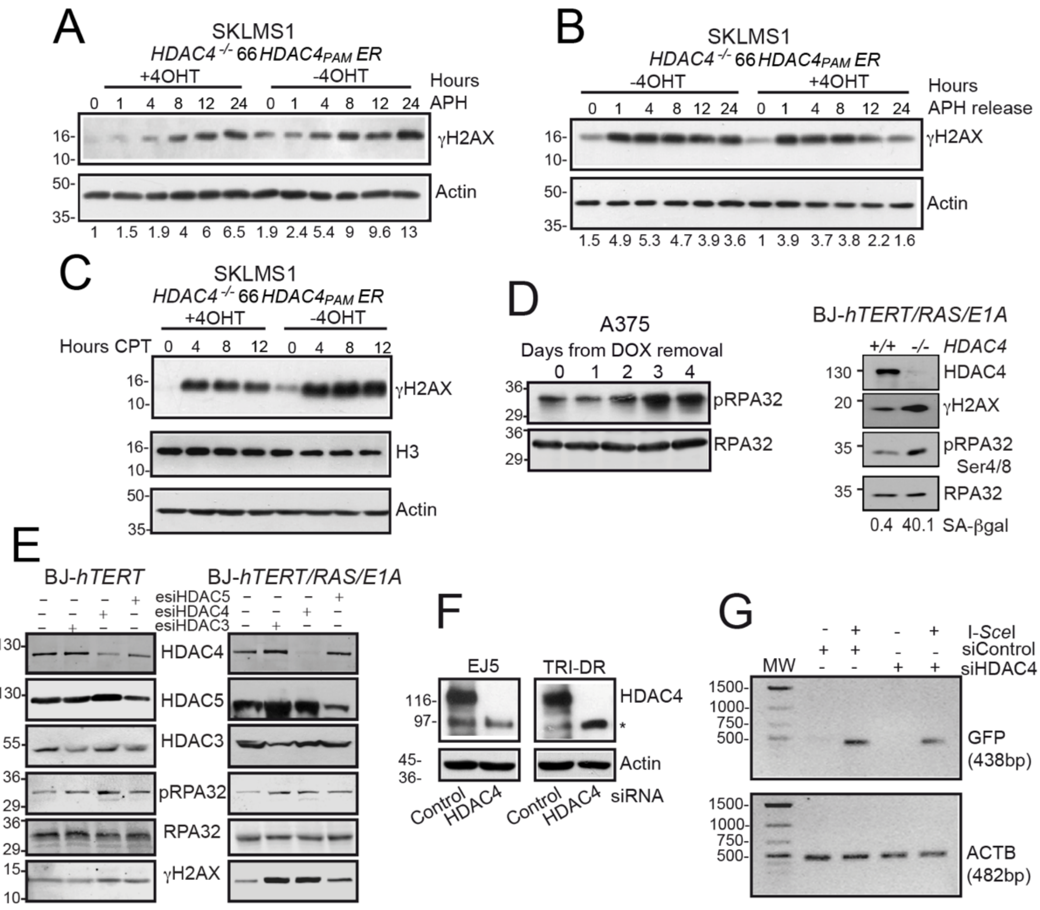

Figure S7. HDAC4 restrains RS and supports the repair by HR.

A and C. Time-course immunoblot analysis in SK-LMS-1$^{HDAC4-/-/HDAC4PAM-ER}$ cells re-expressing or not HDAC4^{PAM}-ER (48 hours) and treated for the indicated time with 500nM APH (A) or 3.125μm CPT (C). Actin was used as loading control. Densitometric analysis of γH2AX/Actin ratio is provided.

B. Immunoblot analysis on the same cells described in Supplementary Figure S7A, harvested at the indicated time after the release from 60 min. APH treatment. Densitometric analysis of γH2AX/Actin ratio is provided.

D. Immunoblot analysis on the indicated cells, harvested at the indicated time (days) after DOX removal (A375 left) or 7 days after the achievement of the knock-out in BJ-*hTERT/RAS/E1A* cells.

E. Immunoblot analysis on the indicated cells transfected for 48 hours with 74pmoles of the indicated esiRNAs. Whole cell-lyase were processed. Total RPA32 was used as loading control.

F. Immunoblot analysis on the indicated cells, silenced or not for HDAC4 and transfected or not with I-*Sce*-I expressing plasmid, as indicated. Actin was used as loading control.

G. PCR was performed by using *GFP* specific primers and as a template the genomic DNA extracted from TRI-DR-GFP cells treated as in Supplementary Figure S7F. The intensity of GFP PCR products is proportional to the efficiency of HR repair. *ACTB* was used as control.

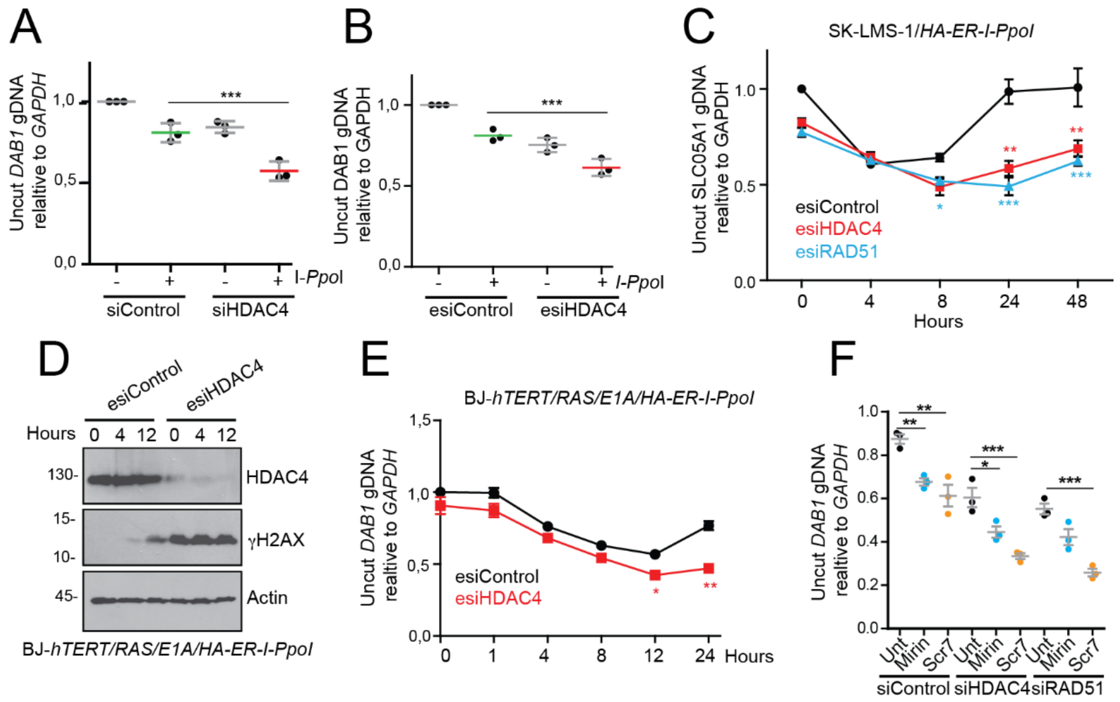

Figure S8. HDAC4 displays a genome caretaker role in different cell lines.

A and B. qPCR analysis of DAB1 integrity after the I-PpoI cleavage in the same cells described in Figure 3I, transfected for 36 hours with siRNAs and esiRNAs against HDAC4 or controls. The analysis was performed at 24 hours from the treatment with 4OHT to induce I-PpoI,

C. qPCR analysis of *SLC05A1* integrity after the I-PpoI cleavage in the same cells described in Fig. 5I and treated with 4OHT to induce I-PpoI as indicated in the figure.

D. Immunoblot analysis in BJ-*hTERT/RAS/E1A/HA-ER-I-PpoI* cells, silenced for 48 hours with the indicated esiRNAs and then treated for the indicated time to induce I-PpoI.

E. qPCR analysis of DAB1 integrity after the I-PpoI cleavage and treated with 4OHT to induce I-PpoI as indicated in the figure. BJ-*hTERT/RAS/E1A/HA-ER-I-PpoI* cells were silenced for 48 hours with the indicated esiRNAs

F. qPCR analysis of DAB1 integrity after the I-PpoI cleavage in SK-LMS-1-*HA/ER/I-PpoI* cells treated for 12 hours with 4OHT to induce I-PpoI. Cells were silenced for 48 hours with the different siRNAs and treated with the different inhibitors of DDR or with DMSO.

In A, B, C, E, and F data are shown as means ± SD; n = 3. *P<0.05, **P<0.01, and ***P<0.001.

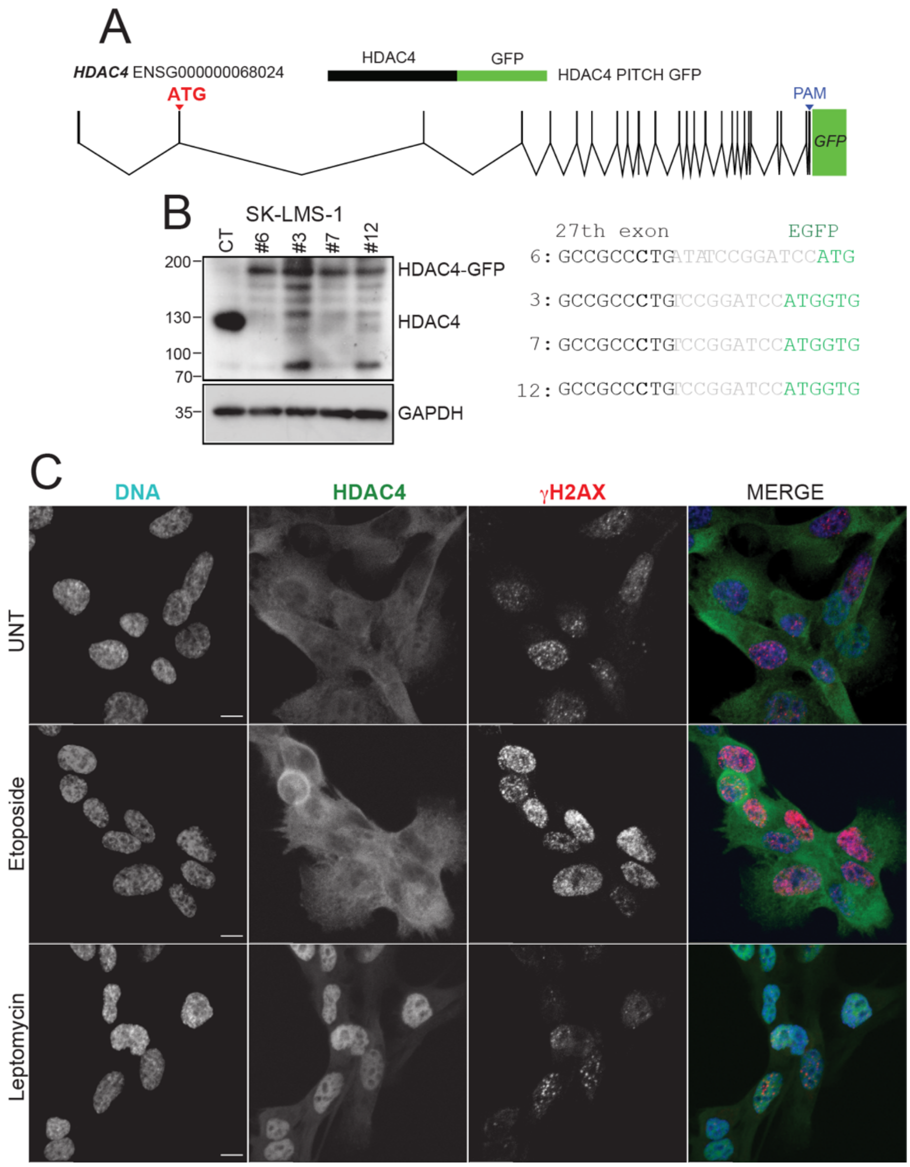

Figure S9. Generation and characterization of SK-LMS-*1*$^{HDAC4\text{-}GFP/HDFAC4\text{-}GFP}$ cells.

A. PITCh technology was used to tag the C-terminus of HDAC4 with eGFP. In the diagram the vertical lines indicate the exons, the oblique lines the introns. The PAM present on the last exon before the stop codon was used for the targeting.

B. Immunoblot analysis and Sanger sequencing of the indicated monoclonal cultures of SK-LMS-*1*$^{HDAC4\text{-}GFP/HDFAC4\text{-}GFP}$ cells. Clones 6 and 7 were used for the experiments.

C. Confocal pictures of SK-LMS-1$^{HDAC4\text{-}GFP/HDFAC4\text{-}GFP}$ clone 6 cells, treated or not as indicated for 60 min. with etoposide (10 µM) or leptomycin B (50 µg/ml). Bar=10µm.

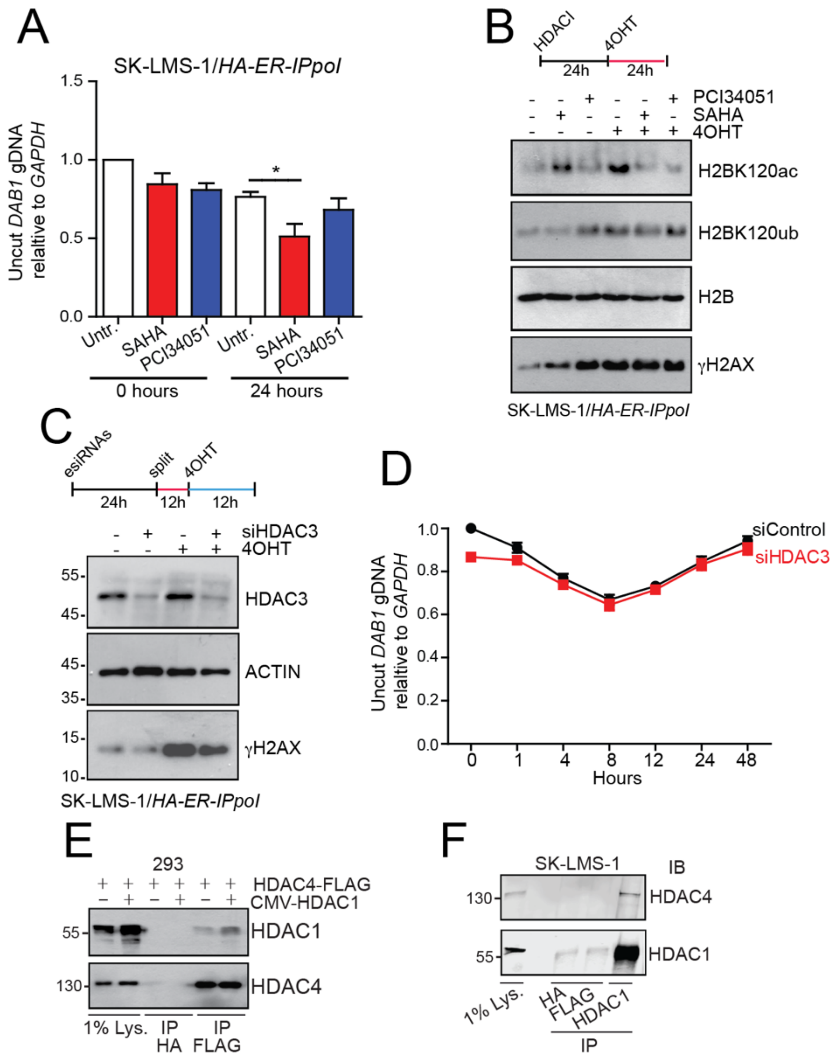

Figure S10. Class I HDACs, but not HDAC3 and HDAC8, control HR-repair and H2B K120ac.

A. qPCR analysis of *DAB1* integrity after the I-*Ppo*I cleavage in SK-LMS-1/*HA-ER-I-PpoI* pre-treated or not for 24 hours with 1µM SAHA or PCI-34051 and then treated for 24 hours with 4OHT to induce I-PpoI.

B. Immunoblot analysis in SK-LMS-1/*HA-ER-I-PpoI*. 1µM SAHA and of PCI-34051 were used. Data are shown as means ± SD; n = 3. *P<0.05.

C. Immunoblot analysis in SK-LMS-1/*HA-ER-I-PpoI* cells transfected with *HDAC3* siRNA or control and treated as indicated in the scheme.

D. qPCR analysis of *DAB1* integrity after the I-*Ppo*I cleavage in SK-LMS-1/*HA-ER-I-PpoI* in which HDAC3 was silenced or not for 36 hours. Data are shown as means ± SD; n = 3.

E. 293 cells were transfected with 4.5 µg of pFLAG-*HDAC4* and 4.5 µg of CMV-Empty of CMV-*HDAC1* plasmids. Native cellular lysates were harvested after 48 hours and immunoprecipitated with 1 µg of anti-HA or anti-FLAG as indicated. 1% Total lysate was loaded as input.

F. Endogenous HDAC1 was immunoprecipitated from exponentially growing SK-LMS-1 cells with 1 µg anti-HDAC1 monoclonal antibody. Anti-HA and anti-FLAG were used as control. 1% Total lysate was loaded as input.

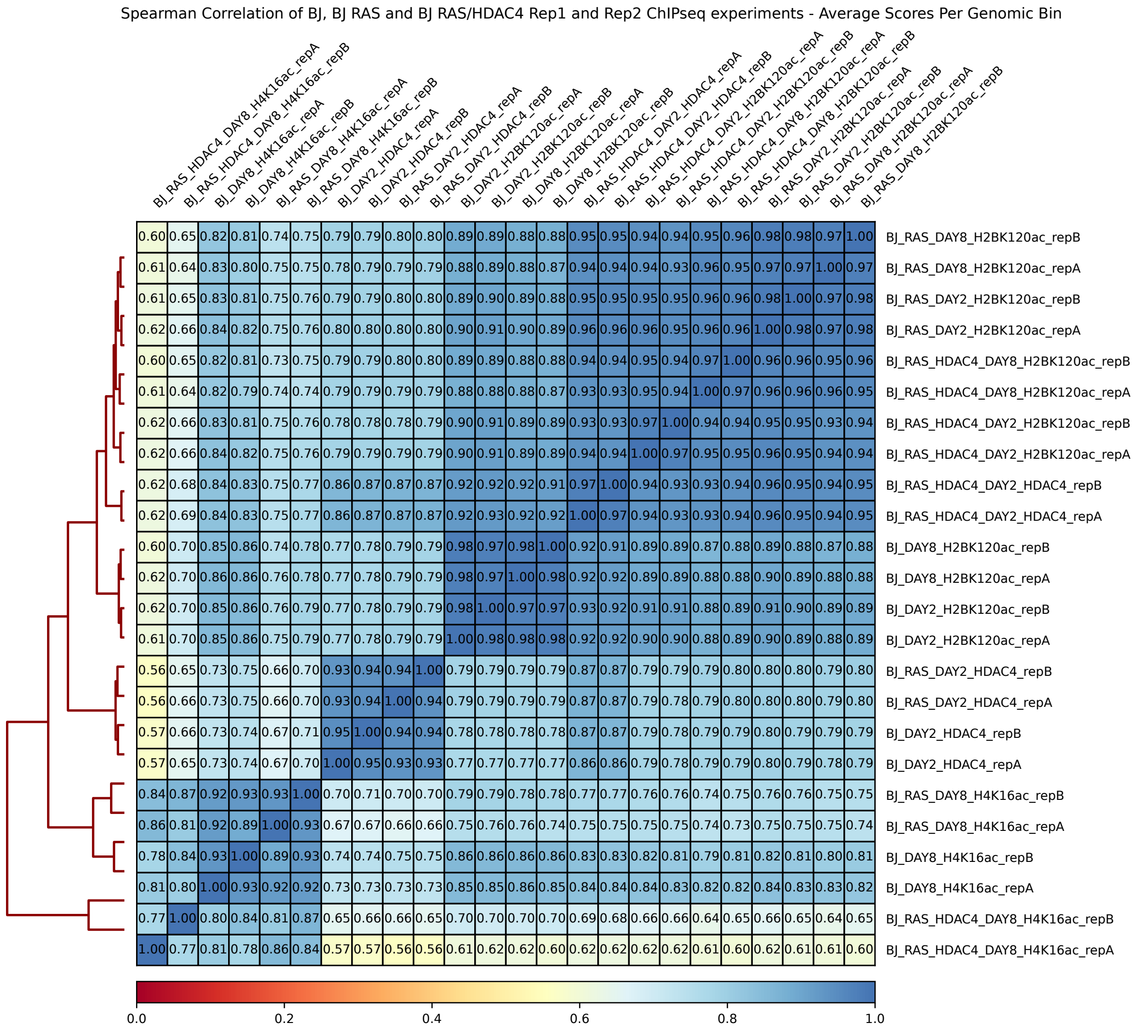

Figure S11. Consistency of data between replicates in ChIP-seq experiments.
Heatmap showing the Spearman correlation scores between all the replicate samples of the investigated chromatin markers in the three cell lines examined.

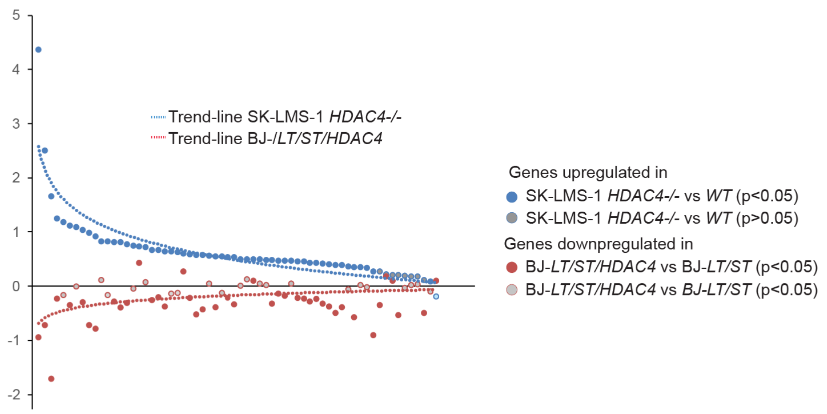

Figure S12. Genes under HDAC4 regulation.

Genomic loci displaying direct HDAC4 binding and belonging to KEGG “Cellular senescence” category (Figure 7D) were identified and their expression levels in SK-LMS-1 and BJ-*LT/ST/HDAC4* were plotted are log2(ratio) as indicated in the figure (GSE150427).

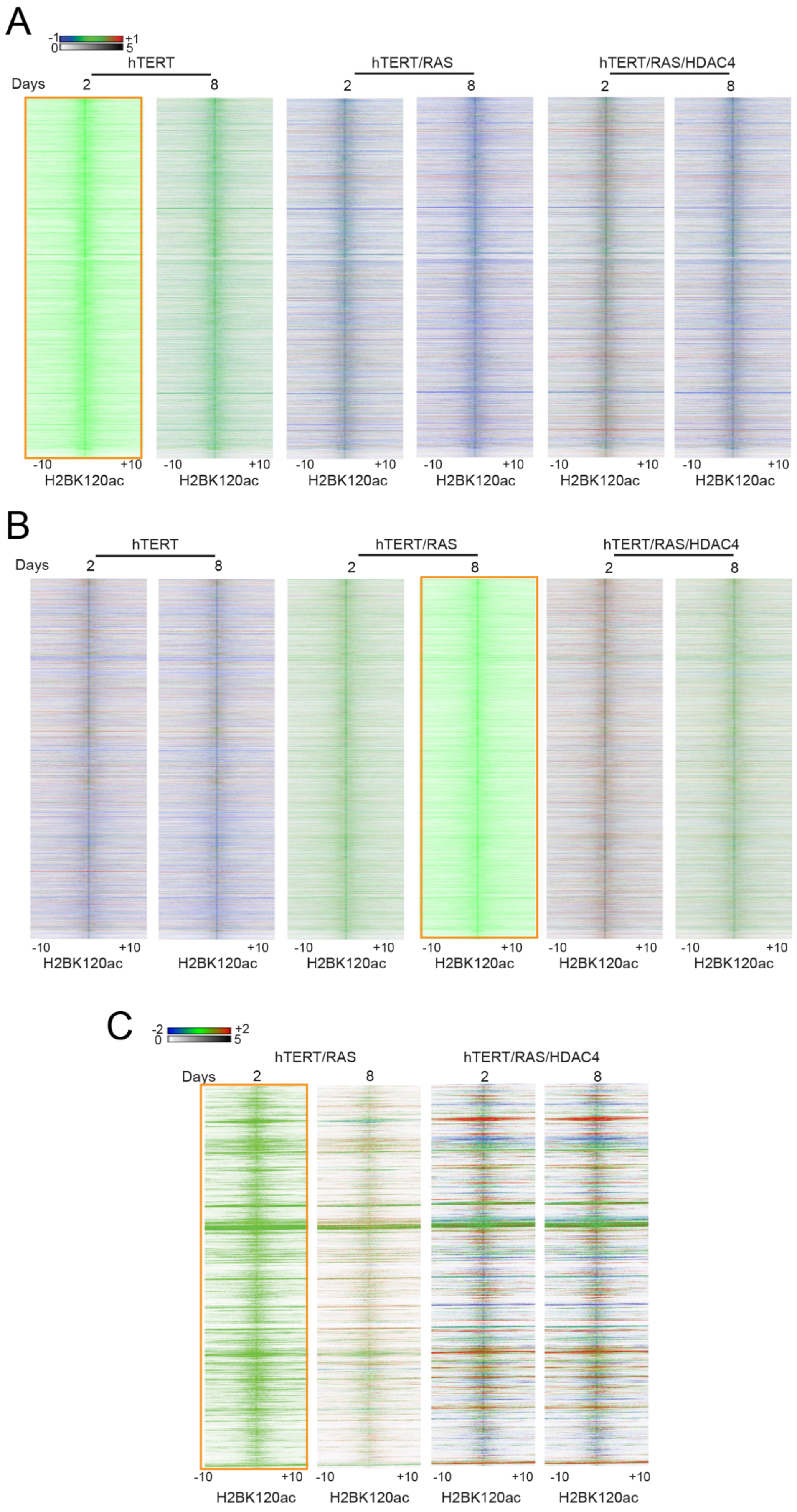
A
-1
+1
0
5
hTERT
hTERT/RAS
hTERT/RAS/HDAC4
Days
2
8
-10
+10
H2BK120ac
B
C
-2
+2

Figure S13. The genomic characterization of HDAC4 and H2BK120ac dynamics during OIS and DNA damage

A. Heatmap showing the intensity (represented in shades of red, blue and green) and density (proportional to color gradation) of H2BK120ac peaks in the indicated cell lines compared to BJ-*hTERT* at day 2. The signals are within 20kb of the center of the peaks identified in BJ-*hTERT* (replicate B).

B. Heatmap depicting the intensity (represented in shades of red, blue and green) and density (proportional to the color gradation) of H2BK120ac peaks in the indicated cell lines compared to BJ-*hTERT/RAS* at day 8. Signals are within 20kb of the center of the peaks identified in BJ-*hTERT/RAS* (replicate B).

C. Heatmap depicting the intensity (represented as shades of red, blue and green) and density (proportional to the color gradation) of H2BK120ac peaks in the indicated cell lines compared to BJ-*hTERT/RAS* at day 2. Signals are within 20kb of the center of HDAC4 binding in BJ-*hTERT/RAS/HDAC4* cells (replicate A).

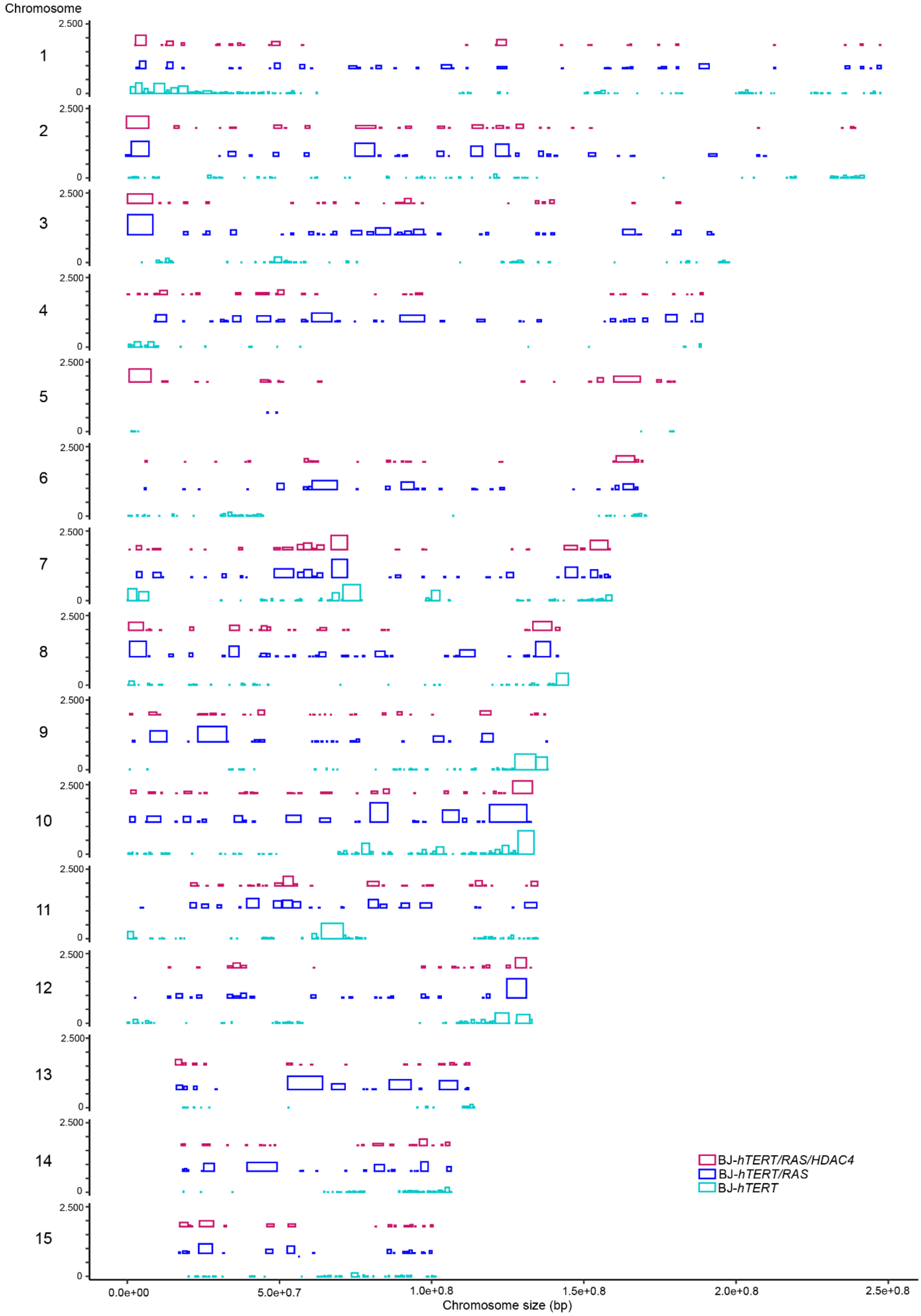

Chromosome
2.500
0
1
2
3
4
5
6
7
8
9
10
11
12
13
14
15
BJ-hTERT/RAS/HDAC4
BJ-hTERT/RAS
BJ-hTERT
0.0e+00
5.0e+0.7
1.0e+0.8
1.5e+0.8
2.0e+0.8
2.5e+0.8
Chromosome size (bp)

Figure S14. Chromosomal distribution of the γH2AX signal between BJ-*hTERT*, BJ-*hTERT/RAS* and BJ-*hTERT/RAS/HDAC4* cells induced with 4OHT for 8 days in Replicate A. (Chromosomes 1-15). Enriched peak counts are shown.

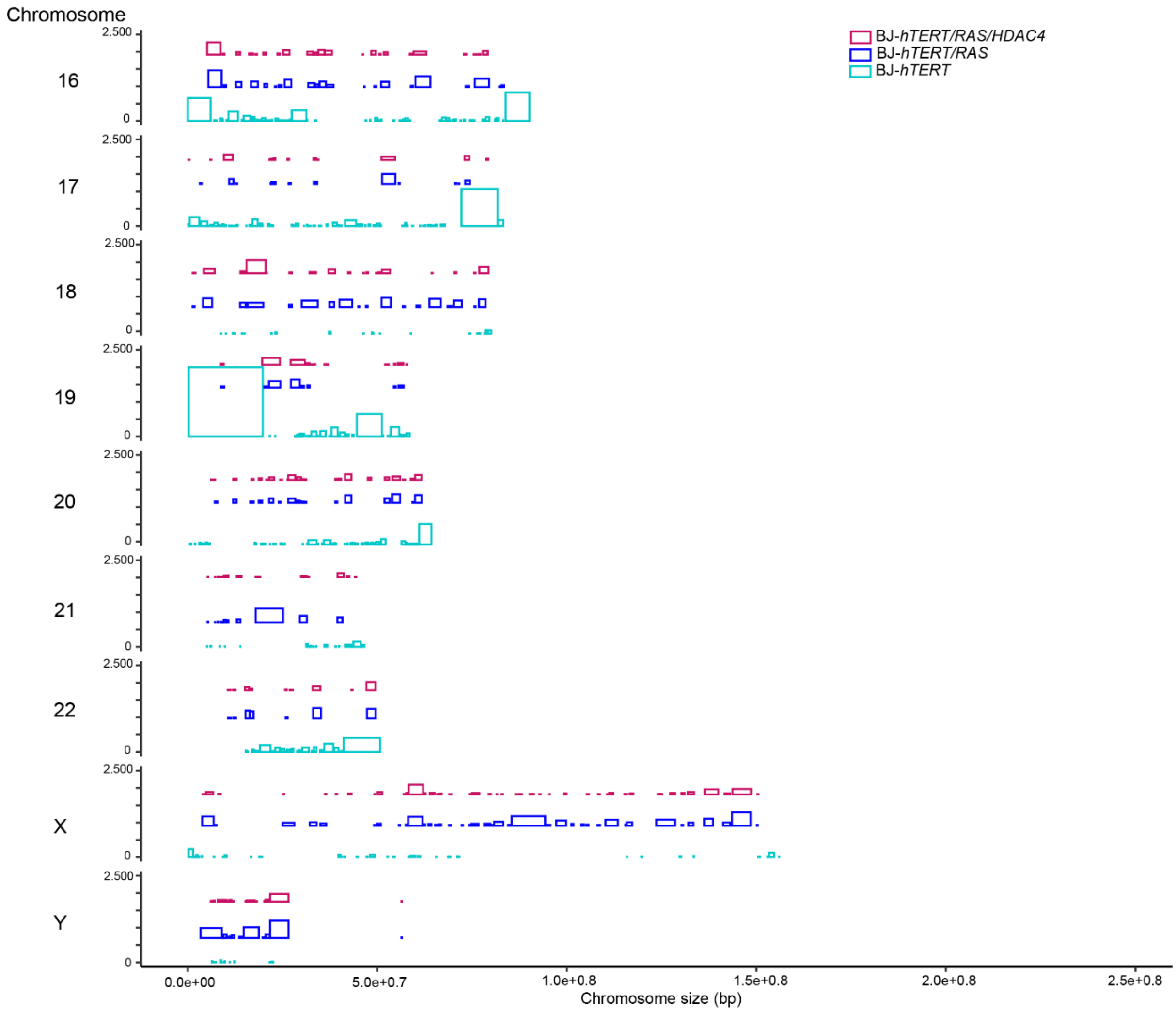

Figure S15. Chromosomal distribution of the γH2AX signal between BJ-*hTERT*, BJ-*hTERT/RAS* and BJ-*hTERT/RAS/HDAC4* cells induced with 4OHT for 8 days in Replicate A. (Chromosomes 16-Y). Enriched peak counts are shown.

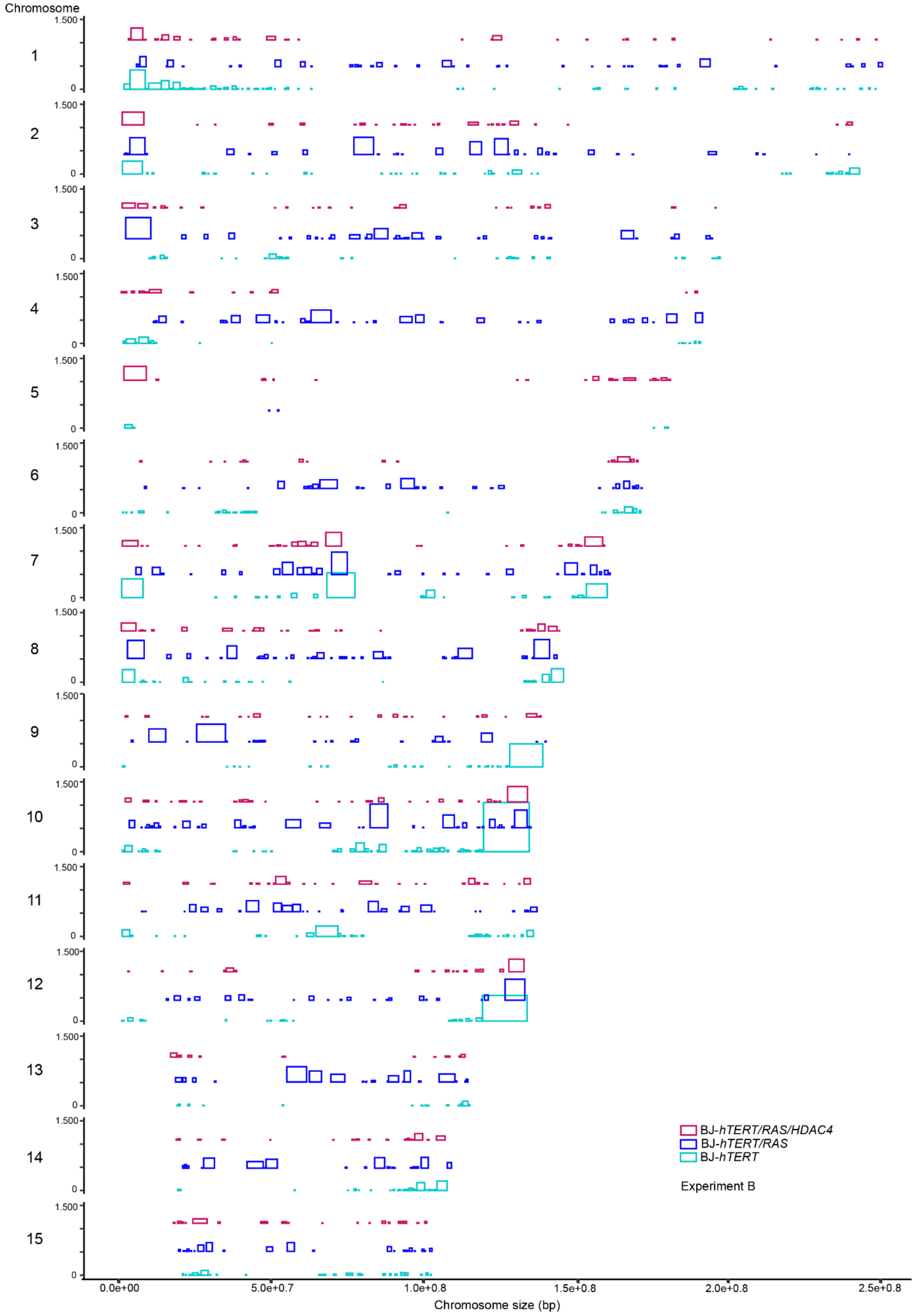

Chromosome
1.500
0
1
2
3
4
5
6
7
8
9
10
11
12
13
14
15
0.0e+00
5.0e+0.7
1.0e+0.8
1.5e+0.8
2.0e+0.8
2.5e+0.8
Chromosome size (bp)
BJ-hTERT/RAS/HDAC4
BJ-hTERT/RAS
BJ-hTERT
Experiment B

Figure S16. Chromosomal distribution of the γH2AX signal between BJ-*hTERT*, BJ-*hTERT/RAS* and BJ-*hTERT/RAS/HDAC4* cells induced with 4OHT for 8 days in Replicate B. (Chromosomes 1-15). Enriched peak counts are shown.

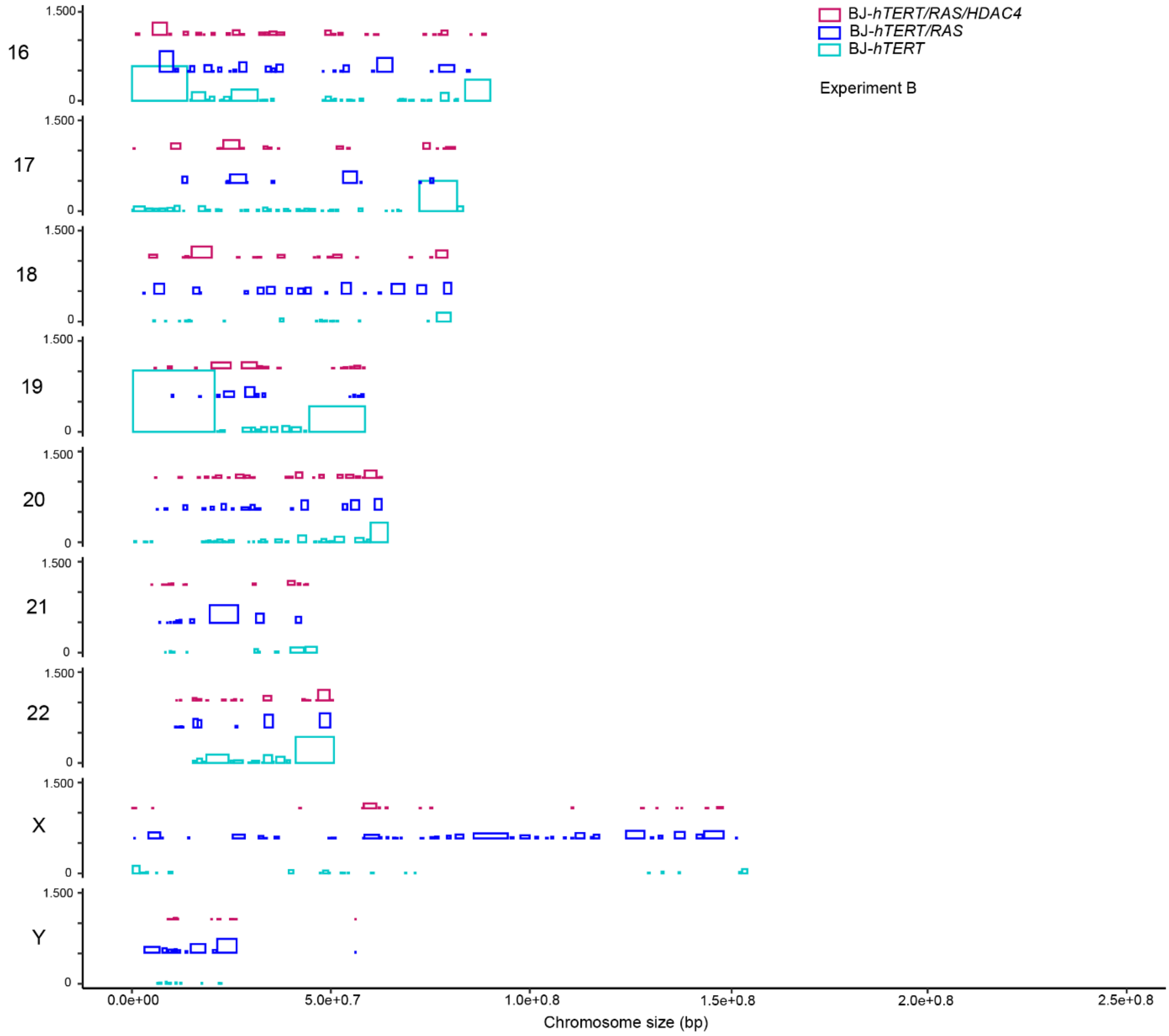

Figure S17. Chromosomal distribution of the γH2AX signal between BJ-*hTERT*, BJ-*hTERT/RAS* and BJ-*hTERT/RAS/HDAC4* cells induced with 4OHT for 8 days in Replicate B. (Chromosomes 1-15). Enriched peak counts are shown.

Other Supplementary Materials for this manuscript include:

Movie S1. Accumulation of DNA damage in SK-LMS-1$^{HDAC4\text{-/-}/HDAC4PAM\text{-}ER}$ cells expressing H2B-GFP and Apple-TP53BP1 grown in the presence of 4OHT.

Movie S2. Accumulation of DNA damage in SK-LMS-1$^{HDAC4\text{-/-}/HDAC4PAM\text{-}ER}$ cells expressing H2B-GFP and Apple-TP53BP1 in the absence of 4OHT.

Tables S1. SEs activated by HDAC4 depletion in cells SK-LMS-1$^{HDAC4\text{-/-}/HDAC4PAM\text{-}ER}$ cells at the indicated hours after 4OHT removal.

Tables S2. Analysis of changes in mRNA expression of genes involved in DNA repair, as defined by the GO categories (DNA damge + DNA repair and Homologous repair) in LMS cells SK-LMS-1 and SK-U-T-1 knocked out for HDAC4. Data are from GSE150427 and GSE132569 respectively.

Table S3. Mass spectrometry analysis (Co-IP-MS) of HDAC4 complexes in SK-LMS-1$^{HDAC4/GFP\ PITCH}$ cells.

Table S4. Genomic distribution and total number of peaks from H2BK120ac ChIP-seq experiments in the indicated cell lines.

Table S5. Overlap between genomic regions for H2BK120ac signals in the indicated cell lines. 1nt overlap was selected for the analysis.

Table S6. The length of the genomic domains of γH2AX in the indicated cell lines.

Table S7. A list of the antibodies and oligonucleotides that were used in this study.
