# Supplementary figures and images for: HDAC4 influences the DNA damage response and counteracts senescence by assembling with HDAC1/HDAC2 to control H2BK120 acetylation and homology-directed repair
Source: Nucleic Acids Res. 2024 Jun 14;52(14):8218–40. doi: 10.1093/nar/gkae501 (PMC11317144; doi:10.1093/nar/gkae501)

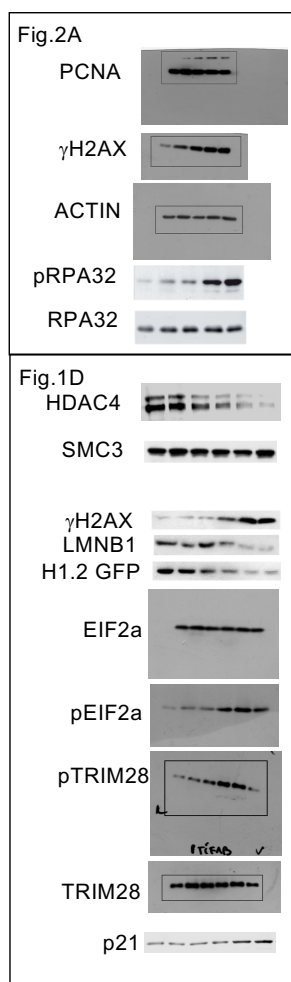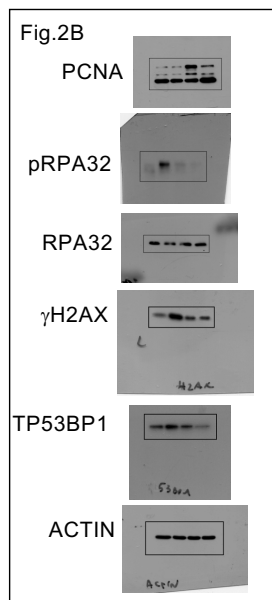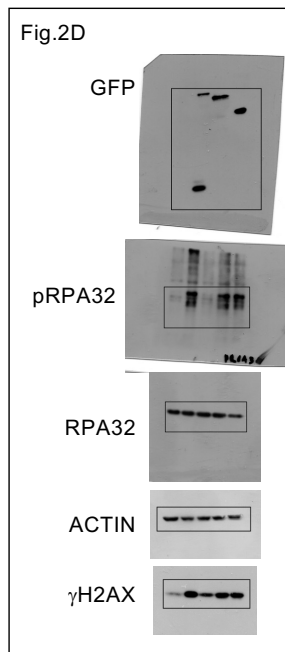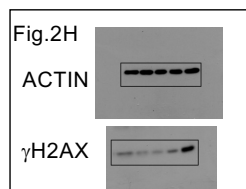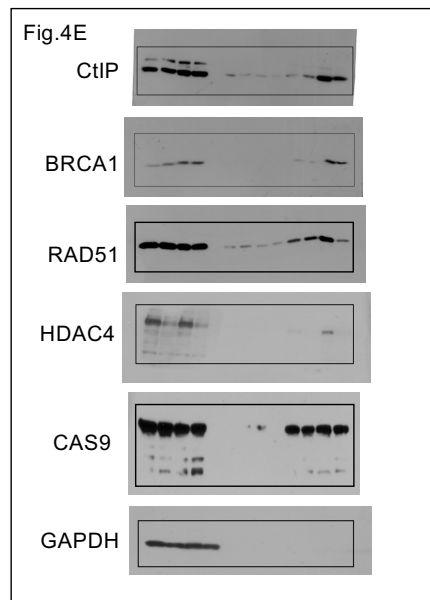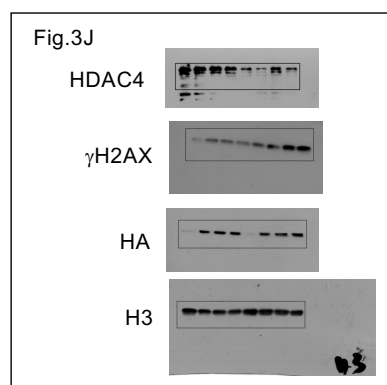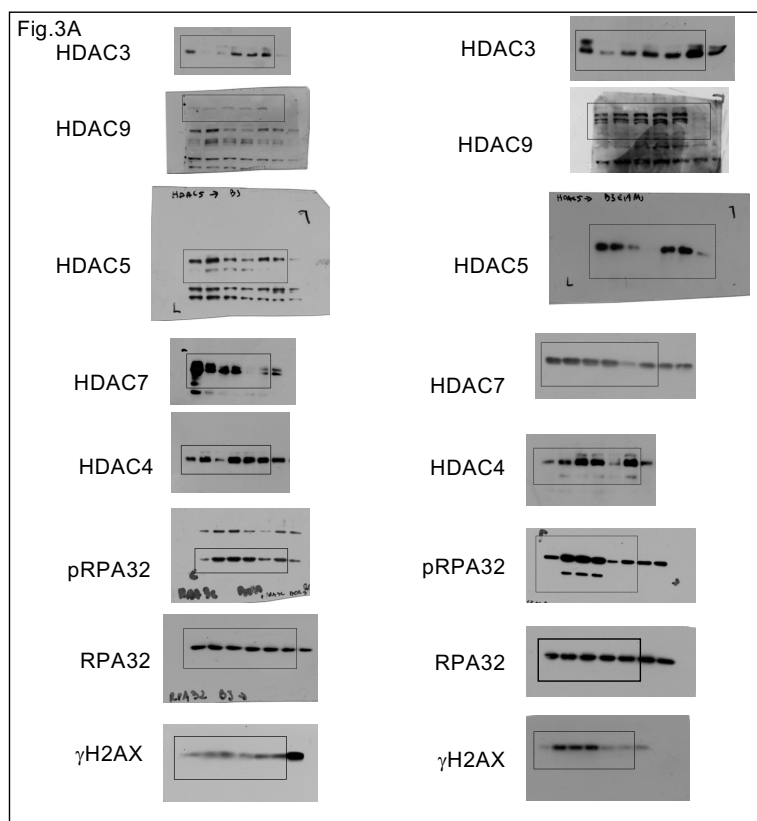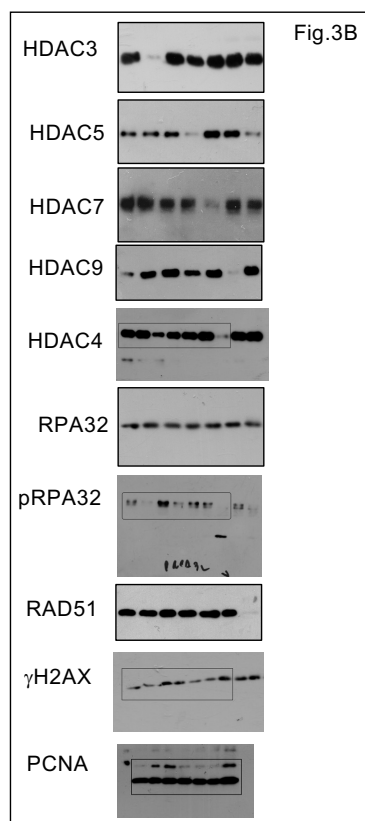

Fig. 5A, B

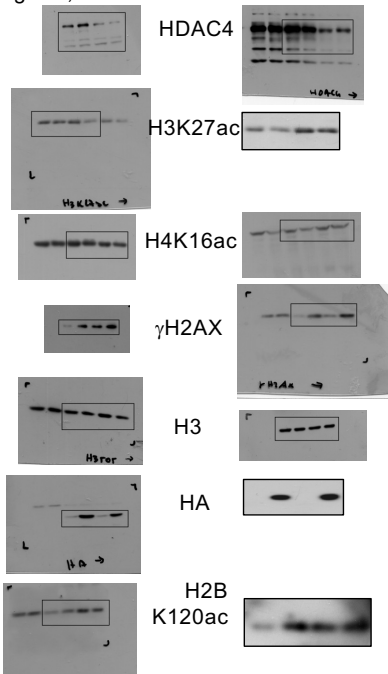

Fig. 5H

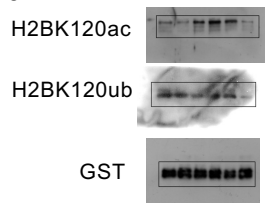

Fig. 5C

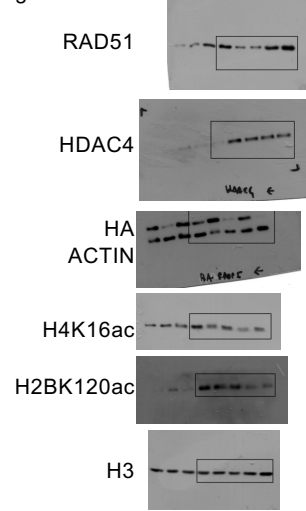

Fig. 5K

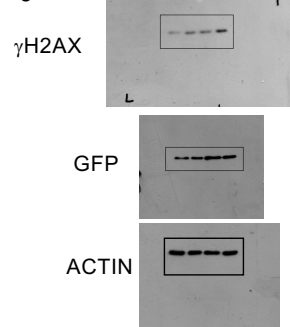

Fig. 5D

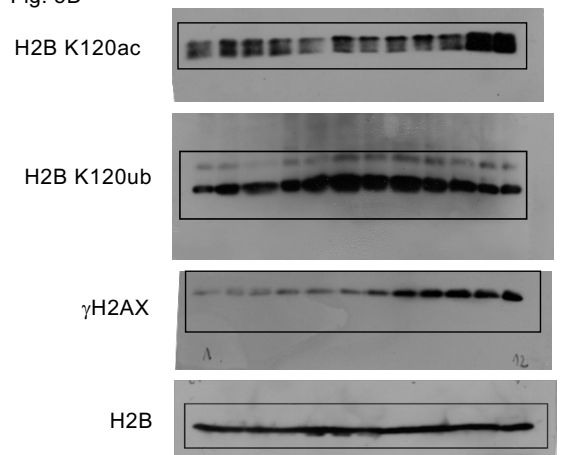

Fig. 5I

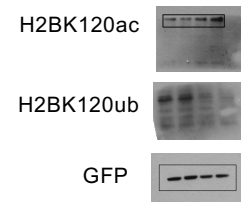

Fig. 5M

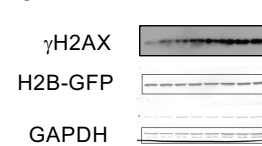

Fig. 5J

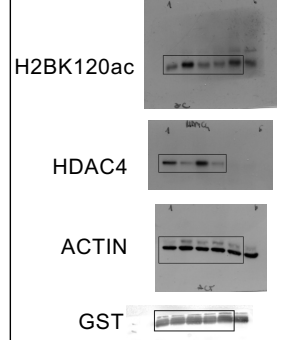

Fig. 6A

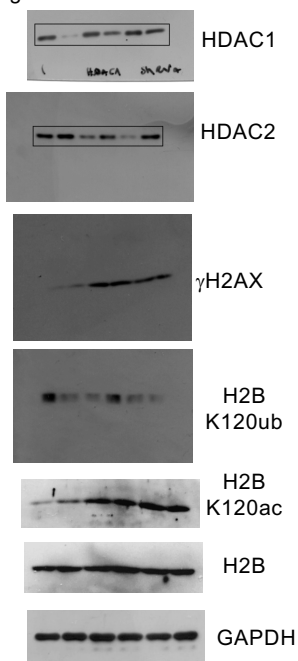

Fig. 6B

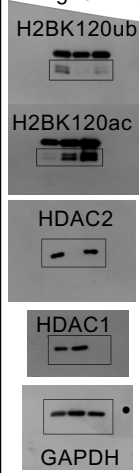

Fig. 6D

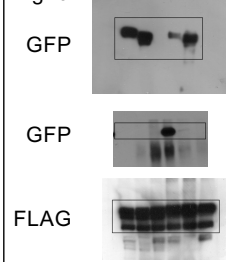

Fig. 6E

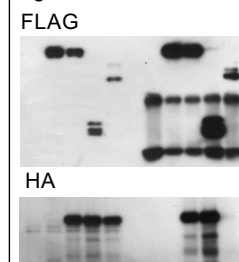

Fig. 6H

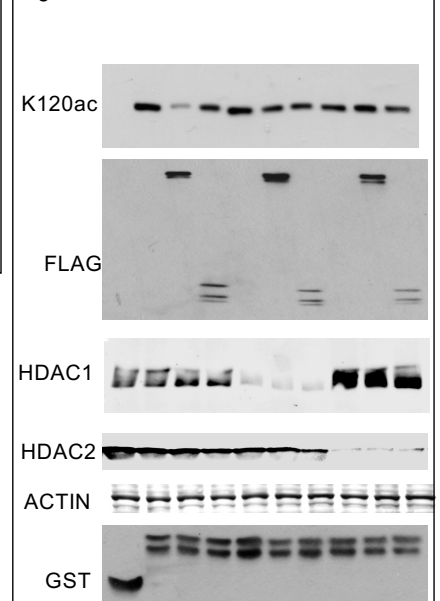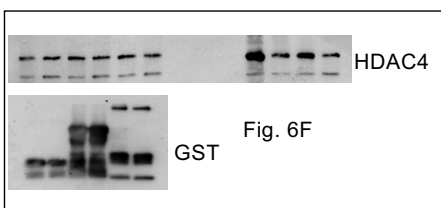

Fig. 6F

Fig. 6C

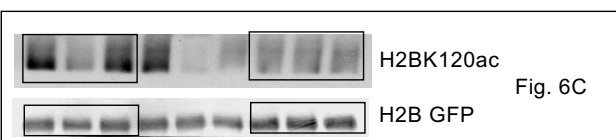

Fig. 7A

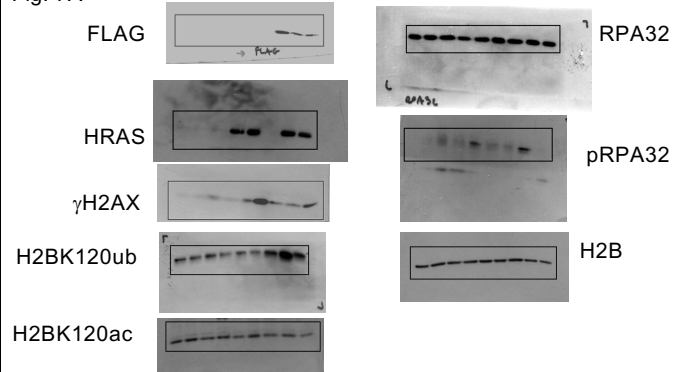

Fig. 6G

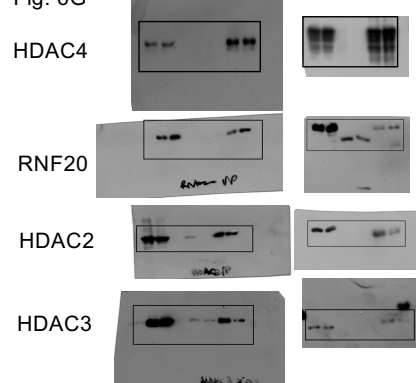

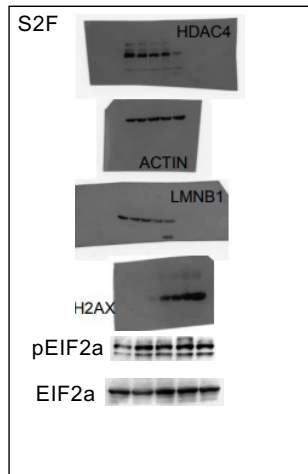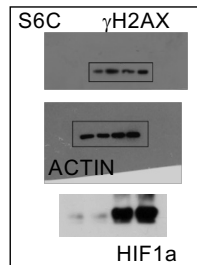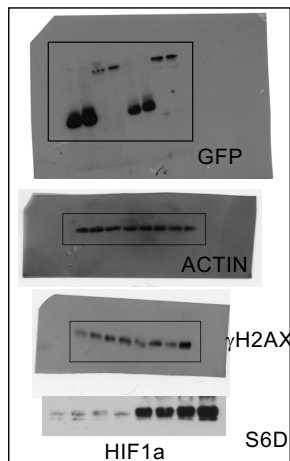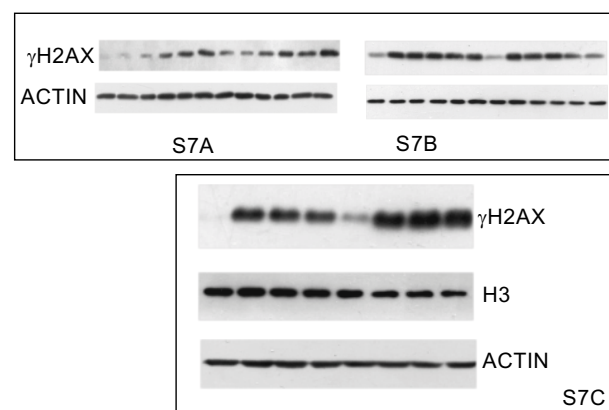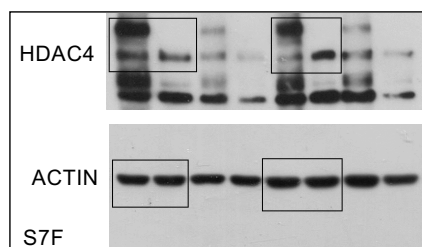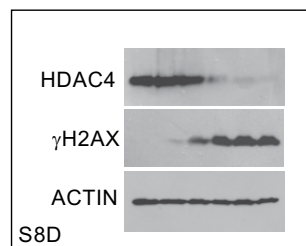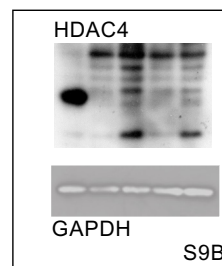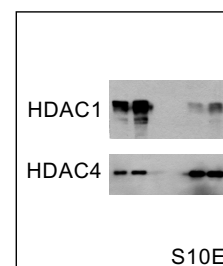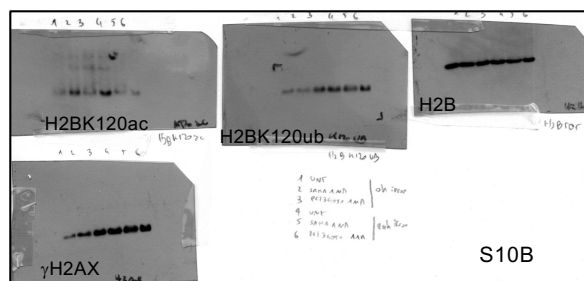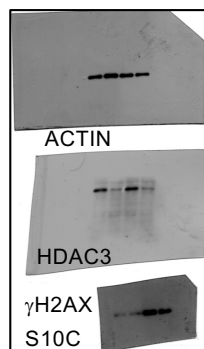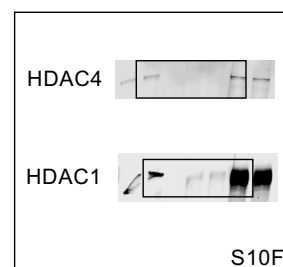

Supplement: gkae501_Supplemental_Files [file gkae501_supplemental_files.zip › Original scan of immunoblots.pdf]
